# Prasinezumab slows motor progression in rapidly progressing early-stage Parkinson’s disease

**DOI:** 10.1038/s41591-024-02886-y

**Published:** 2024-04-15

**Authors:** Gennaro Pagano, Kirsten I. Taylor, Judith Anzures Cabrera, Tanya Simuni, Kenneth Marek, Ronald B. Postuma, Nicola Pavese, Fabrizio Stocchi, Kathrin Brockmann, Hanno Svoboda, Dylan Trundell, Annabelle Monnet, Rachelle Doody, Paulo Fontoura, Geoffrey A. Kerchner, Patrik Brundin, Tania Nikolcheva, Azad Bonni, PASADENA Investigators, Prasinezumab Study Group

**Affiliations:** 1 Roche Pharma Research and Early Development (pRED), Neuroscience and Rare Diseases Discovery and Translational Area, Roche Innovation Center Basel, Basel, Switzerland; 2 University of Exeter Medical School, London, UK; 3 Roche Products Ltd, Welwyn Garden City, UK; 4 Department of Neurology, Northwestern University Feinberg School of Medicine, Chicago, IL USA; 5 Institute for Neurodegenerative Disorders, New Haven, CT USA; 6 Department of Neurology, Montreal Neurological Institute, McGill University, Montreal, Quebec Canada; 7 Clinical Ageing Research Unit, Newcastle University, Newcastle upon Tyne, UK; 8 The Institute for Research and Medical Care (IRCCS) San Raffaele Pisana, University San Raffaele Roma, Rome, Italy; 9 Hertie Institute for Clinical Brain Research and German Center for Neurodegenerative Diseases (DZNE), University of Tübingen, Tübingen, Germany; 10 Roche Diagnostics GmbH, Penzberg, Germany; 11 F. Hoffmann-La Roche Ltd, Basel, Switzerland

**Keywords:** Parkinson's disease, Drug development

## Abstract

Prasinezumab, a monoclonal antibody that binds aggregated α-synuclein, is being investigated as a potential disease-modifying therapy in early-stage Parkinson’s disease. Although in the PASADENA phase 2 study, the primary endpoint (Movement Disorder Society Unified Parkinson’s Disease Rating Scale (MDS-UPDRS) sum of Parts I + II + III) was not met, prasinezumab-treated individuals exhibited slower progression of motor signs than placebo-treated participants (MDS-UPDRS Part III). We report here an exploratory analysis assessing whether prasinezumab showed greater benefits on motor signs progression in prespecified subgroups with faster motor progression. Prasinezumab’s potential effects on disease progression were assessed in four prespecified and six exploratory subpopulations of PASADENA: use of monoamine oxidase B inhibitors at baseline (yes versus no); Hoehn and Yahr stage (2 versus 1); rapid eye movement sleep behavior disorder (yes versus no); data-driven subphenotypes (diffuse malignant versus nondiffuse malignant); age at baseline (≥60 years versus <60 years); sex (male versus female); disease duration (>12 months versus <12 months); age at diagnosis (≥60 years versus <60 years); motor subphenotypes (akinetic–rigid versus tremor-dominant); and motor subphenotypes (postural instability gait dysfunction versus tremor-dominant). In these subpopulations, the effect of prasinezumab on slowing motor signs progression (MDS-UPDRS Part III) was greater in the rapidly progressing subpopulations (for example, participants who were diffuse malignant or taking monoamine oxidase B inhibitors at baseline). This exploratory analysis suggests that, in a trial of 1-year duration, prasinezumab might reduce motor progression to a greater extent in individuals with more rapidly progressing Parkinson’s disease. However, because this was a post hoc analysis, additional randomized clinical trials are needed to validate these findings.

## Abstract (web summary)

An exploratory analysis of the 1-year clinical trial PASADENA in individuals with early-stage Parkinson’s disease suggests that prasinezumab might reduce motor signs progression to a greater extent in those with more rapidly progressing disease.

## Main

Pathological α-synuclein is considered to be the hallmark of Parkinson’s disease (PD) and several lines of evidence suggest a role for α-synuclein aggregates, and their propagation between neurons, in the pathogenesis of PD progression^1^.

Prasinezumab is the first experimental therapeutic monoclonal antibody designed to bind aggregated α-synuclein^2,3^. The effect of prasinezumab was investigated in individuals with early-stage PD in the PASADENA phase 2 study (NCT03100149)^4^. Part I of the study was double-blind and included 316 individuals with early-stage PD randomized 1:1:1 to intravenous infusions of placebo, prasinezumab 1,500 mg or prasinezumab 4,500 mg every 4 weeks for 52 weeks. Participants were stratified for age at baseline (<60 years versus ≥60 years), sex at birth (male versus female) and use of monoamine oxidase B (MAO-B) inhibitors at baseline (yes versus no). Except for the use of MAO-B inhibitors, other symptomatic medications for PD, including levodopa and dopamine agonists, were not allowed at baseline, and their use was discouraged for the duration of the double-blind period of the study, unless absolutely necessary. In those cases, a prior-to-start of symptomatic treatment visit was performed to collect MDS-UPDRS^5^ scores before symptomatic medication was commenced.

PASADENA revealed no differences in the change from baseline at week 52 in the sum of Parts I + II + III of the MDS-UPDRS^5^, the primary endpoint of the study^4^. Similarly, the results of another phase 2 study testing a different monoclonal antibody directed against aggregated α-synuclein (cinpanemab) were also negative on multiple endpoints^6^. However, in PASADENA, prasinezumab-treated individuals exhibited less progression on MDS-UPDRS Part III. No differences were found for MDS-UPDRS Parts I and II^4^, but these subscales of the MDS-UPDRS are unlikely to change over a 1-year period, as observed in the Parkinson’s Progression Marker Initiative (PPMI), a larger observational study of PD natural progression. In fact, over the first 52 weeks, the PPMI cohort exhibited a clinically meaningful decline in MDS-UPDRS Part III scores, but minimal changes in MDS-UPDRS Parts II and I, which fell below the thresholds for clinical meaningfulness^7–9^. Although the PASADENA MDS-UPDRS findings should be considered preliminary because there was no provision for correcting confidence interval (CI) widths for multiple comparisons, they are consistent with the idea that a potential treatment effect on disease progression can only be demonstrated when patients progress sufficiently on the endpoint of interest.

We therefore hypothesized that prasinezumab might show a greater effect in subpopulations with rapidly progressing disease, as measured by MDS-UPDRS Part III, compared with more slowly progressing subpopulations, because greater progression (with comparable variability of progression) is expected to increase the signal-to-noise ratio (degree of change over time) and the likelihood of revealing a potential treatment effect^10^. The initial PASADENA protocol^10^ included six prespecified primary subpopulations and nine prespecified exploratory subpopulations, defined by factors known to be associated with faster progression, such as MAO-B inhibitors at baseline (versus treatment-naive), Hoehn and Yahr stage 2 (versus stage 1) and diffuse malignant phenotypes (versus nondiffuse malignant phenotypes) (Table 1).

**Table 1:** Prespecified primary and exploratory subpopulations

Subpopulation	Category	Disease progression	≥20% of patients
**Primary subpopulations**			
MAO-B inhibitor at baseline	Yes	Faster	Yes
	No	Slower	Yes
Hoehn and Yahr stage	2	Faster	Yes
	1	Slower	Yes
RBDSQ	≥5	Faster	Yes
	<5	Slower	Yes
Data-driven subphenotypes	Diffuse malignant	Faster	Yes
	Nondiffuse malignant	Slower	Yes
α-synuclein skin (staining by IHC on skin biopsy sections at baseline)^a^	Yes	Faster	No
	No	Slower	No
DaT-SPECT SBR (ipsilateral putamen)^b^	Very abnormal	Faster	No
	Abnormal	Slower	No
**Exploratory subpopulations**			
Age at baseline	≥60 years	Faster	Yes
	<60 years	Slower	Yes
Sex	Male	Faster	Yes
	Female	Slower	Yes
Disease duration	>12 months	Faster	Yes
	<12 months	Slower	Yes
Age at diagnosis	≥60 years	Faster	Yes
	<60 years	Slower	Yes
Atrophy in the nucleus basalis of Meynert	Yes	Faster	No
	No	Slower	No
MoCA total score	<22	Faster	No
	>22	Slower	No
*GBA* mutation^c^	Yes	Faster	No^b^
	No	Slower	No^b^
Motor subphenotypes I	Akinetic–rigid	Faster	Yes
	Tremor-dominant	Slower	Yes
Motor subphenotypes II	PIGD	Faster	Yes
	Tremor-dominant	Slower	Yes

^a^Note that these data may be confounded by technical pre-processing issues.

^b^Defined on the baseline data with a validated cutoff of 0.6.

^c^For the glucocerebrosidase (*GBA*) mutation subgroup, a 15% sample cutoff was prespecified because very few participants were expected to carry the mutation.

DaT-SPECT, dopamine transporter-single-photon emission computed tomography; IHC, immunohistochemistry; MAO-B, monoamine oxidase-B; MoCA, Montreal Cognitive Assessment; PIGD, postural instability gait dysfunction; RBDSQ, Rapid Eye Movement Sleep Behavior Disorder Screening Questionnaire; SBR, striatal binding ratio.

In this report, we describe the effect of prasinezumab on disease progression as quantified by the MDS-UPDRS Part I, II and III scores, focusing on: (1) the subpopulation taking stable doses of MAO-B inhibitors at baseline, and (2) those with the other prespecified indicators of faster progression. Only subpopulations containing at least 20% of patients from the modified intention-to-treat (mITT) population at baseline were included in the final analyses presented in this article.

## Results

A total of 443 individuals were screened for participation in the PASADENA study, and 316 were enrolled: 105 were assigned to placebo, 105 to prasinezumab 1,500 mg and 106 to prasinezumab 4,500 mg (ref. ^4^).

### Baseline characteristics of the participants

Two of the six prespecified primary subpopulations (ɑ-synuclein skin positive on skin biopsy sections at baseline and dopamine transporter single-photon emission computed tomography (DaT-SPECT) striatal binding ratio (SBR) ipsilateral putamen <0.6) included fewer than 20% of participants from the mITT population at baseline and were not further analyzed. Thus, only four primary subpopulations were included in the final analyses: (1) MAO-B inhibitors at baseline (yes versus no); (2) Hoehn and Yahr stage (2 versus 1); (3) rapid eye movement (REM) sleep behavior disorder (yes versus no); and (4) data-driven subphenotypes (diffuse malignant versus nondiffuse malignant). Similarly, of the nine exploratory subpopulations, six were included in the final analyses: (1) age at baseline (≥60 years versus <60 years); (2) sex (male versus female); (3) disease duration (>12 months versus <12 months); (4) age at diagnosis (≥60 years versus <60 years); (5) motor subphenotypes akinetic–rigid versus tremor-dominant; and (6) motor subphenotypes postural instability gait dysfunction (PIGD) versus tremor-dominant. All primary and exploratory subpopulations were defined a priori in the statistical analysis plan before database lock of the study. Baseline demographic and clinical characteristics of the placebo and prasinezumab groups were comparable in the subpopulations of patients who received MAO-B inhibitors at baseline versus those who were treatment-naive (Table 2 and Supplementary Table 1), and in the other primary and exploratory subpopulations included in the analyses (Supplementary Table 1).

**Table 2:** Baseline demographic and clinical characteristics of the whole study population, participants taking MAO-B inhibitors at baseline and those who were treatment-naive at baseline

	Whole population		MAO-B inhibitors		Treatment-naive	
	Placebo (*n* = 105)	Prasinezumab pooled (*n* = 211)	Placebo (*n* = 38)	Prasinezumab pooled (*n* = 77)	Placebo (*n* = 67)	Prasinezumab pooled (*n* = 134)
Age (years), mean (s.d.)	59.9 (8.7)	59.9 (9.3)	58.3 (8.4)	58.2 (9.4)	60.8 (8.8)	60.9 (9.2)
Sex (male), *n* (%)	71 (67.6)	142 (67.3)	24 (63.2)	50 (64.9)	47 (70.1)	92 (68.7)
Time since diagnosis (months), mean (s.d.)	9.95 (6.79)	10.19 (6.37)	11.93 (6.37)	11.98 (5.99)	8.83 (6.81)	9.17 (6.37)
Time since diagnosis ≤12 months, *n* (%)	72 (68.6)	147 (69.7)	22 (57.9)	47 (61.0)	50 (74.6)	100 (74.6)
Hoehn and Yahr stage, *n* (%)						
Stage I	20 (19.0)	58 (27.5)	7 (18.4)	25 (32.5)	13 (19.4)	33 (24.6)
Stage II	85 (81.0)	153 (72.5)	31 (81.6)	52 (67.5)	54 (80.6)	101 (75.4)
MDS-UPDRS total (sum of Parts I, II and III), mean (s.d.)	32.01 (12.98)	31.11 (12.70)	32.16 (12.01)	29.25 (11.90)	31.93 (13.58)	32.19 (13.06)
MDS-UPDRS Part I, mean (s.d.)	4.91 (3.71)	4.45 (3.88)	5.08 (3.82)	4.19 (3.15)	4.82 (3.67)	4.60 (4.25)
MDS-UPDRS Part II, mean (s.d.)	5.55 (4.09)	5.22 (4.03)	5.84 (4.25)	4.87 (3.69)	5.39 (4.01)	5.43 (4.21)
MDS-UPDRS Part III, mean (s.d.)	21.54 (9.11)	21.44 (8.97)	21.24 (8.77)	20.18 (8.87)	21.72 (9.36)	22.16 (8.98)
DaT-SPECT SBR^a^, mean (s.d.)	1.06 (0.30)	1.06 (0.34)	1.03 (0.30)	1.02 (0.31)	1.09 (0.29)	1.09 (0.35)
Estimated average change in MDS-UPDRS Part III per month before the study (points per month)	2.16	2.10	1.78	1.68	2.46	2.42

*n* is the number of participants contributing to summary statistics. Percentages are based on *n*.

^a^Ipsilateral putamen. DaT-SPECT, dopamine transporter-single-photon emission computed tomography; MAO-B, monoamine oxidase-B; MDS-UPDRS, Movement Disorder Society-Unified Parkinson’s Disease Rating Scale; s.d., standard deviation; SBR, striatal binding ratio.

### Motor sign progression in the prespecified subpopulations

Analyses were performed using two estimand strategies: the ‘hypothetical strategy’ assumes a scenario in which the events of start of symptomatic therapy or change in MAO-B inhibitor dose did not occur, and the ‘treatment policy strategy’ in which the treatment effect is estimated irrespective of symptomatic treatment start or changes in MAO-B inhibitor treatment (see Methods for further details).

**Fig. 1:**
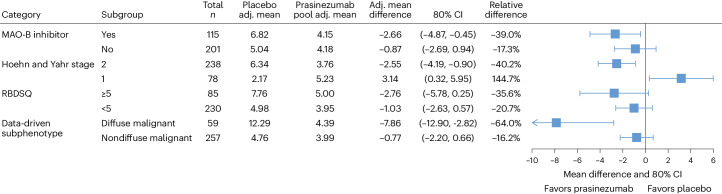
Forest plot of prasinezumab effects on motor progression as measured by the MDS-UPDRS Part III (hypothetical strategy) across the primary prespecified subpopulations. Adj., adjusted; MAO-B, monoamine oxidase B; MDS-UPDRS, Movement Disorder Society-sponsored revision of the Unified Parkinson’s Disease Rating Scale; RBDSQ, Rapid Eye Movement Sleep Behavior Disorder Screening Questionnaire. Error bars represent 80% confidence interval (CI).

The placebo groups in each prespecified rapidly progressing subpopulation declined faster than their nonrapidly progressing counterparts in motor function, as assessed by MDS-UPDRS Part III (Fig. 1 and Supplementary Fig. 1); for example, mean (s.e.) changes (with the hypothetical strategy) from baseline to week 52 were 6.82 (1.37) points in participants taking MAO-B inhibitors at baseline versus 5.04 (1.16) points in those who were treatment-naive; 6.34 (1.04) points in participants with Hoehn and Yahr stage 2 versus 2.17 (1.84) points in those with stage 1; 7.04 (1.30) points in those who were ≥60 years at baseline versus 3.83 (1.25) points in those aged <60 years; and 8.40 (1.59) points in participants with the motor subphenotype of PIGD versus 4.70 (1.11) points in those with tremor-dominant phenotype (Fig. 1, Table 3, Supplementary Fig. 1 and Supplementary Table 2).

**Table 3:** Change from baseline at week 52 in the subpopulations of participants taking MAO-B inhibitors at baseline and those who were treatment-naive at baseline

	Placebo			Prasinezumab pooled					
	MAO-B (*n* = 38)	Treatment-naive (*n* = 67)	All (*n* = 105)	MAO-B (*n* = 77)			Treatment-naive (*n* = 134)		
	Adjusted mean (s.e.)	Adjusted mean (s.e.)	Adjusted mean (s.e.)	Difference in adjusted means (s.e.)	80% CI	% RR	Difference in adjusted means (s.e.)	80% CI	% RR
**MDS-UPDRS Part III**									
Hypothetical strategy^a^	6.82 (1.371) *n* = 28	5.04 (1.163) *n* = 48	5.57 (0.897) *n* = 76	−2.66 (1.713) *n* = 55	−4.87, −0.45	−39.0	−0.87 (1.411) *n* = 92	−2.69, 0.94	−17.3
Treatment policy OFF^b^	4.79 (1.214) *n* = 36	3.10 (1.048) *n* = 65	3.56 (0.800) *n* = 101	−2.60 (1.476) *n* = 76	−4.51, −0.70	−54.3	0.53 (1.267) *n* = 125	−1.10, 2.16	+17.1
Treatment policy ON^b^	4.18 (1.248) *n* = 38	2.01 (1.109) *n* = 67	2.66 (0.840) *n* = 105	−2.60 (1.532) *n* = 76	−4.57, −0.63	−62.2	0.32 (1.340) *n* = 130	−1.40, 2.04	+15.9
**MDS-UPDRS Part II**									
Hypothetical strategy^a^	2.40 (0.635) *n* = 28	2.89 (0.467) *n* = 48	2.75 (0.373) *n* = 76	0.20 (0.773) *n* = 55	−0.80, 1.20	+8.3	0.10 (0.567) *n* = 92	−0.63, 0.83	+3.5
Treatment policy^b^	1.21 (0.592) *n* = 38	1.63 (0.438) *n* = 67	1.47 (0.353) *n* = 105	0.22 (0.721) *n* = 76	−0.71, 1.15	+18.2	0.25 (0.531) *n* = 129	−0.43, 0.93	+15.3
**MDS-UPDRS Part I**									
Hypothetical strategy^a^	1.28 (0.466) *n* = 28	0.38 (0.371) *n* = 48	0.77 (0.295) *n* = 76	−0.44 (0.567) *n* = 55	−1.17, 0.29	−34.4	0.30 (0.447) *n* = 92	−0.27, 0.88	+78.9
Treatment policy^b^	0.50 (0.452) *n* = 37	0.10 (0.375) *n* = 67	0.20 (0.292) *n* = 104	0.03 (0.549) *n* = 76	−0.68, 0.73	+6.0	0.65 (0.453) *n* = 125	0.06, 1.23	+650.0

^a^‘Hypothetical strategy’ assumes a scenario in which the events of start of symptomatic therapy or change in MAO-B inhibitor dose did not occur (performed for the mITT population).

^b^‘Treatment policy strategy’ in which the treatment effect is estimated irrespective of symptomatic treatment start or changes in MAO-B inhibitor treatment (performed for the intention-to-treat population). CI, confidence interval; MAO-B, monoamine oxidase-B; MDS-UPDRS, Movement Disorder Society-Unified Parkinson’s Disease Rating Scale; mITT, modified intention-to-treat; %RR, percent relative reduction; s.e., standard error.

### Participants with and without MAO-B inhibitors at baseline

**Fig. 2:**
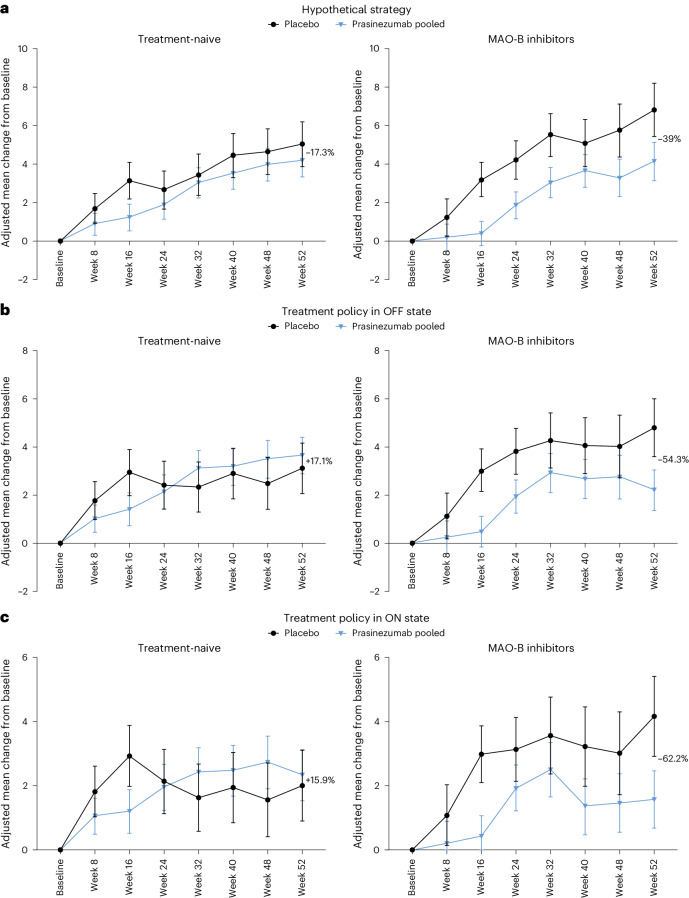
Prasinezumab effect on motor signs progression assessed using MDS-UPDRS Part III in participants taking MAO-B inhibitors at baseline and those who were treatment-naive at baseline. **a**, Hypothetical strategy. **b**, Treatment policy in OFF state. **c**, Treatment policy in ON state. The MDS-UPDRS endpoints were analyzed using mixed models for repeated measures. Error bars represent s.e. MAO-B inhibitors at baseline: prasinezumab pooled *n* = 77; placebo *n* = 38; treatment-naive at baseline: prasinezumab pooled *n* = 134; placebo *n* = 67. MAO-B, monoamine oxidase B; MDS-UPDRS, Movement Disorder Society-sponsored revision of the Unified Parkinson’s Disease Rating Scale.

With the hypothetical strategy, the mean (s.e.) change from baseline to week 52 in MDS-UPDRS Part III score in the entire PASADENA placebo population was 5.57 (0.90) points (Table 3). The corresponding mean (s.e.) change from baseline in the placebo group of the subpopulation of participants taking MAO-B inhibitors was 6.82 (1.37) points, compared with 5.04 (1.16) points in the placebo group of the treatment-naive subpopulation (Table 3 and Fig. 2a). The differences in adjusted means from baseline at week 52 in the pooled prasinezumab group versus placebo were −2.66 points (80% CI, −4.87, −0.45; relative reduction (RR), −39.0%) in the subpopulation of participants taking MAO-B inhibitors at baseline and −0.87 points (80% CI, −2.69, 0.94; RR, −17.3%) in the subpopulation of participants who were treatment-naive (Table 3 and Fig. 2a). Results from the groups receiving low and high doses of prasinezumab were comparable (Supplementary Tables 3 and 4).

Similar results were observed when MAO-B inhibitor subpopulation MDS-UPDRS Part III data were analyzed with the treatment policy strategy, both in OFF state (difference in adjusted means, −2.60 points; 80% CI, −4.51, −0.70; RR, −54.3%) (Table 3 and Fig. 2b) and in ON state (difference in adjusted means, −2.60 points; 80% CI, −4.57, −0.63; RR, −62.2%) (Table 3 and Fig. 2c).

Mean (s.e.) changes (with the hypothetical strategy) from baseline to week 52 in MDS-UPDRS Part II and Part I scores in the entire PASADENA placebo population were 2.75 (0.37) and 0.77 (0.30) points, respectively (Table 3). The mean (s.e.) changes from baseline to week 52 in MDS-UPDRS Part II score in the placebo subpopulations who were treated with MAO-B inhibitors and treatment-naive at baseline were 2.40 (0.64) and 2.89 (0.47) points, respectively, and the corresponding values for MDS-UPDRS Part I score were 1.28 (0.47) and 0.38 (0.37) points, respectively (Table 3). No differences were found between the prasinezumab and the placebo groups on MDS-UPDRS Part II and Part I, using either the hypothetical or treatment policy strategies (Table 3).

### Participants with Hoehn and Yahr stage 1 and 2

With the hypothetical strategy, the mean change (s.e.) from baseline to week 52 in MDS-UPDRS Part III score in the placebo group of the Hoehn and Yahr stage 2 subpopulation was 6.34 (1.04) points, compared with 2.17 (1.84) points in the placebo group of the Hoehn and Yahr stage 1 subpopulation (Fig. 1 and Supplementary Table 2). The differences in adjusted means from baseline at week 52 in the prasinezumab group versus placebo were −2.55 points (80% CI, −4.19, −0.9; RR, −40.2%) in the Hoehn and Yahr stage 2 subpopulation and 3.14 points (80% CI, 0.32, 5.95; relative change, +144.7%) in the Hoehn and Yahr stage 1 subpopulation (Fig. 1 and Supplementary Table 2).

When the MDS-UPDRS Part III data for the Hoehn and Yahr stage 2 subpopulation were analyzed with the treatment policy, the differences in adjusted means in OFF state and ON state were −1.33 points (80% CI, −2.76, 0.11; RR, −35.1%) and −1.51 points (80% CI, −3.02, 0.00; RR, −54.1%), respectively (Supplementary Table 2).

The mean changes (s.e.) (with the hypothetical strategy) from baseline to week 52 in MDS-UPDRS Part II score in the placebo groups of the Hoehn and Yahr stage 2 and stage 1 subpopulations were 3.01 (0.43) and 1.68 (0.70) points, respectively, and the corresponding values for MDS-UPDRS Part I score were 1.05 (0.33) and −0.18 (0.65) points, respectively (Supplementary Table 2). No differences were found between the prasinezumab and the placebo groups on MDS-UPDRS Part II and Part I, using either the hypothetical strategy or treatment policy (Supplementary Table 2).

### Participants with and without REM sleep behavior disorder

With the hypothetical strategy, the mean change (s.e.) from baseline to week 52 in MDS-UPDRS Part III score in the placebo group of the subpopulation with REM sleep behavior disorder was 7.76 (2.01) points, compared with 4.98 (1.01) points in the placebo group of the subpopulation without REM sleep behavior disorder (Fig. 1 and Supplementary Table 2). The differences in adjusted means from baseline at week 52 in the prasinezumab group versus placebo were −2.76 points (80% CI, −5.78, 0.25; RR, −35.6%) in the subpopulation with REM sleep behavior disorder and −1.03 points (80% CI, −2.63, 0.57; RR, −20.7%) in the subpopulation without REM sleep behavior disorder (Fig. 1 and Supplementary Table 2).

When the MDS-UPDRS Part III data for the subpopulation with REM sleep behavior disorder were analyzed with the treatment policy, the differences in adjusted means in OFF state and ON state were −1.21 points (80% CI, −3.92, 1.49; RR, −22.2%) and −0.04 points (80% CI, −2.80, 2.71; RR, −1.1%), respectively (Supplementary Table 2).

The mean changes (s.e.) (with the hypothetical strategy) from baseline to week 52 in MDS-UPDRS Part II score in the placebo groups of the subpopulations with and without REM sleep behavior disorder were 2.19 (0.86) and 2.82 (0.42) points, respectively, and the corresponding values for MDS-UPDRS Part I score were 1.28 (0.60) and 0.69 (0.34) points, respectively (Supplementary Table 2). No differences were found between the prasinezumab and the placebo groups on MDS-UPDRS Part II and Part I, using either the hypothetical strategy or treatment policy (Supplementary Table 2).

### Participants with diffuse and nondiffuse malignant phenotypes

With the hypothetical strategy, the mean change (s.e.) from baseline to week 52 in MDS-UPDRS Part III score in the placebo group of the diffuse malignant subpopulation was 12.29 (3.45) points, compared with 4.76 (0.91) points in the placebo group of the nondiffuse malignant subpopulation (Fig. 1 and Supplementary Table 2). The differences in adjusted means from baseline at week 52 in the prasinezumab group versus placebo were −7.86 points (80% CI, −12.90, −2.82; RR, −64.0%) in the diffuse malignant subpopulation and −0.77 points (80% CI, −2.20, 0.66; RR, −16.2%) in the nondiffuse malignant subpopulation (Fig. 1 and Supplementary Table 2).

When the MDS-UPDRS Part III data for the diffuse malignant subpopulation were analyzed with the treatment policy, the differences in adjusted means in OFF state and ON state were −2.58 points (80% CI, −6.18, 1.02; RR, −49.9%) and −2.76 points (80% CI, −6.67, 1.15; RR, −72.4%), respectively (Supplementary Table 2).

The mean changes (s.e.) (with the hypothetical strategy) from baseline to week 52 in MDS-UPDRS Part II score in the placebo groups of the diffuse malignant and nondiffuse malignant subpopulations were 4.08 (1.53) and 2.47 (0.37) points, respectively, and the corresponding values for MDS-UPDRS Part I score were 1.59 (1.13) and 0.76 (0.29) points, respectively (Supplementary Table 2). No differences were found between the prasinezumab and the placebo groups on MDS-UPDRS Part II and Part I, using either the hypothetical strategy or treatment policy (Supplementary Table 2).

### Participants in the prespecified exploratory subpopulations

A greater beneficial effect for prasinezumab versus placebo was also shown in all prespecified exploratory subpopulations with more rapidly progressing disease compared with their nonrapidly progressing counterparts (Supplementary Fig. 1 and Supplementary Table 2); for example, the differences in adjusted means in MDS-UPDRS Part III scores (with the hypothetical strategy) for pooled prasinezumab versus placebo in participants with age at baseline ≥60 years and <60 years were −1.89 points (80% CI, −3.90, 0.12; RR, −26.9%) and −0.61 points (80% CI, −2.58, 1.36; RR, −15.9%), respectively (Supplementary Fig. 1 and Supplementary Table 2). Results from the groups receiving low and high doses of prasinezumab were comparable for the exploratory as well as the primary subpopulations (Supplementary Tables 3 and 4).

### Sensitivity analyses

At baseline, the subpopulations might have differences in baseline characteristics (for example, participants treated with MAO-B inhibitors had a longer disease duration, lower DaT-SPECT SBR and different proportions of patients with Hoehn and Yahr stage 1 and 2 compared with the treatment-naive population, whereas the MDS-UPDRS Parts I, II and III scores were comparable) (Table 1). Therefore, the primary analyses were repeated for all subpopulations including all baseline characteristics as covariates. These analyses resulted in the same pattern of results as the primary analyses (results not shown).

## Discussion

In this exploratory analysis of the PASADENA study, we found a consistent effect of prasinezumab in predefined rapidly progressing subpopulations, with prasinezumab-treated participants exhibiting less increase (worsening) in MDS-UPDRS Part III compared with participants treated with placebo. These findings might suggest that prasinezumab slows the progression of motor signs in individuals with characteristics usually associated with more rapid progression within a 1-year timeframe. Our observations also expand upon those of the original PASADENA study, in which the overall population of prasinezumab-treated individuals exhibited less progression on MDS-UPDRS Part III than those treated with placebo^4^. However, an additional study in an independent population is needed to confirm the hypothesis that prasinezumab slows motor progression in early-stage PD.

In the original PASADENA study, prasinezumab failed to meet the primary endpoint (change from baseline in MDS-UPDRS sum of Parts I + II + III)^4^. The MDS-UPDRS sum of Parts I + II + III is a global measure of PD, including motor signs rated by the clinicians (Part III), and motor (Part II) and nonmotor (Part I) signs reported by the patients^5^. Although no differences were found in MDS-UPDRS Part I and II scores in the PASADENA study, participants treated with prasinezumab exhibited slower progression of motor signs on the MDS-UPDRS Part III scale.

For a disease-modifying treatment to be able to exhibit a substantial effect (a slowing of progression), a meaningful degree of disease progression in the placebo group is necessary during the study period. Importantly, the PASADENA participants (both placebo-treated and active-treated) progressed minimally on the MDS-UPDRS Part I (<1 point) and Part II (<3 points) over the 52-week double-blind treatment period. Minimal changes in the progression of MDS-UPDRS Parts I or II (that is, changes below what is considered clinically meaningful) were also observed in other studies that included participants with early-stage PD, such as the PPMI study^7–9^ and the De Novo Parkinson study^11^. On average, MDS-UPDRS Parts I and Part II declined 0.9–1.2 points and 1.0–1.6 points, respectively, at week 52 in the PPMI cohort^7–9^, which was similar to participants in the De Novo Parkinson and PASADENA studies^4,11^. By contrast, both the prasinezumab-treated and placebo-treated PASADENA participants progressed on average by ~5 points on the MDS-UPDRS Part III, which was more than the minimum threshold for a clinically meaningful change (previously defined as 4.63 points)^9^. Based on the PASADENA original finding that prasinezumab-treated individuals exhibited a numerically reduced clinical decline in MDS-UPDRS Part III scores relative to placebo^4^, we explored here the hypothesis that prasinezumab might have greater effects in prespecified subpopulations that were expected to decline more rapidly in motor function. We focused on this hypothesis because greater progression (with comparable variability of progression) is expected to increase the signal-to-noise ratio and the likelihood of showing a potential treatment effect in MDS-UPDRS Part III.

We confirmed faster declines in the subpopulations expected to progress more rapidly on motor signs (as measured by a larger increase in MDS-UPDRS Part III) with comparable variabilities of change. All rapidly progressing subpopulations consistently showed a numerically greater prasinezumab effect compared with their nonrapidly progressing counterparts. Moreover, we demonstrated that the prasinezumab effect size might have been related to the speed of progression in the placebo group. For example, the diffuse malignant subpopulation showed an increase (worsening) of 12.29 points on MDS-UPDRS Part III in the placebo group, and a 64.0% RR of worsening in prasinezumab-treated versus placebo-treated participants. By contrast, the nondiffuse malignant subpopulation showed an increase (worsening) of 4.76 points on MDS-UPDRS Part III in the placebo group, and a 16.2% RR in worsening in prasinezumab versus placebo. These relationships indicate that longer observation periods may be required to detect potential effects of prasinezumab in more slowly progressing populations.

Notably, when viewing the data for the placebo group, the subpopulations that progressed faster on MDS-UPDRS Part III did not progress faster on MDS-UPDRS Parts II and I. This may suggest that the progression of motor signs (MDS-UPDRS Part III) precedes notable changes in both motor and nonmotor symptoms (MDS-UPDRS Parts II and I). A difference in the clinical rating of motor signs versus patient self-rating/awareness of motor symptoms can also explain the differences in progression between MDS-UPDRS Part III and MDS-UPDRS Parts II and I^12^. Much longer studies may be required to test the effect of potential disease-modifying treatments, such as prasinezumab, on progression of patient-reported motor symptoms, functional activity of daily living and progression of nonmotor symptoms. Moreover, it confirms that motor signs remain the most reliable endpoint of disease progression in early-stage PD, as also shown in other studies on prodromal PD^13^.

The results of these analyses should be interpreted with caution, given the small sample sizes of most of the subpopulations and the lack of correction for multiple comparisons. However, the subpopulation of people treated with MAO-B inhibitors represents ~40% of the whole population, and the use of MAO-B inhibitors at baseline was included as a stratification factor at randomization. A further limitation of the study is that it cannot be excluded that the use of MAO-B inhibitors might reflect the treating clinician’s/site’s preferred approach to managing recently diagnosed patients, rather than representing an indicator of rapid progression in all patients. Three nonmutually exclusive explanations may account for the potentially greater effect of prasinezumab in subpopulations with faster progression. First, the effect of prasinezumab might be more detectable in the faster-progressing subpopulation because of an increased signal-to-noise ratio (that is, degree of change over time) on clinician-rated scale assessments of motor signs progression. Second, prasinezumab might exert a synergistic effect in people taking symptomatic therapy, such as MAO-B inhibitors. Evidence from multiple laboratory models suggests that α-synuclein aggregates induce both presynaptic and postsynaptic defects before promoting degeneration of the dopaminergic nigrostriatal pathway^14^. These findings suggest that removal of aggregates might induce relatively rapid restoration of neuronal function, which could translate into benefits on motor functions in PD, and that the benefits would be particularly evident when other pharmacotherapies that directly promote dopaminergic neurotransmission are used concomitantly (for example, MAO-B inhibitors). This may explain why the treatment effect is larger in the treatment policy analysis of MDS-UPDRS Part III, both in OFF and ON states, when measures of MDS-UPDRS Part III of people who started levodopa or dopamine agonists during the study are included in the analysis (Fig. 2b,c). It is also worth noting that the putative effect of prasinezumab on motor signs might have been already evidenced within the first 16 weeks, in line with the hypothesis that removal of extracellular aggregates could improve neural signaling^14^. Third, those subpopulations that progressed faster on motor signs may have a larger amount or more widely distributed pathological aggregated α-synuclein in the brain at baseline and thus might have responded more to prasinezumab^10^. However, without a validated quantitative biomarker of in vivo pathological α-synuclein in the brain, this hypothesis cannot be tested.

A different clinical trial (the SPARK study; NCT03318523) explored the potential efficacy of cinpanemab in early-stage treatment-naive PD populations, another monoclonal anti-α-synuclein antibody^6^. In that trial, cinpanemab showed no effect on either the primary (MDS-UPDRS Parts I + II + III) or the secondary (MDS-UPDRS Part III) endpoint^6^. Prasinezumab and the PASADENA study have three unique features compared with cinpanemab and the SPARK study: (1) prasinezumab binds to a C-terminal epitope of α-synuclein; (2) prasinezumab targets aggregated, monomeric and intermediate oligomeric α-synuclein proteospecies^15^; and (3) the PASADENA study included both participants taking MAO-B inhibitors at baseline and participants who were treatment-naive. The results in the treatment-naive subpopulation in the PASADENA study are not dissimilar to those from the SPARK study. Cinpanemab binds to an N-terminal epitope of α-synuclein and only to aggregated α-synuclein, and unlike prasinezumab, not to monomeric or oligomeric proteospecies^6^.

In conclusion, prasinezumab showed a numerical effect on slowing motor progression in the PASADENA study, and the effect was greater in subpopulations of individuals with rapidly progressing disease. For subpopulations of individuals with slower progressing disease, a clinical trial should be longer than 1-year duration. It is important to emphasize, however, that the current report is an exploratory analysis of a phase 2 study that failed to show an effect on the primary endpoint. Thus, a phase 2 randomized trial is required to support further the hypothesis that prasinezumab can slow progression in early-stage PD. Notably, PADOVA (NCT04777331) is an ongoing phase 2b study that will test the effect of prasinezumab on slowing motor progression in early-stage PD populations on stable treatment with MAO-B inhibitors or levodopa.

## Methods

### Ethics statement

The trial was conducted according to the principles of the Declaration of Helsinki and Good Clinical Practice guidelines, and was approved by central institutional review boards (Ethikkommission der Medizinischen Universität Innsbruck (Austria), Comité de Protection des Personnes (CPP) Ouest IV (France), Ethikkommission der LÄK Hessen (Germany), CEIm Hospital Universitari Vall d’Hebron (Spain), Copernicus Group Independent Review Board (United States) and Western Institutional Review Board (United States)) or ethics committees at each trial site (Ethikkommission der Universität Leipzig Geschäftsstelle der Ethikkommission an der Medizinischen Fakultät der Universität Leipzig (Germany), Ethikkommission der Fakultät für Medizin der Technischen Universität München (Germany), Ethikkommission der Universität Ulm (Oberer Eselsberg) (Germany), Landesamt für Gesundheit und Soziales Berlin Geschäftsstelle der Ethik-Kommission des Landes Berlin (Germany), Ethikkommission des FB Medizin der Philipps-Universität Marburg (Germany), Ethikkommission an der Medizinischen Fakultät der Eberhard-Karls-Universität und am Universitätsklinikum Tübingen (Germany), Ethikkommission an der Medizinischen Fakultät der HHU Düsseldorf (Germany), The University of Kansas Medical Center Human Research Protection Program (United States), Oregon Health & Science University Independent Review Board (United States), Northwestern University Institutional Review Board (United States), Spectrum Health Human Research Protection Program (United States), The University of Vermont Committees on Human Subjects (United States), Beth Israel Deaconess Medical Center Committee on Clinical Investigations, New Procedures and New Forms of Therapy (United States), Vanderbilt Human Research Protection Program Health (United States), University of Maryland, Baltimore Institutional Review Board (United States), University of Southern California Institutional Review Board (United States), Columbia University Medical Center Institutional Review Board (United States), University of Southern California San Francisco Institutional Review Board (United States), University of Pennsylvania Institutional Review Board (United States) and HCA – HealthOne Institutional Review Board (United States)).

All participants provided written informed consent before undergoing any trial-specific screening tests or evaluations.

### Participants

Eligibility criteria for the PASADENA study have been reported previously^10^. Key inclusion criteria included: idiopathic PD with bradykinesia and one of the other cardinal signs of PD (resting tremor, rigidity) and no other known or suspected cause of PD; age 40–80 years; and a DaT-SPECT consistent with PD. Participants were also required to be either treatment-naive or on a stable dose of a MAO-B inhibitor for at least 90 days. Key exclusion criteria included: a medical history indicating a Parkinson syndrome other than idiopathic PD; known carriers of certain familial PD genes (*Parkin*, *PINK1*, *DJ1*); Mini Mental State Examination ≤25; use of catechol-*O*-methyl transferase inhibitors (entacapone, tolcapone), amantadine or anticholinergics, or dopaminergic medication (levodopa and dopamine agonists) for more than a total of 60 days or within 60 days of baseline; and previous participation in any prasinezumab study^10^.

### Study design

Full details of the study design and results are published elsewhere^4,10^. The multicenter and multinational study was powered to assess a difference of 3 points between the prasinezumab and placebo groups in the change from baseline to week 52 in the sum of scores on Parts I, II and III of the MDS-UPDRS.

#### Endpoints

The results of the subpopulation analyses of the following secondary endpoints in PASADENA Part 1 (randomized controlled part of the study) are reported: MDS-UPDRS Parts I, II and III. Following the International Council for Harmonization of Technical Requirements for Pharmaceuticals for Human Use (ICH) E9 (R1) addendum, analyses were performed using two estimand strategies to handle the post-randomization event of start or increase of symptomatic treatment: (1) ‘hypothetical strategy’, the estimated treatment effect assumes a scenario in which the events of start of symptomatic therapy or change in MAO-B inhibitor dose did not occur; and (2) ‘treatment policy’, an assessment of treatment effect irrespective of symptomatic treatment start or changes in MAO-B inhibitor treatment. The hypothetical strategy implies that the data following the first dose of symptomatic treatment or change in MAO-B inhibitor dose are excluded from the analysis; instead, the treatment effect from these participants is estimated through the covariance matrix of the mixed models for repeated measures model. For the treatment policy analysis of MDS-UPDRS Part III, all the data are included in the analysis, regardless of symptomatic treatment intake. Two scenarios are considered in this case: (1) measurements in practically defined OFF state (12 h after withdrawal of levodopa), and (2) ON state (after taking levodopa).

#### Description of subpopulations

A subpopulation analysis was performed if there were at least 20% of patients from the mITT in each of the subpopulation groups at baseline (Table 1). To derive the PIGD and tremor-dominant motor subphenotypes, the following definitions were used: tremor score was defined as the mean of the MDS-UPDRS items 2.10 tremor, 3.15a (postural tremor—right hand), 3.15b (postural tremor—left hand), 3.16a (kinetic tremor—right hand), 3.16b (kinetic tremor—left hand), 3.17a (rest tremor amplitude—right upper extremity), 3.17b (rest tremor amplitude—left upper extremity), 3.17c (rest tremor amplitude—right lower extremity), 3.17d (rest tremor amplitude—left lower extremity), 3.17e (rest tremor amplitude—lip/jaw) and 3.18 (constancy of rest tremor). PIGD score was defined as sum of an individual’s baseline falling, walking, freezing, gait and postural stability scores (3.11 and 3.12), divided by 5. The ratio of tremor score to PIGD score was calculated; a subject was defined as ‘tremor-dominant’ if the ratio was ≥1.15 OR the PIGD score was 0 and the tremor score was >0; a subject was defined as having PIGD if the ratio was ≤0.9; and a subject was defined as being ‘intermediate’ if the ratio was >0.9 and <1.15, OR if the tremor score and PIGD score were 0.

For the derivation of the akinetic–rigid motor subphenotype, the akinetic–rigid score was calculated as the average of the items of bradykinesia, rigidity and axial symptoms. The ratio of mean tremor-dominant score/mean akinetic–rigid score was then calculated. Subjects were classified as having the ‘akinetic–rigid subphenotype’ if they had a ratio <0.8, ‘tremor-dominant subphenotype’ if they had a ratio ≥1.0 and ‘intermediate’ if they had a ratio between 0.8 and 1.

To derive the data-driven subphenotypes (diffuse malignant), scales were classified into ‘motor’ and ‘nonmotor’. The motor scales were MDS-UPDRS Part II (motor symptoms) and MDS-UPDRS Part III (motor signs). The nonmotor scales were the Scale for Outcomes in PD for Autonomic symptoms (autonomic dysfunction), Rapid Eye Movement Sleep Behavior Disorder Screening Questionnaire (sleep problems) and Montreal Cognitive Assessment (cognitive impairment). The ‘diffuse malignant’ subpopulation was defined as either motor score (MDS-UPDRS Part II or MDS-UPDRS Part III) greater than the 75th percentile and at least one nonmotor score (autonomic dysfunction, sleep problems or cognitive impairment) greater than the 75th percentile or all three nonmotor scores greater than the 75th percentile. The ‘nondiffuse malignant’ subpopulation was defined as all the remaining participants not being classified as diffuse malignant.

### Randomization and blinding

In Part 1 of the PASADENA study, participants were randomized with a 1:1:1 allocation ratio to either placebo, a high dose of prasinezumab (referred to as the 4,500 mg group throughout, although participants with a body weight of <65 kg received just 3,500 mg) or a low dose of prasinezumab (1,500 mg for all body weights), as previously reported^10^. Randomization was stratified by sex, age group (<60 years versus ≥60 years) and use of an MAO-B inhibitor at baseline (yes versus no)^10^. Randomization was conducted via an Interactive Voice/Web Response System^4^. The randomization list was made available to the bioanalytical manager and to the unblinded pharmacists preparing the study drug at the sites^4^. The list was also provided to specialists external to the sponsor, as needed, to create unblinded data displays for the Independent Data Monitoring Committee (iDMC) reviews^4^. Members of the iDMC were fully unblinded; no sponsor personnel had access to the unblinded data displays reviewed by the iDMC. Treatment assignment for Part 1 was only available to the sponsor personnel after completion of Part 1 of the study.

### Sample size

As reported previously^10^, a sample size of ~100 randomized participants per group (300 participants in total) was estimated, which allowed for a power of ~80% at a two-sided ɑ-level of 20% to detect a three-point difference in MDS-UPDRS sum of Parts I + II + III between groups from baseline at week 52. The three-point difference was chosen based on the clinical judgment of expert consultants in movement disorders and modeling of PPMI data, which were used to model disease progression in the placebo arm of the PASADENA study^10^.

### Statistical analyses

All the analyses were performed in the mITT population; that is, all patients randomized in the study who received any amount of study drug treatment. The endpoints were analyzed by mixed models for repeated measures, using as covariates the stratification factors age at baseline (<60 years versus ≥60 years), sex at birth (male versus female), MAO-B inhibitor treatment at baseline (yes versus no) and the DaT-SPECT SBR in the putamen contralateral to the clinically most affected side (see also Pagano et al. ^4^). For subpopulations described by a covariate, the corresponding covariate was removed from the analysis. Primary analyses tested for differences in change from baseline between prasinezumab versus placebo, for each subpopulation separately. In this analysis, the prasinezumab 1,500 mg and 4,500 mg groups were pooled as no dose-response was previously found^4^. RR values were calculated as the ratio between the difference in estimated mean change from the baseline of the pooled prasinezumab group and the placebo group, divided by the estimated mean change from the baseline in the placebo group. Statistical analyses were obtained with SAS v.9.04 and R v.4.0.3. Data were collected using the eCRF Medidata Classic Rave 2021.1.1. The MDS-UPDRS data were collected using Virgil Investigative Study Platform provided by MedAvante, Inc. (WCG Clinical).

### Reporting summary

Further information on research design is available in the Nature Portfolio Reporting Summary linked to this article.

## Online content

Any methods, additional references, Nature Portfolio reporting summaries, source data, extended data, supplementary information, acknowledgements, peer review information; details of author contributions and competing interests; and statements of data and code availability are available at 10.1038/s41591-024-02886-y.

### Supplementary information

Supplementary Information Supplementary materials, Tables 1–7 and Fig. 1.

Reporting Summary

## Data Availability

Qualified researchers may request access to individual patient-level data through the clinical study data request platform (https://vivli.org/). Further details on Roche’s criteria for eligible studies are available at https://vivli.org/members/ourmembers/. For further details on Roche’s Global Policy on the Sharing of Clinical Information and how to request access to related clinical study documents, see: https://www.roche.com/research_and_development/who_we_are_how_we_work/clinical_trials/our_commitment_to_data_sharing.htm.
