# Supplementary material for: Prasinezumab slows motor progression in rapidly progressing early-stage Parkinson’s disease
Source: Nat Med. 2024 Apr 15;30(4):1096–103. doi: 10.1038/s41591-024-02886-y (PMC11031390; doi:10.1038/s41591-024-02886-y)
Supplement: Supplementary file 1 — Supplementary materials, Tables 1–7 and Fig. 1. [file 41591_2024_2886_MOESM1_ESM.pdf]

nature medicine

Article

https://doi.org/10.1038/s41591-024-02886-y

Prasinezumab slows motor progression in rapidly progressing early-stage Parkinson's disease

In the format provided by the authors and unedited

Supplementary Materials

Results

Supplementary Table 1 summarizes the baseline demographic and clinical characteristics of all subpopulations included in the main manuscript. Data are shown for placebo, low dose (1500 mg) prasinezumab, high dose (4500 mg) prasinezumab and pooled prasinezumab (low and high dose). Treatment responses (change from baseline at Week 52 in MDS-UPDRS Part I, II and III scores) for placebo and prasinezumab are shown in Supplementary Table 2 (pooled prasinezumab), Supplementary Table 3 (low dose prasinezumab), Supplementary Table 4 (high dose prasinezumab) and Supplementary Fig. 1. Supplementary Table 5 summarizes the use of symptomatic treatment at all visits for the placebo and pooled prasinezumab groups; data are shown for the whole population and all subpopulations. Supplementary Table 6 presents the levodopa equivalent daily dose for the MDS-UPDRS Part III OFF assessment at all visits for the placebo and pooled prasinezumab groups; data are shown for the whole population and all subpopulations. Supplementary Table 7 presents the percentage of patients in a specific fast-progressing subgroup who also belonged to other fast-progressing subgroups.

The baseline characteristics and treatment responses for placebo versus pooled prasinezumab are described in more detail below.

Supplementary Table 1: Baseline demographic and clinical characteristics of the study participants in all subpopulations

MAO-B inhibitor at baseline								
	**Treatment-naïve**				**MAO-B inhibitors**			
	**Placebo (n=67)**	**Prasinezumab pooled (n=134)**	**Prasinezumab low dose (n=67)**	**Prasinezumab high dose (n=67)**	**Placebo (n=38)**	**Prasinezumab pooled (n=77)**	**Prasinezumab low dose (n=38)**	**Prasinezumab high dose (n=39)**
Age (years), mean (SD)	60.8 (8.8)	60.9 (9.2)	61.7 (8.6)	60.0 (9.7)	58.3 (8.4)	58.2 (9.4)	57.9 (8.8)	58.5 (10.0)
Sex (male), n (%)	47 (70.1)	92 (68.7)	46 (68.7)	46 (68.7)	24 (63.2)	50 (64.9)	25 (65.8)	25 (64.1)
Time since diagnosis (months), mean (SD)	8.83 (6.81)	9.17 (6.37)	8.82 (6.11)	9.51 (6.65)	11.93 (6.37)	11.98 (5.99)	12.88 (5.73)	11.09 (6.19)
Time since diagnosis ≤12 months, n (%)	50 (74.6)	100 (74.6)	51 (76.1)	49 (73.1)	22 (57.9)	47 (61.0)	19 (50.0)	28 (71.8)
Hoehn and Yahr Stage, n (%)								
Stage I	13 (19.4)	33 (24.6)	17 (25.4)	16 (23.9)	7 (18.4)	25 (32.5)	12 (31.6)	13 (33.3)
Stage II	54 (80.6)	101 (75.4)	50 (74.6)	51 (76.1)	31 (81.6)	52 (67.5)	26 (68.4)	26 (66.7)
MDS-UPDRS Total (Sum of Parts I, II and III), mean (SD)	31.93 (13.58)	32.19 (13.06)	32.64 (13.22)	31.73 (12.99)	32.16 (12.01)	29.25 (11.90)	29.45 (13.44)	29.05 (10.36)
MDS-UPDRS Part I, mean (SD)	4.82 (3.67)	4.60 (4.25)	4.87 (4.57)	4.34 (3.92)	5.08 (3.82)	4.19 (3.15)	4.24 (3.40)	4.15 (2.92)
MDS-UPDRS Part II, mean (SD)	5.39 (4.01)	5.43 (4.21)	5.43 (4.10)	5.42 (4.35)	5.84 (4.25)	4.87 (3.69)	4.08 (3.67)	5.64 (3.59)
MDS-UPDRS Part III, mean (SD)	21.72 (9.36)	22.16 (8.98)	22.34 (9.04)	21.97 (8.99)	21.24 (8.77)	20.18 (8.87)	21.13 (9.40)	19.26 (8.34)
DaT-SPECT SBR*, mean (SD)	1.09 (0.29)	1.09 (0.35)	1.05 (0.33)	1.12 (0.36)	1.03 (0.30)	1.02 (0.31)	1.03 (0.32)	1.02 (0.30)
Estimated average change in MDS-UPDRS Part III per month prior to the study (points per month)	2.46	2.42	2.53	2.31	1.78	1.68	1.64	1.74
Hoehn and Yahr stage								

	Stage 1				Stage 2			
	**Placebo (n=20)**	**Prasinezumab pooled (n=58)**	**Prasinezumab low dose (n=29)**	**Prasinezumab high dose (n=29)**	**Placebo (n=85)**	**Prasinezumab pooled (n=153)**	**Prasinezumab low dose (n=76)**	**Prasinezumab high dose (n=77)**
Age (years), mean (SD)	55.8 (8.7)	59.4 (9.4)	60.4 (8.2)	58.4 (10.4)	60.9 (8.4)	60.1 (9.3)	60.3 (9.1)	59.8 (9.6)
Sex (male), n (%)	13 (65.0)	34 (58.6)	20 (69.0)	14 (48.3)	58 (68.2)	108 (70.6)	51 (67.1)	57 (74.0)
Time since diagnosis (months), mean (SD)	8.55 (5.90)	9.72 (6.39)	10.75 (6.66)	8.69 (6.04)	10.28 (6.97)	10.37 (6.37)	10.11 (6.14)	10.62 (6.62)
Time since diagnosis ≤12 months, n (%)	14 (70.0)	41 (70.7)	17 (58.6)	24 (82.8)	58 (68.2)	106 (69.3)	53 (69.7)	53 (68.8)
Hoehn and Yahr Stage, n (%)<br>Stage I<br>Stage II	 20 (100)<br>0 (0)	 58 (100)<br>0 (0)	 29 (100)<br>0 (0)	 29 (100)<br>0 (0)	 0 (0)<br>85 (100)	 0 (0)<br>153 (100)	 0 (0)<br>76 (100)	 0 (0)<br>77 (100)
MDS-UPDRS Total (Sum of Parts I, II and III), mean (SD)	21.45 (7.69)	22.41 (9.32)	21.28 (9.38)	23.55 (9.28)	34.49 (12.74)	34.41 (12.27)	35.38 (12.55)	33.45 (11.98)
MDS-UPDRS Part I, mean (SD)	4.70 (3.16)	4.16 (3.41)	4.07 (3.27)	4.24 (3.59)	4.96 (3.84)	4.57 (4.05)	4.86 (4.48)	4.29 (3.59)
MDS-UPDRS Part II, mean (SD)	4.40 (4.43)	4.43 (3.34)	3.59 (2.98)	5.28 (3.50)	5.82 (3.98)	5.52 (4.23)	5.46 (4.21)	5.58 (4.28)
MDS-UPDRS Part III, mean (SD)	12.35 (4.46)	13.83 (6.37)	13.62 (6.41)	14.03 (6.44)	23.71 (8.57)	24.32 (8.09)	25.07 (8.00)	23.58 (8.17)
DaT-SPECT SBR*, mean (SD)	1.19 (0.36)	1.13 (0.32)	1.08 (0.32)	1.18 (0.31)	1.04 (0.27)	1.04 (0.34)	1.03 (0.33)	1.05 (0.35)
Estimated average change in MDS-UPDRS Part III per month prior to the study (points per month)	1.44	1.42	1.27	1.61	2.31	2.35	2.48	2.22
REM sleep behavior disorder score (RBDSQ)								
	**<5**				**≥5**			
	**Placebo (n=81)**	**Prasinezumab pooled**	**Prasinezumab low dose**	**Prasinezumab high dose**	**Placebo (n=24)**	**Prasinezumab pooled**	**Prasinezumab low dose**	**Prasinezumab high dose**

		**(n=149)**	**(n=71)**	**(n=78)**		**(n=61)**	**(n=34)**	**(n=27)**
Age (years), mean (SD)	59.8 (9.0)	58.3 (9.4)	58.9 (8.7)	57.8 (10.0)	60.1 (7.8)	63.6 (8.2)	63.3 (8.6)	64.0 (7.9)
Sex (male), n (%)	54 (66.7)	102 (68.5)	50 (70.4)	52 (66.7)	17 (70.8)	39 (63.9)	21 (61.8)	18 (66.7)
Time since diagnosis (months), mean (SD)	9.78 (6.86)	10.50 (6.49)	10.82 (6.69)	10.20 (6.33)	10.56 (6.64)	9.25 (5.89)	9.18 (5.18)	9.35 (6.78)
Time since diagnosis ≤12 months, n (%)	55 (67.9)	102 (68.5)	45 (63.4)	57 (73.1)	17 (70.8)	45 (73.8)	25 (73.5)	20 (74.1)
Hoehn and Yahr Stage, n (%)								
Stage I	17 (21.0)	42 (28.2)	20 (28.2)	22 (28.2)	3 (12.5)	16 (26.2)	9 (26.5)	7 (25.9)
Stage II	64 (79.0)	107 (71.8)	51 (71.8)	56 (71.8)	21 (87.5)	45 (73.8)	25 (73.5)	20 (74.1)
MDS-UPDRS Total (Sum of Parts I, II and III), mean (SD)	30.84 (11.69)	29.16 (11.52)	29.44 (11.65)	28.91 (11.48)	35.96 (16.28)	35.52 (14.13)	35.76 (15.61)	35.22 (12.31)
MDS-UPDRS Part I, mean (SD)	4.77 (3.57)	3.66 (3.43)	3.58 (3.52)	3.73 (3.37)	5.42 (4.20)	6.39 (4.28)	6.85 (4.61)	5.81 (3.81)
MDS-UPDRS Part II, mean (SD)	5.04 (3.67)	4.49 (3.73)	4.00 (3.23)	4.94 (4.10)	7.29 (4.95)	6.95 (4.23)	6.91 (4.69)	7.00 (3.65)
MDS-UPDRS Part III, mean (SD)	21.04 (8.88)	21.01 (8.86)	21.86 (9.16)	20.24 (8.57)	23.25 (9.85)	22.18 (9.05)	22.0 (9.25)	22.41 (8.96)
DaT-SPECT SBR*, mean (SD)	1.05 (0.29)	1.10 (0.35)	1.07 (0.34)	1.12 (0.36)	1.11 (0.31)	0.98 (0.29)	0.98 (0.30)	0.99 (0.28)
Estimated average change in MDS-UPDRS Part III per month prior to the study (points per month)	2.15	2.00	2.02	1.98	2.20	2.40	2.40	2.40
Data-driven subphenotype								
	**Non-diffuse malignant**				**Diffuse malignant**			
	**Placebo (n=90)**	**Prasinezumab pooled (n=167)**	**Prasinezumab low dose (n=84)**	**Prasinezumab high dose (n=83)**	**Placebo (n=15)**	**Prasinezumab pooled (n=44)**	**Prasinezumab low dose (n=21)**	**Prasinezumab high dose (n=23)**
Age (years), mean (SD)	59.3 (8.3)	59.2 (9.5)	59.3 (8.9)	59.0 (10.0)	63.4 (10.5)	62.6 (8.4)	64.3 (7.3)	61.0 (9.2)
Sex (male), n (%)	61 (67.8)	113 (67.7)	57 (67.9)	56 (67.5)	10 (66.7)	29 (65.9)	14 (66.7)	15 (65.2)

Time since diagnosis (months), mean (SD)	9.70 (6.72)	10.60 (6.51)	10.60 (6.46)	10.59 (6.60)	11.47 (7.25)	8.66 (5.59)	9.04 (5.34)	8.31 (5.91)
Time since diagnosis ≤12 months, n (%)	62 (68.9)	109 (65.3)	53 (63.1)	56 (67.5)	10 (66.7)	38 (86.4)	17 (81.0)	21 (91.3)
Hoehn and Yahr Stage, n (%)<br>Stage I<br>Stage II	 19 (21.1)<br>71 (78.9)	 50 (29.9)<br>117 (70.1)	 26 (31.0%)<br>58 (69.0%)	 24 (28.9%)<br>59 (71.1%)	 1 (6.7)<br>14 (93.3)	 8 (18.2)<br>36 (81.8)	 3 (14.3)<br>18 (85.7)	 5 (21.7)<br>18 (78.3)
MDS-UPDRS Total (Sum of Parts I, II and III), mean (SD)	28.96 (10.43)	27.65 (10.61)	28.14 (10.88)	27.14 (10.37)	50.33 (11.81)	44.27 (11.38)	44.86 (14.05)	43.74 (8.53)
MDS-UPDRS Part I, mean (SD)	4.23 (3.14)	3.56 (3.04)	3.77 (3.27)	3.35 (2.79)	9.00 (4.31)	7.84 (4.81)	8.10 (5.55)	7.61 (4.13)
MDS-UPDRS Part II, mean (SD)	4.44 (2.99)	4.16 (3.06)	3.90 (2.93)	4.41 (3.19)	12.20 (3.47)	9.27 (4.67)	9.10 (4.93)	9.43 (4.53)
MDS-UPDRS Part III, mean (SD)	20.28 (8.47)	19.93 (8.49)	20.46 (8.55)	19.39 (8.45)	29.13 (9.43)	27.16 (8.51)	27.67 (9.37)	26.70 (7.82)
DaT-SPECT SBR*, mean (SD)	1.09 (0.29)	1.07 (0.34)	1.07 (0.35)	1.07 (0.33)	0.91 (0.29)	1.04 (0.33)	0.94 (0.22)	1.12 (0.38)
Estimated average change in MDS-UPDRS Part III per month prior to the study (points per month)	2.09	1.88	1.93	1.83	2.54	3.14	3.06	3.21
Age at baseline								
	**<60 years**				**≥60 years**			
	**Placebo (n=50)**	**Prasinezumab pooled (n=98)**	**Prasinezumab low dose (n=48)**	**Prasinezumab high dose (n=50)**	**Placebo (n=55)**	**Prasinezumab pooled (n=113)**	**Prasinezumab low dose (n=57)**	**Prasinezumab high dose (n=56)**
Age (years), mean (SD)	52.3 (5.1)	51.5 (5.8)	52.5 (5.9)	50.5 (5.5)	66.8 (4.4)	67.2 (4.5)	66.9 (4.3)	67.4 (4.6)
Sex (male), n (%)	33 (66.0)	67 (68.4)	33 (68.8)	34 (68.0)	38 (69.1)	75 (66.4)	38 (66.7)	37 (66.1)
Time since diagnosis (months), mean (SD)	9.33 (6.70)	10.68 (6.24)	11.30 (6.21)	10.08 (6.26)	10.52 (6.88)	9.77 (6.48)	9.44 (6.23)	10.11 (6.76)
Time since diagnosis ≤12 months, n (%)	38 (76.0)	62 (63.3)	27 (56.3)	35 (70.0%)	34 (61.8)	85 (75.2)	43 (75.4)	42 (75.0)

Hoehn and Yahr Stage, n (%)<br>Stage I<br>Stage II	 14 (28.0)<br>36 (72.0)	 27 (27.6)<br>71 (72.4)	 12 (25.0)<br>36 (75.0)	 15 (30.0)<br>35 (70.0)	 6 (10.9)<br>49 (89.1)	 31 (27.4)<br>82 (72.6)	 17 (29.8)<br>40 (70.2)	 14 (25.0)<br>42 (75.0)
MDS-UPDRS Total (Sum of Parts I, II and III), mean (SD)	28.94 (12.96)	30.76 (13.64)	31.19 (14.05)	30.34 (13.37)	34.80 (12.46)	31.42 (11.87)	31.74 (12.80)	31.11 (10.96)
MDS-UPDRS Part I, mean (SD)	5.08 (4.20)	4.02 (3.84)	4.27 (4.46)	3.78 (3.16)	4.76 (3.23)	4.83 (3.90)	4.95 (3.94)	4.71 (3.88)
MDS-UPDRS Part II, mean (SD)	4.88 (4.06)	5.13 (3.94)	4.58 (3.93)	5.66 (3.91)	6.16 (4.04)	5.30 (4.12)	5.25 (4.04)	5.36 (4.24)
MDS-UPDRS Part III, mean (SD)	18.98 (8.53)	21.60 (9.38)	22.33 (8.88)	20.90 (9.88)	23.87 (9.07)	21.29 (8.63)	21.54 (9.42)	21.04 (7.83)
DaT-SPECT SBR*, mean (SD)	1.16 (0.28)	1.12 (0.35)	1.12 (0.38)	1.13 (0.32)	0.98 (0.29)	1.01 (0.31)	0.97 (0.26)	1.04 (0.36)
Estimated average change in MDS-UPDRS Part III per month prior to the study (points per month)	2.03	2.02	1.98	2.07	2.27	2.18	2.28	2.08
Sex								
	**Female**				**Male**			
	**Placebo (n=34)**	**Prasinezumab pooled (n=69)**	**Prasinezumab low dose (n=34)**	**Prasinezumab high dose (n=35)**	**Placebo (n=71)**	**Prasinezumab pooled (n=142)**	**Prasinezumab low dose (n=71)**	**Prasinezumab high dose (n=71)**
Age (years), mean (SD)	59.4 (9.1)	60.5 (8.7)	60.8 (8.6)	60.2 (9.0)	60.1 (8.5)	59.6 (9.6)	60.1 (9.0)	59.0 (10.3)
Sex (male), n (%)	0 (0)	0 (0)	0 (0)	0 (0)	71 (100)	142 (100)	71 (100)	71 (100)
Time since diagnosis (months), mean (SD)	11.03 (7.13)	9.78 (6.58)	10.30 (6.60)	9.28 (6.61)	9.44 (6.61)	10.39 (6.28)	10.28 (6.14)	10.50 (6.45)
Time since diagnosis ≤12 months, n (%)	22 (64.7)	51 (73.9)	25 (73.5)	26 (74.3)	50 (70.4)	96 (67.6)	45 (63.4)	51 (71.8)
Hoehn and Yahr Stage, n (%)<br>Stage I<br>Stage II	 7 (20.6)<br>27 (79.4)	 24 (34.8)<br>45 (65.2)	 9 (26.5)<br>25 (73.5)	 15 (42.9)<br>20 (57.1)	 13 (18.3)<br>58 (81.7)	 34 (23.9)<br>108 (76.1)	 20 (28.2)<br>51 (71.8)	 14 (19.7)<br>57 (80.3)

MDS-UPDRS Total (Sum of Parts I, II and III), mean (SD)	29.35 (11.81)	28.93 (12.80)	29.12 (14.03)	28.74 (11.69)	33.28 (13.39)	32.18 (12.56)	32.62 (12.92)	31.73 (12.26)
MDS-UPDRS Part I, mean (SD)	5.06 (4.16)	4.52 (4.06)	4.62 (4.28)	4.43 (3.90)	4.85 (3.50)	4.42 (3.81)	4.65 (4.16)	4.20 (3.43)
MDS-UPDRS Part II, mean (SD)	4.91 (3.86)	4.52 (3.86)	4.38 (4.03)	4.66 (3.74)	5.86 (4.18)	5.56 (4.08)	5.21 (3.96)	5.92 (4.19)
MDS-UPDRS Part III, mean (SD)	19.38 (7.86)	19.88 (9.31)	20.12 (9.73)	19.66 (9.03)	22.58 (9.53)	22.19 (8.73)	22.76 (8.79)	21.62 (8.70)
DaT-SPECT SBR*, mean (SD)	1.09 (0.33)	1.11 (0.32)	1.07 (0.27)	1.15 (0.37)	1.05 (0.28)	1.04 (0.34)	1.03 (0.35)	1.05 (0.33)
Estimated average change in MDS-UPDRS Part III per month prior to the study (points per month)	1.76	2.03	1.95	2.12	2.39	2.14	2.21	2.06
Disease duration								
	**<12 months**				**>12 months**			
	**Placebo (n=72)**	**Prasinezumab pooled (n=147)**	**Prasinezumab low dose (n=70)**	**Prasinezumab high dose (n=77)**	**Placebo (n=33)**	**Prasinezumab pooled (n=64)**	**Prasinezumab low dose (n=35)**	**Prasinezumab high dose (n=29)**
Age (years), mean (SD)	59.4 (8.6)	60.8 (9.1)	61.5 (8.0)	60.1 (9.9)	61.0 (8.9)	57.8 (9.7)	57.9 (10.0)	57.7 (9.4)
Sex (male), n (%)	50 (69.4)	96 (65.3)	45 (64.3)	51 (66.2)	21 (63.6)	46 (71.9)	26 (74.3)	20 (69.0)
Time since diagnosis (months), mean (SD)	5.97 (2.80)	6.61 (2.98)	6.53 (2.97)	6.69 (3.01)	18.65 (4.30)	18.41 (4.02)	17.81 (3.84)	19.13 (4.18)
Time since diagnosis ≤12 months, n (%)	72 (100)	147 (100)	70 (100)	77 (100)	0 (0)	0 (0)	0 (0)	0 (0)
Hoehn and Yahr Stage, n (%)<br>Stage I<br>Stage II	 14 (19.4)<br>58 (80.6)	 41 (27.9)<br>106 (72.1)	 17 (24.3)<br>53 (75.7)	 24 (31.2)<br>53 (68.8)	 6 (18.2)<br>27 (81.8)	 17 (26.6)<br>47 (73.4)	 12 (34.3)<br>23 (65.7)	 5 (17.2)<br>24 (82.8)
MDS-UPDRS Total (Sum of Parts I, II and III), mean (SD)	31.44 (13.36)	32.40 (13.16)	32.14 (14.00)	32.64 (12.44)	33.24 (12.21)	28.16 (11.12)	30.17 (11.95)	25.72 (9.67)
MDS-UPDRS Part I, mean (SD)	4.99 (3.83)	4.92 (4.10)	5.00 (4.49)	4.84 (3.73)	4.76 (3.49)	3.39 (3.11)	3.91 (3.42)	2.76 (2.61)
MDS-UPDRS Part II, mean (SD)	5.72 (4.37)	5.49 (4.24)	5.13 (4.12)	5.82 (4.35)	5.18 (3.40)	4.61 (3.44)	4.57 (3.72)	4.66 (3.14)

MDS-UPDRS Part III, mean (SD)	20.74 (8.79)	21.99 (9.17)	22.01 (9.42)	21.97 (9.00)	23.30 (9.68)	20.16 (8.42)	21.69 (8.68)	18.31 (7.84)
DaT-SPECT SBR*, mean (SD)	1.08 (0.29)	1.07 (0.34)	1.05 (0.34)	1.10 (0.35)	1.04 (0.32)	1.03 (0.32)	1.03 (0.31)	1.04 (0.33)
Estimated average change in MDS-UPDRS Part III per month prior to the study (points per month)	3.47	3.33	3.37	3.28	1.25	1.10	1.22	0.96
Age at diagnosis								
	**<60 years**				**≥60 years**			
	**Placebo (n=52)**	**Prasinezumab pooled (n=102)**	**Prasinezumab low dose (n=50)**	**Prasinezumab high dose (n=52)**	**Placebo (n=53)**	**Prasinezumab pooled (n=109)**	**Prasinezumab low dose (n=55)**	**Prasinezumab high dose (n=54)**
Age (years), mean (SD)	52.6 (5.2)	51.9 (5.9)	52.8 (6.0)	50.9 (5.7)	67.1 (4.3)	67.4 (4.3)	67.2 (4.2)	67.6 (4.5)
Sex (male), n (%)	34 (65.4)	70 (68.6)	34 (68.0)	36 (69.2)	37 (69.8)	72 (66.1)	37 (67.3)	35 (64.8)
Time since diagnosis (months), mean (SD)	9.67 (6.79)	10.88 (6.28)	11.33 (6.16)	10.45 (6.42)	10.24 (6.84)	9.54 (6.41)	9.34 (6.26)	9.75 (6.61)
Time since diagnosis ≤12 months, n (%)	38 (73.1)	63 (61.8)	28 (56.0)	35 (67.3)	34 (64.2)	84 (77.1)	42 (76.4)	42 (77.8)
Hoehn and Yahr Stage, n (%)								
Stage I	14 (26.9)	27 (26.5)	12 (24.0)	15 (28.8)	6 (11.3)	31 (28.4)	17 (30.9)	14 (25.9)
Stage II	38 (73.1)	75 (73.5)	38 (76.0)	37 (71.2)	47 (88.7)	78 (71.6)	38 (69.1)	40 (74.1)
MDS-UPDRS Total (Sum of Parts I, II and III), mean (SD)	28.63 (12.80)	30.50 (13.45)	30.80 (13.89)	30.21 (13.13)	35.32 (12.39)	31.69 (12.00)	32.11 (12.88)	31.26 (11.13)
MDS-UPDRS Part I, mean (SD)	4.96 (4.18)	3.93 (3.79)	4.16 (4.40)	3.71 (3.12)	4.87 (3.22)	4.94 (3.92)	5.07 (3.96)	4.81 (3.91)
MDS-UPDRS Part II, mean (SD)	4.77 (4.02)	5.05 (3.92)	4.48 (3.91)	5.60 (3.89)	6.32 (4.04)	5.39 (4.14)	5.36 (4.04)	5.41 (4.27)
MDS-UPDRS Part III, mean (SD)	18.90 (8.37)	21.52 (9.25)	22.16 (8.75)	20.90 (9.76)	24.13 (9.14)	21.36 (8.74)	21.67 (9.56)	21.04 (7.89)
DaT-SPECT SBR*, mean (SD)	1.17 (0.28)	1.12 (0.35)	1.11 (0.37)	1.12 (0.33)	0.96 (0.28)	1.01 (0.31)	0.98 (0.27)	1.05 (0.36)
Estimated average change in MDS-UPDRS Part III per month	1.95	1.98	1.96	2.00	2.36	2.24	2.32	2.16

prior to the study (points per month)								
Motor subphenotypes akinetic-rigid and tremor dominant								
	**Tremor dominant**				**Akinetic-rigid**			
	**Placebo (n=41)**	**Prasinezumab pooled (n=81)**	**Prasinezumab low dose (n=38)**	**Prasinezumab high dose (n=43)**	**Placebo (n=52)**	**Prasinezumab pooled (n=113)**	**Prasinezumab low dose (n=57)**	**Prasinezumab high dose (n=56)**
Age (years), mean (SD)	59.4 (7.7)	59.6 (8.7)	59.6 (9.2)	59.6 (8.4)	59.0 (9.1)	59.6 (9.8)	60.3 (8.5)	58.8 (10.9)
Sex (male), n (%)	28 (68.3)	54 (66.7)	26 (68.4)	28 (65.1)	37 (71.2)	77 (68.1)	38 (66.7)	39 (69.6)
Time since diagnosis (months), mean (SD)	10.15 (6.93)	11.37 (6.64)	10.92 (6.60)	11.76 (6.72)	9.06 (6.23)	9.26 (5.97)	9.84 (6.13)	8.66 (5.79)
Time since diagnosis ≤12 months, n (%)	29 (70.7)	51 (63.0)	22 (57.9)	29 (67.4)	37 (71.2)	85 (75.2)	42 (73.7)	43 (76.8)
Hoehn and Yahr Stage, n (%)								
Stage I	10 (24.4)	31 (38.3)	16 (42.1)	15 (34.9)	9 (17.3)	23 (20.4)	11 (19.3)	12 (21.4)
Stage II	31 (75.6)	50 (61.7)	22 (57.9)	28 (65.1)	43 (82.7)	90 (79.6)	46 (80.7)	44 (78.6)
MDS-UPDRS Total (Sum of Parts I, II and III), mean (SD)	30.10 (11.76)	30.36 (13.02)	29.74 (12.87)	30.91 (13.29)	32.08 (13.56)	31.42 (12.07)	32.86 (13.27)	29.96 (10.62)
MDS-UPDRS Part I, mean (SD)	4.44 (3.84)	4.46 (3.83)	4.92 (4.49)	4.05 (3.13)	5.10 (3.36)	4.60 (4.03)	4.68 (4.10)	4.52 (4.00)
MDS-UPDRS Part II, mean (SD)	4.71 (3.68)	5.40 (4.33)	5.16 (4.21)	5.60 (4.47)	6.06 (4.38)	5.19 (3.89)	4.98 (4.01)	5.39 (3.79)
MDS-UPDRS Part III, mean (SD)	20.95 (8.64)	20.51 (8.46)	19.66 (7.93)	21.26 (8.92)	20.92 (9.48)	21.64 (8.83)	23.19 (9.14)	20.05 (8.30)
DaT-SPECT SBR*, mean (SD)	1.11 (0.30)	1.09 (0.33)	1.08 (0.30)	1.10 (0.36)	1.05 (0.30)	1.03 (0.34)	1.00 (0.35)	1.05 (0.34)
Estimated average change in MDS-UPDRS Part III per month prior to the study (points per month)	2.06	1.80	1.80	1.81	2.31	2.34	2.36	2.32
Motor subphenotypes PIGD and tremor dominant								
	**Tremor dominant**				**PIGD**			

	Placebo (n=77)	Prasinezumab pooled (n=144)	Prasinezumab low dose (n=72)	Prasinezumab high dose (n=72)	Placebo (n=23)	Prasinezumab pooled (n=47)	Prasinezumab low dose (n=24)	Prasinezumab high dose (n=23)
Age (years), mean (SD)	60.1 (8.3)	60.0 (9.2)	60.0 (9.0)	60.1 (9.5)	58.2 (9.7)	58.9 (9.5)	61.1 (7.7)	56.7 (10.7)
Sex (male), n (%)	50 (64.9)	97 (67.4)	49 (68.1)	48 (66.7)	18 (78.3)	31 (66.0)	15 (62.5)	16 (69.6)
Time since diagnosis (months), mean (SD)	10.79 (7.14)	10.50 (6.34)	10.79 (6.23)	10.21 (6.47)	7.35 (5.48)	10.11 (6.44)	9.72 (6.17)	10.51 (6.82)
Time since diagnosis ≤12 months, n (%)	50 (64.9)	99 (68.8)	45 (62.5)	54 (75.0)	19 (82.6)	30 (63.8)	17 (70.8)	13 (56.5)
Hoehn and Yahr Stage, n (%)								
Stage I	12 (15.6)	42 (29.2)	23 (31.9)	19 (26.4)	7 (30.4)	12 (25.5)	5 (20.8)	7 (30.4)
Stage II	65 (84.4)	102 (70.8)	49 (68.1)	53 (73.6)	16 (69.6)	35 (74.5)	19 (79.2)	16 (69.6)
MDS-UPDRS Total (Sum of Parts I, II and III), mean (SD)	31.40 (11.90)	31.53 (12.92)	31.39 (13.26)	31.68 (12.65)	33.43 (16.45)	28.85 (12.23)	30.58 (13.25)	27.04 (11.08)
MDS-UPDRS Part I, mean (SD)	4.64 (3.65)	4.33 (3.80)	4.35 (3.96)	4.31 (3.66)	5.52 (3.85)	4.70 (3.98)	5.13 (4.46)	4.26 (3.45)
MDS-UPDRS Part II, mean (SD)	4.84 (3.48)	5.09 (3.89)	4.67 (3.58)	5.51 (4.16)	7.96 (5.36)	5.17 (4.31)	4.79 (4.36)	5.57 (4.32)
MDS-UPDRS Part III, mean (SD)	21.92 (8.75)	22.12 (9.20)	22.38 (9.62)	21.86 (8.83)	19.96 (9.80)	18.98 (8.40)	20.67 (8.11)	17.22 (8.51)
DaT-SPECT SBR*, mean (SD)	1.06 (0.31)	1.06 (0.33)	1.03 (0.29)	1.10 (0.36)	1.05 (0.26)	1.03 (0.35)	1.04 (0.41)	1.02 (0.28)
Estimated average change in MDS-UPDRS Part III per month prior to the study (points per month)	2.03	2.11	2.07	2.14	2.72	1.88	2.13	1.64

n represents number of participants contributing to summary statistics. Percentages are based on n.

*Putamen-ipsilateral.

DaT-SPECT, dopamine transporter-single-photon emission computed tomography; MAO-B, monoamine oxidase-B; MDS-UPDRS, Movement Disorder Society-Unified Parkinson’s Disease Rating Scale; PIGD, postural instability gait dysfunction; RBDSQ, Rapid Eye Movement Sleep Behavior Disorder Screening Questionnaire; REM, rapid eye movement; SD, standard deviation, SBR, striatal binding ratio.

Supplementary Table 2: Change from baseline at Week 52 for placebo and prasinezumab pooled dose in all subpopulations except those taking MAO-B inhibitors at baseline and those who were treatment-naïve at baseline

Hoehn and Yahr stage								
	**Placebo**		**Prasinezumab pooled**					
	**Stage 1 (n=20)**	**Stage 2 (n=85)**	**Stage 1 (n=58)**			**Stage 2 (n=153)**		
	**Adjusted mean (SE)**	**Adjusted mean (SE)**	**Difference in adjusted means (SE)**	**80% CI**	**%RR**	**Difference in adjusted means (SE)**	**80% CI**	**%RR**
MDS-UPDRS Part III								
Hypothetical strategy*	2.17 (1.836) n=17	6.34 (1.038) n=59	3.14 (2.173) n=41	0.32, 5.95	+144.7	–2.55 (1.276) n=106	–4.19, –0.90	–40.2
Treatment policy OFF#	1.60 (1.779) n=19	3.79 (0.908) n=82	3.15 (2.055) n=56	0.49, 5.81	+196.9	–1.33 (1.119) n=145	–2.76, 0.11	–35.1
Treatment policy ON#	1.18 (1.863) n=20	2.79 (0.952) n=85	3.16 (2.145) n=57	0.39, 5.93	+267.8	–1.51 (1.178) n=149	–3.02, 0.00	–54.1
MDS-UPDRS Part II								
Hypothetical strategy*	1.68 (0.703) n=17	3.01 (0.434) n=59	0.80 (0.815) n=41	–0.26, 1.85	+47.6	0.01 (0.533) n=106	–0.68, 0.70	+0.3
Treatment policy#	0.97 (0.671) n=20	1.62 (0.413) n=85	0.67 (0.768) n=57	–0.33, 1.66	+99.0	0.16 (0.508) n=148	–0.49, 0.82	+9.9
MDS-UPDRS Part I								
Hypothetical strategy*	–0.18 (0.645) n=17	1.05 (0.334) n=59	1.01 (0.745) n=41	0.04, 1.97	–561.1	-0.40 (0.408) n=106	–0.93, 0.12	–38.1

Treatment policy[#]	–0.36 (0.643)<br>n=19	0.36 (0.326)<br>n=85	1.11 (0.736)<br>n=56	0.16, 2.07	–308.3	0.20 (0.400)<br>n=145	–0.31, 0.72	+55.6
REM sleep behavior disorder score (RBDSQ)								
	**Placebo**		**Prasinezumab pooled**					
	**<5<br>(n=81)**	**≥5<br>(n=24)**	**<5<br>(n=149)**			**≥5<br>(n=61)**		
	**Adjusted mean (SE)**	**Adjusted mean (SE)**	**Difference in adjusted means (SE)**	**80% CI**	**%RR**	**Difference in adjusted means (SE)**	**80% CI**	**%RR**
MDS-UPDRS Part III								
Hypothetical strategy*	4.98 (1.008)<br>n=57	7.76 (2.008)<br>n=19	–1.03 (1.244)<br>n=98	–2.63, 0.57	–20.7	–2.76 (2.327)<br>n=49	–5.78, 0.25	–35.6
Treatment policy OFF[#]	3.00 (0.885)<br>n=78	5.44 (1.809)<br>n=23	–0.59 (1.086)<br>n=143	–1.98, 0.81	–19.7	–1.21 (2.095)<br>n=58	–3.92, 1.49	–22.2
Treatment policy ON[#]	2.38 (0.935)<br>n=81	3.64 (1.839)<br>n=24	–1.12 (1.152)<br>n=146	–2.60, 0.36	–47.1	–0.04 (2.133)<br>n=60	–2.80, 2.71	–1.1
MDS-UPDRS Part II								
Hypothetical strategy*	2.82 (0.417)<br>n=57	2.19 (0.857)<br>n=19	0.02 (0.519)<br>n=98	–0.65, 0.69	+0.7	1.07 (0.983)<br>n=49	–0.20, 2.35	+48.9
Treatment policy[#]	1.51 (0.379)<br>n=81	1.21 (0.863)<br>n=24	0.03 (0.467)<br>n=145	–0.56, 0.63	+2.0	0.96 (1.001)<br>n=60	–0.33, 2.26	+79.3
MDS-UPDRS Part I								
Hypothetical strategy*	0.69 (0.336)<br>n=57	1.28 (0.604)<br>n=19	–0.03 (0.416)<br>n=98	–0.56, 0.51	–4.4	–0.40 (0.691)<br>n=49	–1.30, 0.49	–31.3
Treatment policy[#]	0.06 (0.326)<br>n=80	0.87 (0.634)<br>n=24	0.43 (0.403)<br>n=141	–0.09, 0.94	+716.7	0.05 (0.738)<br>n=60	–0.91, 1.00	+5.8

Data-driven subphenotype								
	**Placebo**		**Prasinezumab pooled**					
	**Non-diffuse malignant (n=90)**	**Diffuse malignant (n=15)**	**Non-diffuse malignant (n=167)**			**Diffuse malignant (n=44)**		
	**Adjusted mean (SE)**	**Adjusted mean (SE)**	**Difference in adjusted means (SE)**	**80% CI**	**%RR**	**Difference in adjusted means (SE)**	**80% CI**	**%RR**
MDS-UPDRS Part III								
Hypothetical strategy*	4.76 (0.910) n=68	12.29 (3.450) n=8	–0.77 (1.113) n=122	–2.20, 0.66	–16.2	–7.86 (3.851) n=25	–12.90, –2.82	–64.0
Treatment policy OFF#	3.29 (0.829) n=86	5.17 (2.401) n=15	–0.44 (1.006) n=161	–1.73, 0.85	–13.4	–2.58 (2.778) n=40	–6.18, 1.02	–49.9
Treatment policy ON#	2.51 (0.868) n=89	3.81 (2.598) n=16	–0.50 (1.058) n=163	–1.86, 0.86	–19.9	–2.76 (3.015) n=43	–6.67, 1.15	–72.4
MDS-UPDRS Part II								
Hypothetical strategy*	2.47 (0.373) n=68	4.08 (1.533) n=8	0.22 (0.458) n=122	–0.37, 0.81	+8.9	–0.47 (1.700) n=25	–2.69, 1.75	–11.5
Treatment policy#	1.48 (0.347) n=90	1.57 (1.334) n=15	0.20 (0.425) n=163	–0.35, 0.74	+13.5	0.06 (1.541) n=42	–1.94, 2.06	+3.8
MDS-UPDRS Part I								
Hypothetical strategy*	0.76 (0.288) n=68	1.59 (1.125) n=8	–0.20 (0.350) n=122	–0.65, 0.25	–26.3	–0.46 (1.266) n=25	–2.12, 1.20	–28.9
Treatment policy#	0.35 (0.274) n=89	0.10 (1.023) n=15	0.16 (0.334) n=160	–0.27, 0.59	+45.7	0.90 (1.165) n=41	–0.62, 2.41	+900.0
Age at baseline								

	Placebo		Prasinezumab pooled					
	<60 years (n=50)	≥60 years (n=55)	<60 years (n=98)			≥60 years (n=113)		
	Adjusted mean (SE)	Adjusted mean (SE)	Difference in adjusted means (SE)	80% CI	%RR	Difference in adjusted means (SE)	80% CI	%RR
MDS-UPDRS Part III								
Hypothetical strategy*	3.83 (1.246) n=36	7.04 (1.304) n=40	–0.61 (1.529) n=64	–2.58, 1.36	–15.9	–1.89 (1.562) n=83	–3.90, 0.12	–26.9
Treatment policy OFF#	2.27 (1.131) n=49	4.74 (1.162) n=52	–0.10 (1.372) n=95	–1.86, 1.67	–4.4	–1.02 (1.398) n=106	–2.82, 0.78	–21.5
Treatment policy ON#	1.52 (1.211) n=50	3.79 (1.204) n=55	–0.30 (1.479) n=96	–2.21, 1.60	–19.7	–1.06 (1.450) n=110	–2.93, 0.80	–28.0
MDS-UPDRS Part II								
Hypothetical strategy*	2.31 (0.531) n=36	3.04 (0.521) n=40	0.18 (0.647) n=64	–0.66, 1.01	+7.8	0.15 (0.631) n=83	–0.67, 0.96	+4.9
Treatment policy#	1.20 (0.490) n=50	1.65 (0.504) n=55	0.61 (0.595) n=95	–0.15, 1.38	+50.8	-0.07 (0.609) n=110	–0.86, 0.71	–4.2
MDS-UPDRS Part I								
Hypothetical strategy*	0.30 (0.385) n=36	1.21 (0.442) n=40	0.17 (0.470) n=64	–0.43, 0.78	+56.7	–0.20 (0.524) n=83	–0.87, 0.48	–16.5
Treatment policy#	–0.26 (0.432) n=49	0.54 (0.392) n=55	0.83 (0.525) n=93	0.15, 1.50	–319.2	0.20 (0.466) n=108	–0.40, 0.80	+37.0
Sex								
	Placebo		Prasinezumab pooled					

	Female (n=34)	Male (n=71)	Female (n=69)			Male (n=142)		
	Adjusted mean (SE)	Adjusted mean (SE)	Difference in adjusted means (SE)	80% CI	%RR	Difference in adjusted means (SE)	80% CI	%RR
MDS-UPDRS Part III								
Hypothetical strategy*	4.20 (1.647) n=26	6.18 (1.066) n=50	–1.22 (2.019) n=50	–3.83, 1.39	–29.1	–1.23 (1.292) n=97	–2.89, 0.44	–19.9
Treatment policy OFF[#]	3.95 (1.528) n=33	3.49 (0.934) n=68	–1.41 (1.853) n=67	–3.80, 0.98	–35.7	–0.18 (1.135) n=134	–1.64, 1.28	–5.2
Treatment policy ON[#]	3.14 (1.521) n=34	2.57 (1.012) n=71	–0.96 (1.853) n=68	–3.35, 1.43	–30.6	–0.60 (1.232) n=138	–2.18, 0.99	–23.4
MDS-UPDRS Part II								
Hypothetical strategy*	1.26 (0.555) n=26	3.69 (0.492) n=50	1.08 (0.682) n=50	0.20, 1.96	+85.7	–0.45 (0.598) n=97	–1.22, 0.32	–12.2
Treatment policy[#]	0.43 (0.508) n=34	2.03 (0.454) n=71	1.27 (0.618) n=68	0.47, 2.06	+295.4	–0.27 (0.554) n=137	–0.98, 0.45	–13.3
MDS-UPDRS Part I								
Hypothetical strategy*	–0.41 (0.500) n=26	1.23 (0.351) n=50	1.65 (0.620) n=50	0.85, 2.45	–402.4	–0.80 (0.426) n=97	–1.35, –0.25	–65.0
Treatment policy[#]	–0.40 (0.599) n=34	0.37 (0.296) n=70	1.81 (0.737) n=67	0.86, 2.76	–452.5	–0.24 (0.361) n=134	–0.71, 0.22	–64.9
Disease duration								
	**Placebo**		**Prasinezumab pooled**					
	**<12 months (n=72)**	**>12 months (n=33)**	**<12 months (n=147)**			**>12 months (n=64)**		

	Adjusted mean (SE)	Adjusted mean (SE)	Difference in adjusted means (SE)	80% CI	%RR	Difference in adjusted means (SE)	80% CI	%RR
MDS-UPDRS Part III								
Hypothetical strategy*	5.60 (1.084) n=55	5.98 (1.748) n=21	–1.78 (1.302) n=105	–3.45, –0.10	–31.8	–1.12 (2.160) n=42	–3.91, 1.68	–18.7
Treatment policy OFF#	3.49 (1.002) n=68	4.18 (1.383) n=33	–1.09 (1.194) n=139	–2.63, 0.44	–31.2	–0.06 (1.712) n=62	–2.27, 2.15	–1.4
Treatment policy ON#	2.59 (1.037) n=72	3.14 (1.506) n=33	–1.06 (1.241) n=144	–2.66, 0.54	–40.9	–0.24 (1.859) n=62	–2.63, 2.16	–7.6
MDS-UPDRS Part II								
Hypothetical strategy*	2.60 (0.477) n=55	3.27 (0.632) n=21	0.49 (0.574) n=105	–0.25, 1.23	+18.9	–0.80 (0.781) n=42	–1.81, 0.21	–24.5
Treatment policy#	1.33 (0.455) n=72	1.79 (0.545) n=33	0.45 (0.546) n=143	–0.25, 1.15	+33.8	–0.17 (0.671) n=62	–1.03, 0.70	–9.5
MDS-UPDRS Part I								
Hypothetical strategy*	0.42 (0.364) n=55	1.81 (0.518) n=21	0.58 (0.433) n=105	0.02, 1.14	+138.1	–1.72 (0.636) n=42	–2.55, –0.90	–95.0
Treatment policy#	0.05 (0.372) n=72	0.76 (0.440) n=32	0.79 (0.444) n=140	0.22, 1.36	+1580.0	–0.56 (0.541) n=61	–1.26, 0.14	–73.7
Age at diagnosis								
	**Placebo**		**Prasinezumab pooled**					
	**<60 years (n=52)**	**≥60 years (n=53)**	**<60 years (n=102)**			**≥60 years (n=109)**		
	**Adjusted mean (SE)**	**Adjusted mean (SE)**	**Difference in adjusted**	**80% CI**	**%RR**	**Difference in adjusted**	**80% CI**	**%RR**

			means (SE)			means (SE)		
MDS-UPDRS Part III								
Hypothetical strategy*	3.74 (1.218) n=37	7.18 (1.331) n=39	–0.52 (1.494) n=66	–2.44, 1.41	–13.9	–2.03 (1.591) n=81	–4.08, 0.02	–28.3
Treatment policy OFF[#]	2.14 (1.104) n=51	4.98 (1.191) n=50	0.15 (1.342) n=99	–1.58, 1.88	+7.0	–1.29 (1.428) n=102	–3.13, 0.54	–25.9
Treatment policy ON[#]	1.40 (1.181) n=52	3.97 (1.229) n=53	–0.21 (1.444) n=100	–2.07, 1.65	–15.0	–1.15 (1.477) n=106	–3.05, 0.75	–29.0
MDS-UPDRS Part II								
Hypothetical strategy*	2.16 (0.515) n=37	3.20 (0.536) n=39	0.30 (0.628) n=66	–0.51, 1.11	+13.9	0.04 (0.646) n=81	–0.80, 0.87	+1.3
Treatment policy[#]	1.11 (0.476) n=52	1.75 (0.521) n=53	0.67 (0.579) n=99	–0.07, 1.42	+60.4	–0.15 (0.627) n=106	–0.95, 0.66	–8.6
MDS-UPDRS Part I								
Hypothetical strategy*	0.36 (0.373) n=37	1.15 (0.455) n=39	0.12 (0.456) n=66	–0.47, 0.71	+33.3	–0.11 (0.536) n=81	–0.80, 0.58	–9.6
Treatment policy[#]	–0.18 (0.416) n=51	0.47 (0.406) n=53	0.73 (0.507) n=97	0.08, 1.39	–405.6	0.28 (0.480) n=104	–0.34, 0.90	+59.6
Motor subphenotypes akinetic rigid and tremor-dominant								
	**Placebo**		**Prasinezumab pooled**					
	**Tremor dominant (n=41)**	**Akinetic-rigid (n=52)**	**Tremor dominant (n=81)**			**Akinetic-rigid (n=113)**		
	**Adjusted mean (SE)**	**Adjusted mean (SE)**	**Difference in adjusted means (SE)**	**80% CI**	**%RR**	**Difference in adjusted means (SE)**	**80% CI**	**%RR**

MDS-UPDRS Part III								
Hypothetical strategy*	4.93 (1.386) n=26	6.00 (1.206) n=40	–0.05 (1.661) n=59	–2.19, 2.10	–1.0	–2.61 (1.451) n=77	–4.48, –0.74	–43.5
Treatment policy OFF#	3.18 (1.235) n=40	4.17 (1.076) n=50	1.29 (1.492) n=77	–0.64, 3.21	+40.6	–2.23 (1.274) n=109	–3.87, –0.59	–53.5
Treatment policy ON#	1.48 (1.337) n=41	3.84 (1.125) n=52	1.97 (1.630) n=79	–0.13, 4.07	+133.1	–2.85 (1.338) n=111	–4.57, –1.13	–74.2
MDS-UPDRS Part II								
Hypothetical strategy*	3.49 (0.580) n=26	1.83 (0.507) n=40	–1.50 (0.686) n=59	–2.39, –0.61	–43.0	1.72 (0.615) n=77	0.93, 2.52	+94.0
Treatment policy#	1.49 (0.522) n=41	1.32 (0.528) n=52	0.01 (0.630) n=78	–0.80, 0.82	+0.7	0.58 (0.635) n=111	–0.24, 1.40	+43.9
MDS-UPDRS Part I								
Hypothetical strategy*	0.87 (0.459) n=26	0.30 (0.429) n=40	–0.33 (0.539) n=59	–1.03, 0.37	–37.9	0.63 (0.516) n=77	–0.04, 1.29	+210.0
Treatment policy#	0.43 (0.476) n=41	–0.22 (0.396) n=51	0.27 (0.576) n=78	–0.47, 1.02	+62.8	0.72 (0.469) n=107	0.11, 1.32	–327.3
Motor subphenotypes PIGD and tremor dominant								
	**Placebo**		**Prasinezumab pooled**					
	**Tremor dominant (n=77)**	**PIGD (n=23)**	**Tremor dominant (n=144)**			**PIGD (n=47)**		
	**Adjusted mean (SE)**	**Adjusted mean (SE)**	**Difference in adjusted means (SE)**	**80% CI**	**%RR**	**Difference in adjusted means (SE)**	**80% CI**	**%RR**
MDS-UPDRS Part III								

Hypothetical strategy*	4.70 (1.108) n=53	8.40 (1.588) n=19	–0.44 (1.349) n=103	–2.17, 1.30	–9.4	–4.96 (1.902) n=31	–7.44, –2.48	–59.1
Treatment policy OFF#	2.88 (0.974) n=74	5.88 (1.425) n=22	0.47 (1.189) n=138	–1.06, 2.00	+16.3	–3.88 (1.681) n=44	–6.06, –1.70	–66.0
Treatment policy ON#	1.80 (1.007) n=77	5.43 (1.570) n=23	0.73 (1.236) n=141	–0.86, 2.32	+40.6	–4.86 (1.860) n=46	–7.27, –2.45	–89.5
MDS-UPDRS Part II								
Hypothetical strategy*	3.23 (0.453) n=53	1.31 (0.698) n=19	–0.79 (0.551) n=103	–1.50, –0.08	–24.5	2.14 (0.862) n=31	1.02, 3.26	+163.4
Treatment policy#	1.74 (0.400) n=77	0.83 (0.755) n=23	–0.17 (0.490) n=140	–0.80, 0.46	–9.8	0.55 (0.927) n=46	–0.65, 1.75	+66.3
MDS-UPDRS Part I								
Hypothetical strategy*	0.94 (0.346) n=53	0.09 (0.690) n=19	–0.34 (0.418) n=103	–0.88, 0.20	–36.2	1.08 (0.829) n=31	0.00, 2.16	+1200.0
Treatment policy#	0.40 (0.345) n=77	–0.54 (0.646) n=22	0.20 (0.422) n=138	–0.34, 0.74	+50.0	1.07 (0.758) n=45	0.09, 2.06	–198.2

* ‘Hypothetical strategy’ assumes a scenario in which the events of start of symptomatic therapy or change in MAO-B inhibitor dose did not occur (performed for the mITT population)

‘Treatment policy strategy’ in which the treatment effect is estimated irrespective of symptomatic treatment start or changes in MAO-B inhibitor treatment (performed for the ITT population)

CI, confidence interval; MAO-B, monoamine oxidase-B; MDS-UPDRS, Movement Disorder Society-Unified Parkinson’s Disease Rating Scale; ITT, intention-to-treat; mITT, modified intention-to-treat; PIGD, postural instability gait dysfunction; RBDSQ, Rapid Eye Movement Sleep Behavior Disorder Screening Questionnaire; REM, rapid eye movement; %RR, percentage relative reduction; SE, standard error.

Supplementary Table 3: Change from baseline at Week 52 for placebo and prasinezumab low dose in all subpopulations

MOA-B inhibitor at baseline								
	**Placebo**		**Prasinezumab low dose**					
	**Treatment-naïve (n=67)**	**MAO-B (n=38)**	**Treatment-naïve (n=67)**			**MAO-B (n=38)**		
	**Adjusted mean (SE)**	**Adjusted mean (SE)**	**Difference in adjusted means (SE)**	**80% CI**	**%RR**	**Difference in adjusted means (SE)**	**80% CI**	**%RR**
MDS-UPDRS Part III								
Hypothetical strategy*	5.04 (1.163) n=48	6.82 (1.371) n=28	-0.41 (1.636) n=45	(-2.51, 1.70)	-8.1	-4.85 (1.919) n=29	(-7.33, -2.37)	-71.1
Treatment policy OFF#	3.10 (1.048) n=65	4.79 (1.214) n=36	0.70 (1.470) n=63	(-1.19, 2.59)	+22.6	-3.77 (1.699) n=37	(-5.96, -1.58)	-78.7
Treatment policy ON#	2.01 (1.109) n=67	4.18 (1.248) n=38	0.28 (1.554) n=65	(-1.72, 2.28)	+13.9	-3.99 (1.767) n=37	(-6.27, -1.71)	-95.5
MDS-UPDRS Part II								
Hypothetical strategy*	2.89 (0.467) n=48	2.40 (0.635) n=28	0.20 (0.655) n=45	(-0.65, 1.04)	+6.9	0.47 (0.906) n=29	(-0.70, 1.65)	+19.6
Treatment policy#	1.63 (0.438) n=67	1.21 (0.592) n=38	0.26 (0.615) n=65	(-0.53, 1.05)	+16.0	0.51 (0.848) n=37	(-0.58, 1.61)	+42.1
MDS-UPDRS Part I								
Hypothetical	0.38 (0.371)	1.28 (0.466)	0.27 (0.520)	(-0.40, 0.94)	+71.1	-0.80 (0.651)	(-1.64, 0.05)	-62.5

strategy*	n=48	n=28	n=45			n=29		
Treatment policy#	0.10 (0.375)<br>n=67	0.50 (0.452)<br>n=37	0.62 (0.527)<br>n=62	(-0.06, 1.29)	+620.0	-0.21 (0.636)<br>n=37	(-1.03, 0.61)	-42.0
Hoehn and Yahr stage								
	**Placebo**		**Prasinezumab low dose**					
	**Stage 1 (n=20)**	**Stage 2 (n=85)**	**Stage 1 (n=29)**			**Stage 2 (n=76)**		
	**Adjusted mean (SE)**	**Adjusted mean (SE)**	**Difference in adjusted means (SE)**	**80% CI**	**%RR**	**Difference in adjusted means (SE)**	**80% CI**	**%RR**
MDS-UPDRS Part III								
Hypothetical strategy*	2.17 (1.836)<br>n=17	6.34 (1.038)<br>n=59	5.04 (2.380)<br>n=22	(1.96, 8.13)	+232.3	-3.87 (1.494)<br>n=52	(-5.79, -1.95)	-61.0
Treatment policy OFF#	1.60 (1.779)<br>n=19	3.79 (0.908)<br>n=82	4.77 (2.250)<br>n=29	(1.86, 7.68)	+298.1	-2.44 (1.312)<br>n=71	(-4.13, -0.76)	-64.4
Treatment policy ON#	1.18 (1.863)<br>n=20	2.79 (0.952)<br>n=85	4.57 (2.379)<br>n=29	(1.49, 7.65)	+387.3	-2.84 (1.377)<br>n=73	(-4.61, -1.07)	-101.8
MDS-UPDRS Part II								
Hypothetical strategy*	1.68 (0.703)<br>n=17	3.01 (0.434)<br>n=59	1.11 (0.916)<br>n=22	(-0.08, 2.30)	+66.1	0.20 (0.623)<br>n=52	(-0.60, 1.00)	+6.6
Treatment policy#	0.97 (0.671)<br>n=20	1.62 (0.413)<br>n=85	0.96 (0.867)<br>n=29	(-0.16, 2.09)	+99.0	0.26 (0.596)<br>n=73	(-0.51, 1.03)	+16.0
MDS-UPDRS Part I								
Hypothetical strategy*	–0.18 (0.645)<br>n=17	1.05 (0.334)<br>n=59	0.61 (0.833)<br>n=22	(-0.47, 1.70)	-338.9	-0.38 (0.477)<br>n=52	(-0.99, 0.24)	-36.2

Treatment policy#	–0.36 (0.643) n=19	0.36 (0.326) n=85	0.75 (0.816) n=29	(-0.30, 1.81)	-208.3	0.27 (0.471) n=70	(-0.34, 0.87)	+75.0
REM sleep behavior disorder score (RBDSQ)								
	**Placebo**		**Prasinezumab low dose**					
	**<5 (n=81)**	**≥5 (n=24)**	**<5 (n=71)**			**≥5 (n=61)**		
	**Adjusted mean (SE)**	**Adjusted mean (SE)**	**Difference in adjusted means (SE)**	**80% CI**	**%RR**	**Difference in adjusted means (SE)**	**80% CI**	**%RR**
MDS-UPDRS Part III								
Hypothetical strategy*	4.98 (1.008) n=57	7.76 (2.008) n=19	-1.64 (1.466) n=48	(-3.52, 0.25)	-32.9	-3.01 (2.597) n=26	(-6.37, 0.36)	-38.8
Treatment policy OFF#	3.00 (0.885) n=78	5.44 (1.809) n=23	-1.27 (1.281) n=68	(-2.91, 0.38)	-42.3	-0.99 (2.335) n=32	(-4.01, 2.02)	-18.2
Treatment policy ON#	2.38 (0.935) n=81	3.64 (1.839) n=24	-1.90 (1.360) n=69	(-3.64, -0.15)	-79.8	-0.37 (2.377) n=33	(-3.44, 2.71)	-10.2
MDS-UPDRS Part II								
Hypothetical strategy*	2.82 (0.417) n=57	2.19 (0.857) n=19	0.25 (0.609) n=48	(-0.53, 1.04)	+8.9	1.26 (1.105) n=26	(-0.17, 2.69)	+57.5
Treatment policy#	1.51 (0.379) n=81	1.21 (0.863) n=24	0.16 (0.554) n=69	(-0.55, 0.87)	+10.6	1.09 (1.116) n=33	(-0.35, 2.53)	+90.1
MDS-UPDRS Part I								
Hypothetical strategy*	0.69 (0.336) n=57	1.28 (0.604) n=19	-0.27 (0.486) n=48	(-0.89, 0.36)	-39.1	-0.42 (0.777) n=26	(-1.42, 0.59)	-32.8
Treatment policy#	0.06 (0.326) n=80	0.87 (0.634) n=24	0.19 (0.476) n=66	(-0.43, 0.80)	+316.7	0.25 (0.824) n=33	(-0.81, 1.32)	+28.7

Data-driven subphenotype								
	**Placebo**		**Prasinezumab low dose**					
	**Non-diffuse malignant (n=90)**	**Diffuse malignant (n=15)**	**Non-diffuse malignant (n=84)**			**Diffuse malignant (n=21)**		
	**Adjusted mean (SE)**	**Adjusted mean (SE)**	**Difference in adjusted means (SE)**	**80% CI**	**%RR**	**Difference in adjusted means (SE)**	**80% CI**	**%RR**
MDS-UPDRS Part III								
Hypothetical strategy*	4.76 (0.910) n=68	12.29 (3.450) n=8	-1.19 (1.291) n=63	(-2.85, 0.47)	-25.0	-8.40 (4.435) n=11	(-14.2, -2.59)	-68.3
Treatment policy OFF#	3.29 (0.829) n=86	5.17 (2.401) n=15	-0.68 (1.170) n=81	(-2.18, 0.82)	-20.7	-3.11 (3.185) n=19	(-7.24, 1.03)	-60.2
Treatment policy ON#	2.51 (0.868) n=89	3.81 (2.598) n=16	-0.83 (1.230) n=82	(-2.41, 0.75)	-33.1	-4.03 (3.443) n=20	(-8.49, 0.44)	-105.8
MDS-UPDRS Part II								
Hypothetical strategy*	2.47 (0.373) n=68	4.08 (1.533) n=8	0.34 (0.530) n=63	(-0.34, 1.02)	+13.8	-0.11 (1.965) n=11	(-2.69, 2.47)	-2.7
Treatment policy#	1.48 (0.347) n=90	1.57 (1.334) n=15	0.27 (0.495) n=82	(-0.36, 0.91)	+18.2	0.55 (1.748) n=20	(-1.72, 2.81)	+35.0
MDS-UPDRS Part I								
Hypothetical strategy*	0.76 (0.288) n=68	1.59 (1.125) n=8	-0.33 (0.405) n=63	(-0.86, 0.19)	-43.4	-0.49 (1.430) n=11	(-2.36, 1.39)	-30.8
Treatment policy#	0.35 (0.274) n=89	0.10 (1.023) n=15	0.01 (0.388) n=79	(-0.49, 0.51)	+2.9	1.38 (1.320) n=20	(-0.33, 3.10)	+1380
Age at baseline								

	Placebo		Prasinezumab low dose					
	<60 years (n=50)	≥60 years (n=55)	<60 years (n=48)			≥60 years (n=57)		
	Adjusted mean (SE)	Adjusted mean (SE)	Difference in adjusted means (SE)	80% CI	%RR	Difference in adjusted means (SE)	80% CI	%RR
MDS-UPDRS Part III								
Hypothetical strategy*	3.83 (1.246) n=36	7.04 (1.304) n=40	-1.22 (1.795) n=32	(-3.53, 1.10)	-31.9	-2.25 (1.798) n=42	(-4.57, 0.07)	-32.0
Treatment policy OFF#	2.27 (1.131) n=49	4.74 (1.162) n=52	-0.36 (1.602) n=47	(-2.42, 1.70)	-15.9	-1.37 (1.607) n=53	(-3.44, 0.70)	-28.9
Treatment policy ON#	1.52 (1.211) n=50	3.79 (1.204) n=55	-1.12 (1.726) n=47	(-3.34, 1.11)	-73.7	-1.37 (1.670) N=55	(-3.52, 0.78)	-36.1
MDS-UPDRS Part II								
Hypothetical strategy*	2.31 (0.531) n=36	3.04 (0.521) n=40	0.34 (0.758) n=32	(-0.64, 1.32)	+14.7	0.31 (0.717) n=42	(-0.61, 1.24)	+10.2
Treatment policy#	1.20 (0.490) n=50	1.65 (0.504) n=55	0.55 (0.694) n=47	(-0.35, 1.44)	+45.8	0.21 (0.702) n=55	(-0.69, 1.12)	+12.7
MDS-UPDRS Part I								
Hypothetical strategy*	0.30 (0.385) n=36	1.21 (0.442) n=40	0.03 (0.545) n=32	(-0.67, 0.73)	+10	-0.35 (0.602) n=42	(-1.13, 0.43)	-28.9
Treatment policy#	–0.26 (0.432) n=49	0.54 (0.392) n=55	0.77 (0.611) n=45	(-0.01, 1.56)	-296.2	0.11 (0.540) n=54	(-0.59, 0.81)	+20.4
Sex								
	Placebo		Prasinezumab low dose					

	Female (n=34)	Male (n=71)	Female (n=34)			Male (n=71)		
	Adjusted mean (SE)	Adjusted mean (SE)	Difference in adjusted means (SE)	80% CI	%RR	Difference in adjusted means (SE)	80% CI	%RR
MDS-UPDRS Part III								
Hypothetical strategy*	4.20 (1.647) n=26	6.18 (1.066) n=50	-1.89 (2.301) n=28	(-4.86, 1.08)	-45.0	-1.29 (1.513) n=46	(-3.23, 0.66)	-20.9
Treatment policy OFF#	3.95 (1.528) n=33	3.49 (0.934) n=68	-2.06 (2.153) n=33	(-4.84, 0.72)	-52.2	-0.30 (1.316) n=67	(-1.99, 1.39)	-8.6
Treatment policy ON#	3.14 (1.521) n=34	2.57 (1.012) n=71	-1.87 (2.154) n=33	(-4.65, 0.91)	-59.6	-0.90 (1.429) n=69	(-2.74, 0.94)	-35.0
MDS-UPDRS Part II								
Hypothetical strategy*	1.26 (0.555) n=26	3.69 (0.492) n=50	1.00 (0.779) n=28	(-0.01, 2.01)	+79.4	-0.05 (0.695) n=46	(-0.95, 0.84)	-1.4
Treatment policy#	0.43 (0.508) n=34	2.03 (0.454) n=71	1.31 (0.720) n=33	(0.38, 2.24)	+304.7	-0.05 (0.642) n=69	(-0.88, 0.78)	-2.5
MDS-UPDRS Part I								
Hypothetical strategy*	–0.41 (0.500) n=26	1.23 (0.351) n=50	1.01 (0.697) n=28	(0.10, 1.91)	-246.3	-0.74 (0.498) n=46	(-1.38, -0.10)	-60.2
Treatment policy#	–0.40 (0.599) n=34	0.37 (0.296) n=70	1.22 (0.848) n=33	(0.12, 2.31)	-305.0	-0.04 (0.419) n=66	(-0.58, 0.50)	-10.8
Disease duration								
	Placebo		Prasinezumab low dose					
	<12 months	>12 months	<12 months			>12 months		

	(n=72)	(n=33)	(n=70)			(n=35)		
	Adjusted mean (SE)	Adjusted mean (SE)	Difference in adjusted means (SE)	80% CI	%RR	Difference in adjusted means (SE)	80% CI	%RR
MDS-UPDRS Part III								
Hypothetical strategy*	5.60 (1.084) n=55	5.98 (1.748) n=21	-1.72 (1.524) n=50	(-3.68, 0.24)	-30.7	-2.33 (2.379) n=24	(-5.41, 0.74)	-39.0
Treatment policy OFF#	3.49 (1.002) n=68	4.18 (1.383) n=33	-1.03 (1.401) n=65	(-2.83, 0.77)	-29.5	-0.99 (1.901) n=35	(-3.45, 1.46)	-23.7
Treatment policy ON#	2.59 (1.037) n=72	3.14 (1.506) n=33	-1.48 (1.457) n=67	(-3.35, 0.39)	-57.1	-0.86 (2.073) n=35	(-3.54, 1.82)	-27.4
MDS-UPDRS Part II								
Hypothetical strategy*	2.60 (0.477) n=55	3.27 (0.632) n=21	0.52 (0.673) n=50	(-0.35, 1.38)	+20.0	-0.34 (0.872) n=24	(-1.47, 0.79)	-10.4
Treatment policy#	1.33 (0.455) n=72	1.79 (0.545) n=33	0.52 (0.642) n=67	(-0.30, 1.35)	+39.1	0.11 (0.757) n=35	(-0.87, 1.08)	+6.1
MDS-UPDRS Part I								
Hypothetical strategy*	0.42 (0.364) n=55	1.81 (0.518) n=21	0.36 (0.508) n=50	(-0.29, 1.02)	+85.7	-1.61 (0.703) n=24	(-2.52, -0.70)	-89.0
Treatment policy#	0.05 (0.372) n=72	0.76 (0.440) n=32	0.68 (0.524) n=65	(0.01, 1.36)	+1360.0	-0.48 (0.605) n=34	(-1.26, 0.30)	-63.2
Age at diagnosis								
	**Placebo**		**Prasinezumab low dose**					
	**<60 years (n=52)**	**≥60 years (n=53)**	**<60 years (n=50)**			**≥60 years (n=55)**		

	Adjusted mean (SE)	Adjusted mean (SE)	Difference in adjusted means (SE)	80% CI	%RR	Difference in adjusted means (SE)	80% CI	%RR
MDS-UPDRS Part III								
Hypothetical strategy*	3.74 (1.218) n=37	7.18 (1.331) n=39	-1.41 (1.746) n=34	(-3.66, 0.84)	-37.7	-2.07 (1.837) n=40	(-4.43, 0.30)	-28.8
Treatment policy OFF#	2.14 (1.104) n=51	4.98 (1.191) n=50	-0.40 (1.565) n=49	(-2.42, 1.62)	-18.7	-1.39 (1.639) n=51	(-3.50, 0.72)	-27.9
Treatment policy ON#	1.40 (1.181) n=52	3.97 (1.229) n=53	-1.09 (1.684) n=49	(-3.26, 1.08)	-77.9	-1.39 (1.698) n=53	(-3.57, 0.80)	-35.0
MDS-UPDRS Part II								
Hypothetical strategy*	2.16 (0.515) n=37	3.20 (0.536) n=39	0.43 (0.734) n=34	(-0.52, 1.37)	+19.9	0.24 (0.735) n=40	(-0.71, 1.19)	+7.5
Treatment policy#	1.11 (0.476) n=52	1.75 (0.521) n=53	0.61 (0.676) n=49	(-0.26, 1.48)	+55.0	0.16 (0.723) n=53	(-0.77, 1.09)	+9.1
MDS-UPDRS Part I								
Hypothetical strategy*	0.36 (0.373) n=37	1.15 (0.455) n=39	-0.05 (0.528) n=34	(-0.73, 0.63)	-13.9	-0.22 (0.618) n=40	(-1.02, 0.57)	-19.1
Treatment policy#	–0.18 (0.416) n=51	0.47 (0.406) n=53	0.68 (0.591) n=47	(-0.08, 1.44)	-377.8	0.21 (0.556) n=52	(-0.51, 0.92)	44.7
Motor subphenotypes akinetic rigid and tremor-dominant								
	**Placebo**		**Prasinezumab low dose**					
	**Tremor dominant (n=41)**	**Akinetic-rigid (n=52)**	**Tremor dominant (n=38)**			**Akinetic-rigid (n=57)**		
	**Adjusted mean**	**Adjusted mean**	**Difference in**	**80% CI**	**%RR**	**Difference in**	**80% CI**	**%RR**

	(SE)	(SE)	adjusted means (SE)			adjusted means (SE)		
MDS-UPDRS Part III								
Hypothetical strategy*	4.93 (1.386) n=26	6.00 (1.206) n=40	-1.79 (1.924) n=26	(-4.28, 0.69)	-36.3	-2.65 (1.679) n=40	(-4.81, -0.48)	-44.2
Treatment policy OFF#	3.18 (1.235) n=40	4.17 (1.076) n=50	0.08 (1.764) n=35	(-2.20, 2.35)	+2.5	-2.21 (1.473) n=55	(-4.11, -0.32)	-53.0
Treatment policy ON#	1.48 (1.337) n=41	3.84 (1.125) n=52	0.25 (1.918) n=36	(-2.22, 2.73)	+16.9	-2.89 (1.546) n=56	(-4.88, -0.90)	-75.3
MDS-UPDRS Part II								
Hypothetical strategy*	3.49 (0.580) n=26	1.83 (0.507) n=40	-1.21 (0.799) n=26	(-2.25, -0.18)	-34.7	1.99 (0.701) n=40	(1.08, 2.89)	+108.7
Treatment policy#	1.49 (0.522) n=41	1.32 (0.528) n=52	-0.09 (0.744) n=36	(-1.05, 0.86)	-6.0	1.03 (0.727) n=56	(0.09, 1.96)	+78.0
MDS-UPDRS Part I								
Hypothetical strategy*	0.87 (0.459) n=26	0.30 (0.429) n=40	-0.52 (0.637) n=26	(-1.34, 0.30)	-59.8	0.48 (0.590) n=40	(-0.28, 1.24)	+160.0
Treatment policy#	0.43 (0.476) n=41	–0.22 (0.396) n=51	0.09 (2.256) n=35	(-2.81, 3.00)	+20.9	-0.51 (2.189) n=53	(-3.32, 2.31)	+231.8
Motor subphenotypes PIGD and tremor dominant								
	**Placebo**		**Prasinezumab low dose**					
	**Tremor dominant (n=77)**	**PIGD (n=23)**	**Tremor dominant (n=72)**			**PIGD (n=24)**		
	**Adjusted mean (SE)**	**Adjusted mean (SE)**	**Difference in adjusted means (SE)**	**80% CI**	**%RR**	**Difference in adjusted means (SE)**	**80% CI**	**%RR**

MDS-UPDRS Part III								
Hypothetical strategy*	4.70 (1.108) n=53	8.40 (1.588) n=19	-1.22 (1.554) n=52	(-3.22, 0.78)	-26.0	-4.53 (2.233) n=16	(-7.44, -1.61)	-53.9
Treatment policy OFF#	2.88 (0.974) n=74	5.88 (1.425) n=22	-0.07 (1.384) n=69	(-1.85, 1.71)	-2.4	-3.30 (1.932) n=23	(-5.80, -0.79)	-56.1
Treatment policy ON#	1.80 (1.007) n=77	5.43 (1.570) n=23	-0.17 (1.438) n=70	(-2.02, 1.68)	-9.4	-4.36 (2.139) n=24	(-7.13, -1.59)	-80.3
MDS-UPDRS Part II								
Hypothetical strategy*	3.23 (0.453) n=53	1.31 (0.698) n=19	-0.60 (0.635) n=52	(-1.42, 0.22)	-18.6	2.81 (0.987) n=16	(1.53, 4.10)	+214.5
Treatment policy#	1.74 (0.400) n=77	0.83 (0.755) n=23	-0.11 (0.571) n=70	(-0.84, 0.63)	-6.3	1.29 (1.046) n=24	(-0.06, 2.65)	+155.4
MDS-UPDRS Part I								
Hypothetical strategy*	0.94 (0.346) n=53	0.09 (0.690) n=19	-0.55 (0.483) n=52	(-1.17, 0.07)	-58.5	0.93 (0.959) n=16	(-0.32, 2.18)	+1033.3
Treatment policy#	0.40 (0.345) n=77	–0.54 (0.646) n=22	0.04 (0.492) n=69	(-0.59, 0.68)	+10.0	1.28 (0.871) n=23	(0.15, 2.41)	-237.0

* ‘Hypothetical strategy’ assumes a scenario in which the events of start of symptomatic therapy or change in MAO-B inhibitor dose did not occur (performed for the mITT population)
# ‘Treatment policy strategy’ in which the treatment effect is estimated irrespective of symptomatic treatment start or changes in MAO-B inhibitor treatment (performed for the ITT population)

CI, confidence interval; MAO-B, monoamine oxidase-B;MDS-UPDRS, Movement Disorder Society-Unified Parkinson’s Disease Rating Scale; ITT, intention-to-treat; mITT, modified intention-to-treat; PIGD, postural instability gait dysfunction; RBDSQ, Rapid Eye Movement Sleep Behavior Disorder Screening Questionnaire; REM, rapid eye movement; %RR, percentage relative reduction; SE, standard error.

Supplementary Table 4: Change from baseline at Week 52 for placebo and prasinezumab high dose in all subpopulations

MAO-B inhibitor at baseline								
	**Placebo**		**Prasinezumab high dose**					
	**Treatment-naïve (n=67)**	**MAO-B (n=38)**	**Treatment-naïve (n=67)**			**MAO-B (n=39)**		
	**Adjusted mean (SE)**	**Adjusted mean (SE)**	**Difference in adjusted means (SE)**	**80% CI**	**%RR**	**Difference in adjusted means (SE)**	**80% CI**	**%RR**
MDS-UPDRS Part III								
Hypothetical strategy*	5.04 (1.163) n=48	6.82 (1.371) n=28	-1.38 (1.636) n=47	(-3.49, 0.72)	-27.4	-0.28 (1.962) n=26	(-2.82, 2.25)	-4.1
Treatment policy OFF#	3.10 (1.048) n=65	4.79 (1.214) n=36	0.35 (1.476) n=62	(-1.54, 2.25)	+11.3	-1.47 (1.689) n=39	(-3.65, 0.71)	-30.7
Treatment policy ON#	2.01 (1.109) n=67	4.18 (1.248) n=38	0.34 (1.559) n=65	(-1.66, 2.35)	+16.9	-1.25 (1.753) n=39	(-3.51, 1.01)	-29.9
MDS-UPDRS Part II								
Hypothetical strategy*	2.89 (0.467) n=48	2.40 (0.635) n=28	-0.01 (0.657) n=47	(-0.85, 0.84)	-0.3	-0.04 (0.890) n=26	(-1.19, 1.11)	-1.7
Treatment policy#	1.63 (0.438) n=67	1.21 (0.592) n=38	0.23 (0.618) n=64	(-0.56, 1.03)	+14.1	-0.04 (0.825) n=39	(-1.10, 1.03)	-3.3
MDS-UPDRS Part I								
Hypothetical strategy*	0.38 (0.371) n=48	1.28 (0.466) n=28	0.33 (0.519) n=47	(-0.33, 1.00)	+86.8	-0.09 (0.660) n=26	(-0.94, 0.76)	-7.0
Treatment	0.10 (0.375)	0.50 (0.452)	0.67 (0.528)	(-0.01, 1.35)	+670.0	0.26 (0.630)	(-0.56, 1.07)	+52.0

policy#	n=67	n=37	n=63			n=39		
Hoehn and Yahr stage								
	**Placebo**		**Prasinezumab high dose**					
	**Stage 1 (n=20)**	**Stage 2 (n=85)**	**Stage 1 (n=29)**			**Stage 2 (n=77)**		
	**Adjusted mean (SE)**	**Adjusted mean (SE)**	**Difference in adjusted means (SE)**	**80% CI**	**%RR**	**Difference in adjusted means (SE)**	**80% CI**	**%RR**
MDS-UPDRS Part III								
Hypothetical strategy*	2.17 (1.836) n=17	6.34 (1.038) n=59	0.87 (2.456) n=22	(-2.31, 4.05)	+40.1	-1.30 (1.479) n=54	(-3.20, 0.60)	-20.5
Treatment policy OFF#	1.60 (1.779) n=19	3.79 (0.908) n=82	1.31 (2.301) n=27	(-1.66, 4.29)	+81.9	-0.26 (1.296) n=74	(-1.93, 1.40)	-6.9
Treatment policy ON#	1.18 (1.863) n=20	2.79 (0.952) n=85	1.62 (2.420) n=28	(-1.51, 4.75)	+137.3	-0.24 (1.363) n=76	(-1.99, 1.51)	-8.6
MDS-UPDRS Part II								
Hypothetical strategy*	1.68 (0.703) n=17	3.01 (0.434) n=59	0.41 (0.943) n=19	(-0.81, 1.63)	+24.4	-0.19 (0.619) n=54	(-0.98, 0.61)	-6.3
Treatment policy#	0.97 (0.671) n=20	1.62 (0.413) n=85	0.36 (0.877) n=28	(-0.77, 1.50)	+37.1	0.07 (0.593) n=75	(-0.70, 0.83)	+4.3
MDS-UPDRS Part I								
Hypothetical strategy*	–0.18 (0.645) n=17	1.05 (0.334) n=59	1.40 (0.867) n=19	(0.28, 2.53)	-777.8	-0.43 (0.474) n=54	(-1.04, 0.18)	-41.0
Treatment policy#	–0.36 (0.643) n=19	0.36 (0.326) n=85	1.50 (0.832) n=27	(0.43, 2.58)	-416.7	0.15 (0.466) n=75	(-0.45, 0.75)	+41.7

REM sleep behavior disorder score (RBDSQ)								
	**Placebo**		**Prasinezumab high dose**					
	**<5 (n=81)**	**≥5 (n=24)**	**<5 (n=78)**			**≥5 (n=27)**		
	**Adjusted mean (SE)**	**Adjusted mean (SE)**	**Difference in adjusted means (SE)**	**80% CI**	**%RR**	**Difference in adjusted means (SE)**	**80% CI**	**%RR**
MDS-UPDRS Part III								
Hypothetical strategy*	4.98 (1.008) n=57	7.76 (2.008) n=19	-0.44 (1.451) n=50	(-2.31, 1.43)	-8.8	-2.66 (2.682) n=23	(-6.13, 0.81)	-34.3
Treatment policy OFF#	3.00 (0.885) n=78	5.44 (1.809) n=23	0.05 (1.251) n=75	(-1.56, 1.66)	+1.7	-1.57 (2.453) n=26	(-4.74, 1.60)	-28.9
Treatment policy ON#	2.38 (0.935) n=81	3.64 (1.839) n=24	-0.40 (1.326) n=77	(-2.10, 1.31)	-16.8	0.26 (2.494) n=27	(-2.96, 3.48)	+7.1
MDS-UPDRS Part II								
Hypothetical strategy*	2.82 (0.417) n=57	2.19 (0.857) n=19	-0.21 (0.596) n=50	(-0.98, 0.55)	-7.4	0.78 (1.150) n=23	(-0.71, 2.27)	+35.6
Treatment policy#	1.51 (0.379) n=81	1.21 (0.863) n=24	-0.08 (0.537) n=76	(-0.77, 0.61)	-5.3	0.77 (1.173) n=27	(-0.74, 2.29)	+63.6
MDS-UPDRS Part I								
Hypothetical strategy*	0.69 (0.336) n=57	1.28 (0.604) n=19	0.19 (0.483) n=50	(-0.43, 0.82)	+27.5	-0.37 (0.794) n=23	(-1.40, 0.65)	-28.9
Treatment policy#	0.06 (0.326) n=80	0.87 (0.634) n=24	0.65 (0.462) n=75	(0.05, 1.24)	+1083.3	-0.20 (0.858) n=27	(-1.31, 0.91)	-23.0
Data-driven subphenotype								

	Placebo		Prasinezumab high dose					
	Non-diffuse malignant (n=90)	Diffuse malignant (n=15)	Non-diffuse malignant (n=83)			Diffuse malignant (n=23)		
	Adjusted mean (SE)	Adjusted mean (SE)	Difference in adjusted means (SE)	80% CI	%RR	Difference in adjusted means (SE)	80% CI	%RR
MDS-UPDRS Part III								
Hypothetical strategy*	4.76 (0.910) n=68	12.29 (3.450) n=8	-0.33 (1.312) n=59	(-2.02, 1.35)	-6.9	-7.77 (4.296) n=14	(-13.4, -2.14)	-63.2
Treatment policy OFF#	3.29 (0.829) n=86	5.17 (2.401) n=15	-0.20 (1.174) n=80	(-1.70, 1.31)	-6.1	-2.09 (3.161) n=21	(-6.19, 2.01)	-40.4
Treatment policy ON#	2.51 (0.868) n=89	3.81 (2.598) n=16	-0.16 (1.235) n=81	(-1.75, 1.43)	-6.4	-1.64 (3.388) n=23	(-6.03, 2.76)	-43.0
MDS-UPDRS Part II								
Hypothetical strategy*	2.47 (0.373) n=68	4.08 (1.533) n=8	0.08 (0.534) n=59	(-0.61, 0.77)	+3.2	-1.16 (1.940) n=14	(-3.70, 1.39)	-28.4
Treatment policy#	1.48 (0.347) n=90	1.57 (1.334) n=15	0.12 (0.496) n=81	(-0.52, 0.76)	+8.1	-0.41 (1.715) n=22	(-2.63, 1.82)	-26.1
MDS-UPDRS Part I								
Hypothetical strategy*	0.76 (0.288) n=68	1.59 (1.125) n=8	-0.05 (0.413) n=59	(-0.59, 0.48)	-6.6	-0.56 (1.398) n=14	(-2.39, 1.28)	-35.2
Treatment policy#	0.35 (0.274) n=89	0.10 (1.023) n=15	0.30 (0.389) n=81	(-0.19, 0.80)	+85.7	0.53 (1.322) n=21	(-1.19, 2.25)	+530.0
Age at baseline								
	**Placebo**		**Prasinezumab high dose**					

	<60 years (n=50)	≥60 years (n=55)	<60 years (n=50)			≥60 years (n=56)		
	**Adjusted mean (SE)**	**Adjusted mean (SE)**	**Difference in adjusted means (SE)**	**80% CI**	**%RR**	**Difference in adjusted means (SE)**	**80% CI**	**%RR**
MDS-UPDRS Part III								
Hypothetical strategy*	3.83 (1.246) n=36	7.04 (1.304) n=40	-0.04 (1.778) n=32	(-2.33, 2.26)	-1.0	-1.57 (1.805) n=41	(-3.89, 0.76)	-22.3
Treatment policy OFF#	2.27 (1.131) n=49	4.74 (1.162) n=52	0.16 (1.581) n=48	(-1.87, 2.20)	+7.0	-0.66 (1.622) n=53	(-2.74, 1.43)	-13.9
Treatment policy ON#	1.52 (1.211) n=50	3.79 (1.204) n=55	0.46 (1.698) n=49	(-1.73, 2.65)	+30.3	-0.77 (1.681) n=55	(-2.94, 1.39)	-20.3
MDS-UPDRS Part II								
Hypothetical strategy*	2.31 (0.531) n=36	3.04 (0.521) n=40	0.01 (0.759) n=32	(-0.96, 0.99)	+0.4	-0.03 (0.719) n=41	(-0.96, 0.90)	-1.0
Treatment policy#	1.20 (0.490) n=50	1.65 (0.504) n=55	0.67 (0.690) n=48	(-0.22, 1.56)	+55.8	-0.37 (0.703) n=55	(-1.27, 0.54)	-22.4
MDS-UPDRS Part I								
Hypothetical strategy*	0.30 (0.385) n=36	1.21 (0.442) n=40	0.32 (0.552) n=32	(-0.39, 1.04)	+106.7	-0.06 (0.605) n=41	(-0.84, 0.72)	-5.0
Treatment policy#	–0.26 (0.432) n=49	0.54 (0.392) n=55	0.88 (0.608) n=48	(0.10, 1.67)	-338.5	0.29 (0.541) n=54	(-0.41, 0.98)	+53.7
Sex								
	**Placebo**		**Prasinezumab high dose**					
	**Female (n=34)**	**Male (n=71)**	**Female (n=35)**			**Male (n=71)**		

	Adjusted mean (SE)	Adjusted mean (SE)	Difference in adjusted means (SE)	80% CI	%RR	Difference in adjusted means (SE)	80% CI	%RR
MDS-UPDRS Part III								
Hypothetical strategy*	4.20 (1.647) n=26	6.18 (1.066) n=50	-0.41 (2.407) n=22	(-3.51, 2.70)	-9.8	-1.18 (1.489) n=51	(-3.10, 0.74)	+19.1
Treatment policy OFF#	3.95 (1.528) n=33	3.49 (0.934) n=68	-0.78 (2.137) n=34	(-3.53, 1.98)	-19.7	-0.06 (1.320) n=67	(-1.76, 1.64)	-1.7
Treatment policy ON#	3.14 (1.521) n=34	2.57 (1.012) n=71	-0.11 (2.131) n=35	(-2.86, 2.64)	-3.5	-0.30 (1.433) n=69	(-2.14, 1.54)	-11.7
MDS-UPDRS Part II								
Hypothetical strategy*	1.26 (0.555) n=26	3.69 (0.492) n=50	1.10 (0.806) n=22	(0.06, 2.14)	+87.3	-0.85 (0.687) n=51	(-1.74, 0.03)	+23.0
Treatment policy#	0.43 (0.508) n=34	2.03 (0.454) n=71	1.23 (0.712) n=35	(0.31, 2.15)	+286.0	-0.48 (0.643) n=68	(-1.31, 0.34)	-23.6
MDS-UPDRS Part I								
Hypothetical strategy*	–0.41 (0.500) n=26	1.23 (0.351) n=50	2.34 (0.732) n=22	(1.40, 3.29)	-570.7	-0.84 (0.489) n=51	(-1.47, -0.21)	-68.3
Treatment policy#	–0.40 (0.599) n=34	0.37 (0.296) n=70	2.40 (0.844) n=34	(1.31, 3.48)	-600.0	-0.44 (0.419) n=68	(-0.98, 0.09)	-118.9
Disease duration								
	**Placebo**		**Prasinezumab high dose**					
	**<12 months (n=72)**	**>12 months (n=33)**	**<12 months (n=77)**			**>12 months (n=29)**		
	**Adjusted mean (SE)**	**Adjusted mean (SE)**	**Difference in adjusted**	**80% CI**	**%RR**	**Difference in adjusted**	**80% CI**	**%RR**

			means (SE)			means (SE)		
MDS-UPDRS Part III								
Hypothetical strategy*	5.60 (1.084) n=55	5.98 (1.748) n=21	-1.85 (1.496) n=55	(-3.77, 0.08)	-33.0	0.80 (2.599) n=18	(-2.56, 4.16)	+13.4
Treatment policy OFF#	3.49 (1.002) n=68	4.18 (1.383) n=33	-1.14 (1.362) n=74	(-2.89, 0.61)	-32.7	1.30 (2.078) n=27	(-1.38, 3.98)	+31.1
Treatment policy ON#	2.59 (1.037) n=72	3.14 (1.506) n=33	-0.68 (1.416) n=77	(-2.50, 1.14)	-26.3	0.68 (2.268) n=27	(-2.25, 3.61)	+21.7
MDS-UPDRS Part II								
Hypothetical strategy*	2.60 (0.477) n=55	3.27 (0.632) n=21	0.45 (0.658) n=55	(-0.40, 1.30)	+17.3	-1.40 (0.924) n=18	(-2.60, -0.21)	-42.8
Treatment policy#	1.33 (0.455) n=72	1.79 (0.545) n=33	0.39 (0.624) n=76	(-0.41, 1.19)	+29.3	-0.50 (0.804) n=27	(-1.53, 0.54)	-27.9
MDS-UPDRS Part I								
20,6Hypothetical strategy*	0.42 (0.364) n=55	1.81 (0.518) n=21	0.78 (0.498) n=55	(0.14, 1.42)	+185.7	-1.91 (0.766) n=18	(-2.90, -0.92)	-106.5
Treatment policy#	0.05 (0.372) n=72	0.76 (0.440) n=32	0.88 (0.508) n=75	(0.23, 1.53)	+1760.0	-0.68 (0.655) n=27	(-1.53, 0.17)	-89.5
Age at diagnosis								
	**Placebo**		**Prasinezumab high dose**					
	**<60 years (n=52)**	**≥60 years (n=53)**	**<60 years (n=52)**			**≥60 years (n=54)**		
	**Adjusted mean (SE)**	**Adjusted mean (SE)**	**Difference in adjusted means (SE)**	**80% CI**	**%RR**	**Difference in adjusted means (SE)**	**80% CI**	**%RR**

MDS-UPDRS Part III								
Hypothetical strategy*	3.74 (1.218) n=37	7.18 (1.331) n=39	0.40 (1.748) n=32	(-1.86, 2.65)	+10.7	-2.06 (1.832) n=41	(-4.42, 0.30)	-28.7
Treatment policy OFF#	2.14 (1.104) n=51	4.98 (1.191) n=50	0.68 (1.545) n=50	(-1.31, 2.67)	+31.8	-1.21 (1.659) n=51	(-3.34, 0.93)	-24.3
Treatment policy ON#	1.40 (1.181) n=52	3.97 (1.229) n=53	0.62 (1.658) n=51	(-1.51, 2.75)	+44.3	-0.94 (1.715) n=53	(-3.14, 1.27)	-23.7
MDS-UPDRS Part II								
Hypothetical strategy*	2.16 (0.515) n=37	3.20 (0.536) n=39	0.19 (0.739) n=32	(-0.76, 1.14)	+8.8	-0.16 (0.735) n=41	(-1.11, 0.79)	-5.0
Treatment policy#	1.11 (0.476) n=52	1.75 (0.521) n=53	0.73 (0.672) n=50	(-0.13, 1.60)	+65.8	-0.46 (0.726) n=53	(-1.39, 0.47)	-26.3
MDS-UPDRS Part I								
Hypothetical strategy*	0.36 (0.373) n=37	1.15 (0.455) n=39	0.30 (0.538) n=32	(-0.39, 1.00)	+83.3	0.01 (0.617) n=41	(-0.79, 0.80)	+0.9
Treatment policy#	–0.18 (0.416) n=51	0.47 (0.406) n=53	0.79 (0.586) n=50	(0.03, 1.54)	-438.9	0.36 (0.558) n=52	(-0.36, 1.08)	+76.6
Motor subphenotypes akinetic rigid and tremor-dominant								
	**Placebo**		**Prasinezumab high dose**					
	**Tremor dominant (n=41)**	**Akinetic-rigid (n=52)**	**Tremor dominant (n=43)**			**Akinetic-rigid (n=56)**		
	**Adjusted mean (SE)**	**Adjusted mean (SE)**	**Difference in adjusted means (SE)**	**80% CI**	**%RR**	**Difference in adjusted means (SE)**	**80% CI**	**%RR**
MDS-UPDRS Part III								

Hypothetical strategy*	4.93 (1.386) n=26	6.00 (1.206) n=40	1.26 (1.846) n=33	(-1.13, 3.64)	+25.6	-2.59 (1.697) n=37	(-4.77, -0.40)	-43.2
Treatment policy OFF#	3.18 (1.235) n=40	4.17 (1.076) n=50	2.30 (1.696) n=42	(0.11, 4.48)	+72.3	-2.25 (1.474) n=54	(-4.14, -0.35)	-54.0
Treatment policy ON#	1.48 (1.337) n=41	3.84 (1.125) n=52	3.42 (1.844) n=43	(1.04, 5.79)	+231.1	-2.81 (1.547) n=55	(-4.80, -0.82)	-73.2
MDS-UPDRS Part II								
Hypothetical strategy*	3.49 (0.580) n=26	1.83 (0.507) n=40	-1.80 (0.780) n=33	(-2.81, -0.79)	-51.6	1.44 (0.710) n=37	(0.52, 2.35)	+78.7
Treatment policy#	1.49 (0.522) n=41	1.32 (0.528) n=52	0.10 (0.721) n=42	(-0.83, 1.03)	+6.7	0.13 (0.729) n=55	(-0.81, 1.07)	+9.8
MDS-UPDRS Part I								
Hypothetical strategy*	0.87 (0.459) n=26	0.30 (0.429) n=40	-0.17 (0.607) n=33	(-0.95, 0.62)	-19.5	0.77 (0.603) n=37	(-0.01, 1.55)	+256.7
Treatment policy#	0.43 (0.476) n=41	–0.22 (0.396) n=51	1.96 (2.191) n=40	(-0.87, 4.78)	+455.8	-1.41 (2.224) n=50	(-4.27, 1.45)	+640.9
Motor subphenotypes PIGD and tremor dominant								
	**Placebo**		**Prasinezumab high dose**					
	**Tremor dominant (n=77)**	**PIGD (n=23)**	**Tremor dominant (n=72)**			**PIGD (n=23)**		
	**Adjusted mean (SE)**	**Adjusted mean (SE)**	**Difference in adjusted means (SE)**	**80% CI**	**%RR**	**Difference in adjusted means (SE)**	**80% CI**	**%RR**
MDS-UPDRS Part III								
Hypothetical strategy*	4.70 (1.108) n=53	8.40 (1.588) n=19	0.34 (1.572) n=51	(-1.68, 2.36)	+7.2	-5.42 (2.255) n=15	(-8.37, -2.48)	-64.5

Treatment policy OFF#	2.88 (0.974) n=74	5.88 (1.425) n=22	1.01 (1.385) n=69	(-0.77, 2.79)	+35.1	-4.55 (1.982) n=21	(-7.12, -1.98)	-77.4
Treatment policy ON#	1.80 (1.007) n=77	5.43 (1.570) n=23	1.62 (1.437) n=71	(-0.23, 3.47)	+90.0	-5.41 (2.193) n=22	(-8.25, -2.57)	-99.6
MDS-UPDRS Part II								
Hypothetical strategy*	3.23 (0.453) n=53	1.31 (0.698) n=19	-1.02 (0.644) n=51	(-1.85, -0.19)	-31.6	1.52 (0.977) n=15	(0.25, 2.79)	+116.0
Treatment policy#	1.74 (0.400) n=77	0.83 (0.755) n=23	-0.23 (0.573) n=70	(-0.97, 0.50)	-13.2	-0.16 (1.047) n=22	(-1.51, 1.20)	-19.3
MDS-UPDRS Part I								
Hypothetical strategy*	0.94 (0.346) n=53	0.09 (0.690) n=19	-0.13 (0.488) n=51	(-0.76, 0.50)	-13.8	1.21 (0.973) n=15	(-0.05, 2.48)	+1344.4
Treatment policy#	0.40 (0.345) n=77	–0.54 (0.646) n=22	0.36 (0.493) n=69	(-0.28, 0.99)	+90.0	0.84 (0.886) n=22	(-0.31, 1.99)	-155.6

* 'Hypothetical strategy' assumes a scenario in which the events of start of symptomatic therapy or change in MAO-B inhibitor dose did not occur (performed for the mITT population)

'Treatment policy strategy' in which the treatment effect is estimated irrespective of symptomatic treatment start or changes in MAO-B inhibitor treatment (performed for the ITT population)

CI, confidence interval; MAO-B, monoamine oxidase-B; MDS-UPDRS, Movement Disorder Society-Unified Parkinson's Disease Rating Scale; ITT, intention-to-treat; mITT, modified intention-to-treat; PIGD, postural instability gait dysfunction; RBDSQ, Rapid Eye Movement Sleep Behavior Disorder Screening Questionnaire; REM, rapid eye movement; %RR, percentage relative reduction; SE, standard error.

Supplementary Table 5: Participants free of symptomatic treatment at all visits for placebo and pooled prasinezumab groups− For the whole population and all subpopulations including those taking MAO-B inhibitors at baseline and those who were treatment-naïve at baseline

MAO-B inhibitors at baseline						
	**Placebo**			**Prasinezumab pooled**		
**n (%)**	**Whole population (n=105)**	**Treatment-naïve (n=67)**	**MAO-B inhibitors (n=38)**	**Whole population (n=211)**	**Treatment-naïve (n=134)**	**MAO-B inhibitors (n=77)**
**Baseline**	105 (100) (n=105)	67 (100) (n=67)	38 (100) (n=38)	211 (100) (n=211)	134 (100) (n=134)	77 (100) (n=77)
**Week 8**	104 (100) (n=104)	67 (100) (n=67)	37 (100) (n=37)	207 (99.5) (n=208)	131 (99.2) (n=132)	76 (100) (n=76)
**Week 16**	101 (98.1) (n=103)	64 (98.5) (n=65)	37 (97.4) (n=38)	200 (98.0) (n=204)	125 (96.9) (n=129)	75 (100) (n=75)
**Week 24**	96 (93.2) (n=103)	61 (93.8) (n=65)	35 (92.1) (n=38)	191 (93.6) (n=204)	118 (92.2) (n=128)	73 (96.1) (n=76)
**Week 32**	89 (84.8) (n=105)	56 (83.6) (n=67)	33 (86.8) (n=38)	180 (87.4) (n=206)	112 (86.2) (n=130)	68 (89.5) (n=76)
**Week 40**	77 (75.5) (n=102)	48 (73.8) (n=65)	29 (78.4) (n=37)	162 (80.2) (n=202)	99 (78.6) (n=126)	63 (82.9) (n=76)
**Week 48**	77 (73.3) (n=105)	48 (71.6) (n=67)	29 (76.3) (n=38)	155 (75.2) (n=206)	97 (74.6) (n=130)	58 (76.3) (n=76)
**Week 52**	76 (72.4) (n=105)	48 (71.6) (n=67)	28 (73.7) (n=38)	148 (72.2) (n=205)	93 (72.1) (n=129)	55 (72.4) (n=76)

Hoehn and Yahr stage						
	**Placebo**			**Prasinezumab pooled**		
**n (%)**	**Whole population (n=105)**	**Stage 1 (n=20)**	**Stage 2 (n=85)**	**Whole population (n=211)**	**Stage 1 (n=58)**	**Stage 2 (n=153)**
**Baseline**	105 (100) (n=105)	20 (100) (n=20)	85 (100) (n=85)	211 (100) (n=211)	58 (100) (n=58)	153 (100) (n=153)
**Week 8**	104 (100) (n=104)	20 (100) (n=20)	84 (100) (n=84)	207 (99.5) (n=208)	58 (100) (n=58)	149 (99.3) (n=150)
**Week 16**	101 (98.1) (n=103)	20 (100) (n=20)	81 (97.6) (n=83)	200 (98.0) (n=204)	55 (98.2) (n=56)	145 (98.0) (n=148)
**Week 24**	96 (93.2) (n=103)	20 (100) (n=20)	76 (91.6) (n=83)	191 (93.6) (n=204)	55 (96.5) (n=57)	136 (92.5) (n=147)
**Week 32**	89 (84.8) (n=105)	19 (95.0) (n=19)	70 (82.4) (n=85)	180 (87.4) (n=206)	51 (91.1) (n=56)	129 (86.0) (n=150)
**Week 40**	77 (75.5) (n=102)	18 (90.0) (n=20)	59 (72.0) (n=82)	162 (80.2) (n=202)	46 (82.1) (n=56)	116 (79.5) (n=146)
**Week 48**	77 (73.3) (n=105)	18 (90.0) (n=20)	59 (69.4) (n=85)	155 (75.2) (n=206)	44 (78.6) (n=56)	111 (74.0) (n=150)
**Week 52**	76 (72.4) (n=105)	17 (85.0) (n=20)	59 (69.4) (n=85)	148 (72.2) (n=205)	42 (73.7) (n=57)	106 (71.6) (n=148)
REM Sleep behavior disorder score						
	**Placebo**			**Prasinezumab pooled**		
**n (%)**	**Whole population**	**<5**	**≥5**	**Whole population**	**<5**	**≥5**

	(n=105)	(n=81)	(n=24)	(n=211)	(n=149)	(n=61)
**Baseline**	105 (100) (n=105)	81 (100) (n=81)	24 (100) (n=24)	211 (100) (n=211)	149 (100) (n=149)	61 (100) (n=61)
**Week 8**	104 (100) (n=104)	81 (100) (n=81)	23 (100) (n=23)	207 (99.5) (n=208)	146 (100) (n=146)	60 (98.4) (n=61)
**Week 16**	101 (98.1) (n=103)	77 (97.5) (n=79)	24 (100) (n=24)	200 (98.0) (n=204)	142 (97.9) (n=145)	58 (98.3) (n=59)
**Week 24**	96 (93.2) (n=103)	74 (92.5) (n=80)	22 (95.7) (n=23)	191 (93.6) (n=204)	132 (91.7) (n=144)	59 (98.3) (n=60)
**Week 32**	89 (84.8) (n=105)	67 (82.7) (n=81)	22 (91.7) (n=24)	180 (87.4) (n=206)	120 (82.8) (n=145)	60 (98.4) (n=61)
**Week 40**	77 (75.5) (n=102)	58 (74.4) (n=78)	19 (79.2) (n=24)	162 (80.2) (n=202)	107 (74.8) (n=143)	55 (93.2) (n=59)
**Week 48**	77 (73.3) (n=105)	58 (71.6) (n=81)	19 (79.2) (n=24)	155 (75.2) (n=206)	103 (70.5) (n=146)	52 (86.7) (n=60)
**Week 52**	76 (72.4) (n=105)	57 (70.4) (n=81)	19 (79.2) (n=24)	148 (72.2) (n=205)	98 (67.6) (n=145)	50 (83.3) (n=60)
Data-driven subphenotype						
	**Placebo**			**Prasinezumab pooled**		
**n (%)**	**Whole population (n=105)**	**Non-diffuse malignant (n=90)**	**Diffuse malignant (n=15)**	**Whole population (n=211)**	**Non-diffuse malignant (n=167)**	**Diffuse malignant (n=44)**
**Baseline**	105 (100) (n=105)	90 (100) (n=90)	15 (100) (n=15)	211 (100) (n=211)	167 (100) (n=167)	44 (100) (n=44)

**Week 8**	104 (100)<br>(n=104)	90 (100)<br>(n=90)	14 (100)<br>(n=14)	207 (99.5)<br>(n=208)	165 (100)<br>(n=165)	42 (97.7)<br>(n=43)
**Week 16**	101 (98.1)<br>(n=103)	86 (97.7)<br>(n=88)	15 (100)<br>(n=15)	200 (98.0)<br>(n=204)	159 (98.8)<br>(n=161)	41 (95.3)<br>(n=43)
**Week 24**	96 (93.2)<br>(n=103)	83 (94.3)<br>(n=88)	13 (86.7)<br>(n=15)	191 (93.6)<br>(n=204)	155 (95.1)<br>(n=163)	36 (87.8)<br>(n=41)
**Week 32**	89 (84.8)<br>(n=105)	80 (88.9)<br>(n=90)	9 (60.0)<br>(n=15)	180 (87.4)<br>(n=206)	143 (88.3)<br>(n=162)	37 (84.1)<br>(n=44)
**Week 40**	77 (75.5)<br>(n=102)	69 (79.3)<br>(n=87)	8 (53.3)<br>(n=15)	162 (80.2)<br>(n=202)	132 (82.5)<br>(n=160)	30 (71.4)<br>(n=42)
**Week 48**	77 (73.3)<br>(n=105)	69 (76.7)<br>(n=90)	8 (53.3)<br>(n=15)	155 (75.2)<br>(n=206)	127 (77.4)<br>(n=164)	28 (66.7)<br>(n=42)
**Week 52**	76 (72.4)<br>(n=105)	68 (75.6)<br>(n=90)	8 (53.3)<br>(n=15)	148 (72.2)<br>(n=205)	123 (75.5)<br>(n=163)	25 (59.5)<br>(n=42)
Age at baseline						
	**Placebo**			**Prasinezumab pooled**		
**n (%)**	**Whole population (n=105)**	**<60 years (n=50)**	**≥60 years (n=55)**	**Whole population (n=211)**	**<60 years (n=98)**	**≥60 years (n=113)**
**Baseline**	105 (100)<br>(n=105)	50 (100)<br>(n=50)	55 (100)<br>(n=55)	211 (100)<br>(n=211)	98 (100)<br>(n=98)	113 (100)<br>(n=113)
**Week 8**	104 (100)<br>(n=104)	50 (100)<br>(n=50)	54 (100)<br>(n=54)	207 (99.5)<br>(n=208)	97 (100)<br>(n=97)	110 (99.1)<br>(n=110)
**Week 16**	101 (98.1)<br>(n=103)	48 (98.0)<br>(n=49)	53 (98.1)<br>(n=54)	200 (98.0)<br>(n=204)	93 (96.9)<br>(n=96)	107 (99.1)<br>(n=107)

**Week 24**	96 (93.2) (n=103)	46 (93.9) (n=49)	50 (92.6) (n=54)	191 (93.6) (n=204)	87 (91.6) (n=95)	104 (95.4) (n=109)
**Week 32**	89 (84.8) (n=105)	44 (88.0) (n=50)	45 (81.8) (n=55)	180 (87.4) (n=206)	81 (84.4) (n=96)	99 (90.0) (n=110)
**Week 40**	77 (75.5) (n=102)	37 (75.5) (n=49)	40 (75.5) (n=53)	162 (80.2) (n=202)	71 (74.7) (n=95)	91 (85.0) (n=107)
**Week 48**	77 (73.3) (n=105)	37 (74.0) (n=50)	40 (72.7) (n=55)	155 (75.2) (n=206)	69 (71.9) (n=96)	86 (78.2) (n=110)
**Week 52**	76 (72.4) (n=105)	36 (72.0) (n=50)	40 (72.7) (n=55)	148 (72.2) (n=205)	65 (68.4) (n=95)	83 (75.5) (n=110)
Sex						
	**Placebo**			**Prasinezumab pooled**		
**n (%)**	**Whole population (n=105)**	**Female (n=34)**	**Male (n=71)**	**Whole population (n=211)**	**Female (n=69)**	**Male (n=142)**
**Baseline**	105 (100) (n=105)	34 (100) (n=34)	71 (100) (n=71)	211 (100) (n=211)	69 (100) (n=69)	142 (100) (n=142)
**Week 8**	104 (100) (n=104)	34 (100) (n=34)	70 (100) (n=70)	207 (99.5) (n=208)	66 (98.5) (n=67)	141 (100) (n=141)
**Week 16**	101 (98.1) (n=103)	34 (100) (n=34)	67 (97.1) (n=69)	200 (98.0) (n=204)	64 (97.0) (n=66)	136 (98.6) (n=138)
**Week 24**	96 (93.2) (n=103)	31 (93.9) (n=33)	65 (92.9) (n=70)	191 (93.6) (n=204)	65 (94.2) (n=69)	126 (93.3) (n=135)
**Week 32**	89 (84.8) (n=105)	31 (91.2) (n=34)	58 (81.7) (n=71)	180 (87.4) (n=206)	59 (86.8) (n=68)	121 (87.7) (n=138)

**Week 40**	77 (75.5) (n=102)	23 (74.2) (n=31)	54 (76.1) (n=71)	162 (80.2) (n=202)	52 (78.8) (n=66)	110 (80.9) (n=136)
**Week 48**	77 (73.3) (n=105)	26 (76.5) (n=34)	51 (71.8) (n=71)	155 (75.2) (n=206)	51 (73.9) (n=69)	104 (75.9) (n=137)
**Week 52**	76 (72.4) (n=105)	26 (76.5) (n=34)	50 (70.4) (n=71)	148 (72.2) (n=205)	50 (73.5) (n=68)	98 (71.5) (n=137)
Disease duration						
	**Placebo**			**Prasinezumab pooled**		
**n (%)**	**Whole population (n=105)**	**≤12 months (n=72)**	**>12 months (n=33)**	**Whole population (n=211)**	**≤12 months (n=147)**	**>12 months (n=64)**
**Baseline**	105 (100) (n=105)	72 (100) (n=72)	33 (100) (n=33)	211 (100) (n=211)	147 (100) (n=147)	64 (100) (n=64)
**Week 8**	104 (100) (n=104)	71 (100) (n=71)	33 (100) (n=33)	207 (99.5) (n=208)	144 (99.3) (n=145)	63 (100) (n=63)
**Week 16**	101 (98.1) (n=103)	69 (98.6) (n=80)	32 (97.0) (n=33)	200 (98.0) (n=204)	139 (97.9) (n=142)	61 (98.4) (n=62)
**Week 24**	96 (93.2) (n=103)	66 (93.0) (n=71)	30 (93.8) (n=32)	191 (93.6) (n=204)	132 (93.0) (n=142)	59 (95.2) (n=62)
**Week 32**	89 (84.8) (n=105)	62 (86.1) (n=72)	27 (81.8) (n=33)	180 (87.4) (n=206)	125 (86.2) (n=145)	55 (90.2) (n=61)
**Week 40**	77 (75.5) (n=102)	53 (76.8) (n=69)	24 (72.7) (n=33)	162 (80.2) (n=202)	111 (79.3) (n=140)	51 (82.3) (n=62)
**Week 48**	77 (73.3) (n=105)	55 (76.4) (n=72)	22 (66.7) (n=33)	155 (75.2) (n=206)	109 (75.7) (n=144)	46 (74.2) (n=62)

**Week 52**	76 (72.4) (n=105)	55 (76.4) (n=72)	21 (63.6) (n=33)	148 (72.2) (n=205)	105 (73.4) (n=143)	43 (69.4) (n=62)

Age at diagnosis

	**Placebo**			**Prasinezumab pooled**		
**n (%)**	**Whole population (n=105)**	**<60 years (n=52)**	**≥60 years (n=53)**	**Whole population (n=211)**	**<60 years (n=102)**	**≥60 years (n=109)**
**Baseline**	105 (100) (n=105)	52 (100) (n=52)	53 (100) (n=53)	211 (100) (n=211)	102 (100) (n=102)	109 (100) (n=109)
**Week 8**	104 (100) (n=104)	52 (100) (n=52)	52 (100) (n=52)	207 (99.5) (n=208)	100 (100) (n=100)	107 (99.1) (n=108)
**Week 16**	101 (98.1) (n=103)	50 (98.0) (n=51)	51 (98.1) (n=52)	200 (98.0) (n=204)	97 (97.0) (n=97)	103 (99.0) (n=104)
**Week 24**	96 (93.2) (n=103)	48 (94.1) (n=51)	48 (92.3) (n=52)	191 (93.6) (n=204)	91 (91.9) (n=99)	100 (95.2) (n=105)
**Week 32**	89 (84.8) (n=105)	46 (88.5) (n=52)	43 (81.1) (n=53)	180 (87.4) (n=206)	85 (85.0) (n=100)	95 (89.6) (n=106)
**Week 40**	77 (75.5) (n=102)	38 (74.5) (n=51)	39 (76.5) (n=51)	162 (80.2) (n=202)	75 (75.8) (n=99)	87 (84.5) (n=103)
**Week 48**	77 (73.3) (n=105)	38 (73.1) (n=52)	39 (73.6) (n=53)	155 (75.2) (n=206)	71 (71.0) (n=100)	84 (79.2) (n=106)
**Week 52**	76 (72.4) (n=105)	37 (71.2) (n=52)	39 (73.6) (n=53)	148 (72.2) (n=205)	67 (67.7) (n=99)	81 (76.4) (n=106)

Motor subphenotypes akinetic-rigid and tremor dominant

	Placebo			Prasinezumab pooled		
**n (%)**	**Whole population (n=105)**	**Tremor dominant (n=41)**	**Akinetic-rigid (n=52)**	**Whole population (n=211)**	**Tremor dominant (n=81)**	**Akinetic-rigid (n=113)**
**Baseline**	105 (100) (n=105)	41 (100) (n=41)	52 (100) (n=52)	211 (100) (n=211)	81 (100) (n=81)	113 (100) (n=113)
**Week 8**	104 (100) (n=104)	40 (100) (n=40)	52 (100) (n=52)	207 (99.5) (n=208)	80 (100) (n=80)	111 (99.1) (n=112)
**Week 16**	101 (98.1) (n=103)	38 (95.0) (n=40)	51 (100) (n=51)	200 (98.0) (n=204)	76 (97.4) (n=78)	109 (98.2) (n=111)
**Week 24**	96 (93.2) (n=103)	35 (89.7) (n=39)	49 (94.2) (n=52)	191 (93.6) (n=204)	74 (96.1) (n=77)	103 (92.0) (n=112)
**Week 32**	89 (84.8) (n=105)	34 (82.9) (n=41)	45 (86.5) (n=52)	180 (87.4) (n=206)	71 (88.8) (n=80)	95 (86.4) (n=110)
**Week 40**	77 (75.5) (n=102)	27 (67.5) (n=40)	40 (80.0) (n=50)	162 (80.2) (n=202)	63 (82.9) (n=76)	88 (79.3) (n=111)
**Week 48**	77 (73.3) (n=105)	26 (63.4) (n=41)	41 (78.8) (n=52)	155 (75.2) (n=206)	63 (78.8) (n=80)	81 (73.6) (n=110)
**Week 52**	76 (72.4) (n=105)	26 (63.4) (n=41)	40 (76.9) (n=52)	148 (72.2) (n=205)	60 (76.9) (n=78)	77 (69.4) (n=111)
Motor subphenotypes PIGD and tremor dominant						
	**Placebo**			**Prasinezumab pooled**		
**n (%)**	**Whole population (n=105)**	**Tremor dominant (n=77)**	**PIGD (n=23)**	**Whole population (n=211)**	**Tremor dominant (n=144)**	**PIGD (n=47)**

**Baseline**	105 (100) (n=105)	77 (100) (n=77)	23 (100) (n=23)	211 (100) (n=211)	144 (100) (n=144)	47 (100) (n=47)
**Week 8**	104 (100) (n=104)	76 (100) (n=76)	23 (100) (n=23)	207 (99.5) (n=208)	141 (100) (n=141)	46 (97.9) (n=47)
**Week 16**	101 (98.1) (n=103)	74 (97.4) (n=76)	22 (100) (n=22)	200 (98.0) (n=204)	135 (97.8) (n=138)	46 (97.9) (n=47)
**Week 24**	96 (93.2) (n=103)	69 (92.0) (n=75)	22 (95.7) (n=23)	191 (93.6) (n=204)	130 (94.2) (n=138)	43 (91.5) (n=47)
**Week 32**	89 (84.8) (n=105)	63 (81.8) (n=77)	21 (91.3) (n=23)	180 (87.4) (n=206)	124 (87.3) (n=142)	39 (86.7) (n=45)
**Week 40**	77 (75.5) (n=102)	53 (70.7) (n=75)	19 (86.4) (n=22)	162 (80.2) (n=202)	111 (81.0) (n=137)	35 (76.1) (n=46)
**Week 48**	77 (73.3) (n=105)	53 (68.8) (n=77)	20 (87.0) (n=23)	155 (75.2) (n=206)	110 (77.5) (n=142)	32 (69.6) (n=46)
**Week 52**	76 (72.4) (n=105)	53 (68.8) (n=77)	19 (82.6) (n=23)	148 (72.2) (n=205)	104 (74.3) (n=140)	31 (67.4) (n=46)

MAO-B, monoamine oxidase B; PIGD, postural instability gait dysfunction; REM, rapid eye movement.

Supplementary Table 6: Levodopa equivalent daily dose on the MDS-UPDRS Part III OFF assessment at all visits for placebo and pooled prasinezumab groups− For the whole population and all subpopulations including those taking MAO-B inhibitors at baseline and those who were treatment-naïve at baseline

MAO-B inhibitors at baseline						
	**Placebo**			**Prasinezumab pooled**		
**Mean (SD)**	**Whole population (n=105)**	**Treatment-naïve (n=67)**	**MAO-B inhibitors (n=38)**	**Whole population (n=211)**	**Treatment-naïve (n=134)**	**MAO-B inhibitors (n=77)**
**Baseline**	41.90 (49.09) (n=105)	11.19 (31.17) (n=67)	96.05 (17.94) (n=38)	40.17 (48.68) (n=211)	8.21 (27.55) (n=134)	95.78 (16.92) (n=77)
**Week 8**	42.31 (49.15) (n=104)	11.36 (31.37) (n=66)	96.05 (17.94) (n=38)	39.88 (48.62) (n=210)	8.21 (27.55) (n=134)	95.72 (17.02) (n=76)
**Week 16**	49.42 (69.42) (n=103)	18.46 (62.85) (n=65)	102.37 (43.46) (n=38)	44.06 (53.39) (n=202)	13.28 (42.32) (n=128)	97.30 (14.07) (n=74)
**Week 24**	69.70 (141.59) (n=101)	38.10 (155.21) (n=63)	122.11 (96.43) (n=38)	54.46 (76.99) (n=199)	23.39 (73.37) (n=124)	105.83 (51.18) (n=75)
**Week 32**	81.68 (113.04) (n=98)	48.20 (107.31) (n=61)	136.88 (101.04) (n=37)	78.20 (117.59) (n=199)	52.68 (131.28) (n=123)	119.50 (75.31) (n=76)
**Week 40**	102.20 (132.61) (n=102)	75.36 (131.05) (n=65)	149.36 (123.36) (n=37)	100.79 (149.38) (n=201)	75.34 (164.22) (n=126)	143.56 (108.65) (n=75)
**Week 48**	108.29 (136.80) (n=104)	82.43 (138.51) (n=66)	153.22 (122.97) (n=38)	118.44 (171.50) (n=203)	94.75 (190.43) (n=127)	158.04 (125.49) (n=76)
**Week 52**	121.15 (139.30) (n=99)	97.85 (144.36) (n=61)	158.57 (123.56) (n=38)	134.38 (172.98) (n=190)	114.64 (195.16) (n=114)	164.00 (128.59) (n=76)

Hoehn and Yahr stage						
	**Placebo**			**Prasinezumab pooled**		
**Mean (SD)**	**Whole population (n=105)**	**Stage 1 (n=20)**	**Stage 2 (n=85)**	**Whole population (n=211)**	**Stage 1 (n=58)**	**Stage 2 (n=153)**
**Baseline**	41.90 (49.09) (n=105)	50.00 (48.67) (n=20)	40.00 (49.28) (n=85)	40.17 (48.68) (n=211)	44.83 (49.28) (n=58)	38.40 (48.50) (n=153)
**Week 8**	42.31 (49.15) (n=104)	50.00 (48.67) (n=20)	40.48 (49.38) (n=84)	39.88 (48.62) (n=210)	44.83 (49.28) (n=58)	37.99 (48.40) (n=152)
**Week 16**	49.42 (69.42) (n=103)	50.00 (48.67) (n=20)	49.28 (73.80) (n=83)	44.06 (53.39) (n=202)	46.55 (49.44) (n=58)	43.06 (55.03) (n=144)
**Week 24**	69.70 (141.59) (n=101)	50.00 (48.67) (n=20)	74.57 (156.13) (n=81)	54.46 (76.99) (n=199)	49.09 (49.53) (n=55)	56.51 (85.24) (n=144)
**Week 32**	81.68 (113.04) (n=98)	65.23 (80.01) (n=20)	85.90 (120.12) (n=78)	78.20 (117.59) (n=199)	69.09 (99.30) (n=55)	81.68 (124.01) (n=144)
**Week 40**	102.20 (132.61) (n=102)	75.73 (117.41) (n=20)	108.66 (135.94) (n=82)	100.79 (149.38) (n=201)	92.14 (142.77) (n=56)	104.14 (152.21) (n=145)
**Week 48**	108.29 (136.80) (n=104)	78.33 (120.95) (n=20)	115.43 (140.02) (n=84)	118.44 (171.50) (n=203)	94.58 (132.09) (n=55)	127.31 (183.61) (n=148)
**Week 52**	121.15 (139.30) (n=99)	83.82 (123.16) (n=19)	130.02 (142.13) (n=80)	134.38 (172.98) (n=190)	107.85 (134.64) (n=56)	145.47 (186.02) (n=134)
REM Sleep behavior disorder score						
	**Placebo**			**Prasinezumab pooled**		
**Mean (SD)**	**Whole population**	**<5**	**≥5**	**Whole population**	**<5**	**≥5**

	(n=105)	(n=81)	(n=24)	(n=211)	(n=149)	(n=61)
**Baseline**	41.90 (49.09) (n=105)	42.59 (49.44) (n=81)	39.58 (48.85) (n=24)	40.17 (48.68) (n=211)	38.76 (48.23) (n=149)	44.26 (50.08) (n=61)
**Week 8**	42.31 (49.15) (n=104)	43.13 (49.52) (n=80)	39.58 (48.85) (n=24)	39.88 (48.62) (n=210)	38.34 (48.13) (n=148)	44.26 (50.08) (n=61)
**Week 16**	49.42 (69.42) (n=103)	52.41 (74.56) (n=79)	39.58 (48.85) (n=24)	44.06 (53.39) (n=202)	44.37 (54.93) (n=142)	43.33 (49.97) (n=60)
**Week 24**	69.70 (141.59) (n=101)	72.31 (148.71) (n=78)	60.87 (116.73) (n=23)	54.46 (76.99) (n=199)	58.54 (85.87) (n=139)	45.00 (50.17) (n=60)
**Week 32**	81.68 (113.04) (n=98)	82.66 (110.30) (n=77)	78.10 (125.40) (n=21)	78.20 (117.59) (n=199)	88.50 (132.16) (n=139)	54.33 (68.70) (n=60)
**Week 40**	102.20 (132.61) (n=102)	103.58 (133.44) (n=78)	97.71 (132.62) (n=24)	100.79 (149.38) (n=201)	112.71 (161.11) (n=143)	71.42 (111.36) (n=58)
**Week 48**	108.29 (136.80) (n=104)	110.53 (138.00) (n=81)	100.43 (135.19) (n=23)	118.44 (171.50) (n=203)	129.94 (186.61) (n=142)	91.68 (127.06) (n=61)
**Week 52**	121.15 (139.30) (n=99)	124.41 (141.33) (n=77)	109.77 (134.52) (n=22)	134.38 (172.98) (n=190)	143.45 (185.46) (n=135)	112.14 (136.62) (n=55)
Data-driven subphenotype						
	**Placebo**			**Prasinezumab pooled**		
**Mean (SD)**	**Whole population (n=105)**	**Non-diffuse malignant (n=89)**	**Diffuse malignant (n=16)**	**Whole population (n=211)**	**Non-diffuse malignant (n=167)**	**Diffuse malignant (n=44)**
**Baseline**	41.90 (49.09) (n=105)	40.45 (48.78) (n=89)	50.00 (51.64) (n=16)	40.17 (48.68) (n=211)	37.57 (47.99) (n=167)	50.00 (50.58) (n=44)

**Week 8**	42.31 (49.15) (n=104)	40.91 (48.86) (n=88)	50.00 (51.64) (n=16)	39.88 (48.62) (n=210)	37.20 (47.89) (n=166)	50.00 (50.58) (n=44)
**Week 16**	49.42 (69.42) (n=103)	49.31 (72.46) (n=87)	50.00 (51.64) (n=16)	44.06 (53.39) (n=202)	40.63 (50.22) (n=160)	57.14 (63.02) (n=42)
**Week 24**	69.70 (141.59) (n=101)	63.52 (140.93) (n=88)	111.54 (144.56) (n=13)	54.46 (76.99) (n=199)	50.63 (75.29) (n=158)	69.21 (82.54) (n=41)
**Week 32**	81.68 (113.04) (n=98)	66.38 (89.41) (n=83)	166.33 (180.70) (n=15)	78.20 (117.59) (n=199)	72.32 (115.43) (n=158)	100.85 (124.42) (n=41)
**Week 40**	102.20 (132.61) (n=102)	87.55 (118.33) (n=86)	180.94 (176.69) (n=16)	100.79 (149.38) (n=201)	91.61 (142.57) (n=159)	135.56 (170.18) (n=42)
**Week 48**	108.29 (136.80) (n=104)	93.70 (124.42) (n=88)	188.56 (174.88) (n=16)	118.44 (171.50) (n=203)	106.60 (162.17) (n=162)	165.24 (199.66) (n=41)
**Week 52**	121.15 (139.30) (n=99)	106.87 (128.37) (n=84)	201.13 (173.38) (n=15)	134.38 (172.98) (n=190)	116.54 (161.13) (n=154)	210.71 (201.70) (n=36)
Age at baseline						
	**Placebo**			**Prasinezumab pooled**		
**Mean (SD)**	**Whole population (n=105)**	**<60 years (n=50)**	**≥60 years (n=55)**	**Whole population (n=211)**	**<60 years (n=98)**	**≥60 years (n=113)**
**Baseline**	41.90 (49.09) (n=105)	47.00 (49.91) (n=50)	37.27 (48.32) (n=55)	40.17 (48.68) (n=211)	45.66 (49.62) (n=98)	35.40 (47.57) (n=113)
**Week 8**	42.31 (49.15) (n=104)	47.00 (49.91) (n=50)	37.96 (48.50) (n=54)	39.88 (48.62) (n=210)	45.66 (49.62) (n=98)	34.82 (47.38) (n=112)
**Week 16**	49.42 (69.42) (n=103)	52.86 (64.74) (n=49)	46.30 (73.88) (n=54)	44.06 (53.39) (n=202)	50.00 (52.36) (n=94)	38.89 (53.97) (n=108)

**Week 24**	69.70 (141.59) (n=101)	58.98 (67.34) (n=49)	79.81 (186.63) (n=52)	54.46 (76.99) (n=199)	62.36 (85.23) (n=92)	47.66 (68.81) (n=107)
**Week 32**	81.68 (113.04) (n=98)	74.23 (80.94) (n=46)	88.27 (135.76) (n=52)	78.20 (117.59) (n=199)	89.69 (122.37) (n=91)	68.52 (113.07) (n=108)
**Week 40**	102.20 (132.61) (n=102)	96.21 (114.47) (n=49)	107.74 (148.31) (n=53)	100.79 (149.38) (n=201)	121.04 (163.75) (n=94)	83.01 (133.78) (n=107)
**Week 48**	108.29 (136.80) (n=104)	105.24 (127.52) (n=49)	111.02 (145.68) (n=55)	118.44 (171.50) (n=203)	129.43 (168.77) (n=93)	109.15 (173.99) (n=110)
**Week 52**	121.15 (139.30) (n=99)	114.42 (129.33) (n=47)	127.24 (148.73) (n=52)	134.38 (172.98) (n=190)	149.85 (174.42) (n=89)	120.76 (171.40) (n=101)
Sex						
	**Placebo**			**Prasinezumab pooled**		
**Mean (SD)**	**Whole population (n=105)**	**Female (n=34)**	**Male (n=71)**	**Whole population (n=211)**	**Female (n=69)**	**Male (n=142)**
**Baseline**	41.90 (49.09) (n=105)	42.65 (49.44) (n=34)	41.55 (49.27) (n=71)	40.17 (48.68) (n=211)	44.20 (49.66) (n=69)	38.20 (48.26) (n=142)
**Week 8**	42.31 (49.15) (n=104)	42.65 (49.44) (n=34)	42.14 (49.37) (n=70)	39.88 (48.62) (n=210)	43.38 (49.55) (n=68)	38.20 (48.26) (n=142)
**Week 16**	49.42 (69.42) (n=103)	42.65 (49.44) (n=34)	52.75 (77.51) (n=69)	44.06 (53.39) (n=202)	44.85 (49.73) (n=68)	43.66 (55.33) (n=134)
**Week 24**	69.70 (141.59) (n=101)	57.35 (81.77) (n=34)	75.97 (164.06) (n=67)	54.46 (76.99) (n=199)	50.00 (50.00) (n=65)	56.62 (87.22) (n=134)
**Week 32**	81.68 (113.04) (n=98)	75.15 (97.34) (n=30)	84.56 (119.88) (n=68)	78.20 (117.59) (n=199)	77.15 (104.86) (n=66)	78.72 (123.80) (n=133)

**Week 40**	102.20 (132.61) (n=102)	87.34 (119.87) (n=33)	109.31 (138.56) (n=69)	100.79 (149.38) (n=201)	102.53 (152.27) (n=68)	99.91 (148.46) (n=133)
**Week 48**	108.29 (136.80) (n=104)	89.55 (121.61) (n=33)	117.01 (143.29) (n=71)	118.44 (171.50) (n=203)	112.90 (162.32) (n=69)	121.30 (176.56) (n=134)
**Week 52**	121.15 (139.30) (n=99)	94.57 (119.57) (n=33)	134.45 (147.24) (n=66)	134.38 (172.98) (n=190)	128.87 (169.42) (n=62)	137.05 (175.27) (n=128)
Disease duration						
	**Placebo**			**Prasinezumab pooled**		
**Mean (SD)**	**Whole population (n=105)**	**≤12 months (n=72)**	**>12 months (n=33)**	**Whole population (n=211)**	**≤12 months (n=147)**	**>12 months (n=64)**
**Baseline**	41.90 (49.09) (n=105)	36.11 (47.64) (n=72)	54.55 (50.56) (n=33)	40.17 (48.68) (n=211)	35.20 (47.61) (n=147)	51.56 (49.58) (n=64)
**Week 8**	42.31 (49.15) (n=104)	36.62 (47.78) (n=71)	54.55 (50.56) (n=33)	39.88 (48.62) (n=210)	34.76 (47.47) (n=146)	51.56 (49.58) (n=64)
**Week 16**	49.42 (69.42) (n=103)	43.57 (68.59) (n=70)	61.82 (70.60) (n=33)	44.06 (53.39) (n=202)	40.07 (54.52) (n=141)	53.28 (49.89) (n=61)
**Week 24**	69.70 (141.59) (n=101)	68.12 (159.96) (n=69)	73.13 (92.37) (n=32)	54.46 (76.99) (n=199)	52.19 (81.32) (n=137)	59.48 (66.79) (n=62)
**Week 32**	81.68 (113.04) (n=98)	72.31 (111.40) (n=67)	101.94 (115.74) (n=31)	78.20 (117.59) (n=199)	78.96 (127.77) (n=140)	76.39 (89.85) (n=59)
**Week 40**	102.20 (132.61) (n=102)	96.51 (136.27) (n=71)	115.25 (124.99) (n=31)	100.79 (149.38) (n=201)	103.46 (163.17) (n=141)	94.53 (111.39) (n=60)
**Week 48**	108.29 (136.80) (n=104)	99.99 (137.39) (n=71)	126.17 (135.87) (n=33)	118.44 (171.50) (n=203)	116.19 (179.28) (n=143)	123.83 (152.62) (n=60)

**Week 52**	121.15 (139.30) (n=99)	111.15 (140.63) (n=69)	144.17 (135.69) (n=30)	134.38 (172.98) (n=190)	136.40 (186.63) (n=132)	129.80 (138.32) (n=58)
Age at diagnosis						
	**Placebo**			**Prasinezumab pooled**		
**Mean (SD)**	**Whole population (n=105)**	**<60 years (n=52)**	**≥60 years (n=53)**	**Whole population (n=211)**	**<60 years (n=102)**	**≥60 years (n=109)**
**Baseline**	41.90 (49.09) (n=105)	47.12 (49.92) (n=52)	36.79 (48.19) (n=53)	40.17 (48.68) (n=211)	45.83 (49.64) (n=102)	34.86 (47.39) (n=109)
**Week 8**	42.31 (49.15) (n=104)	47.12 (49.92) (n=52)	37.50 (48.38) (n=52)	39.88 (48.62) (n=210)	45.30 (49.59) (n=101)	34.86 (47.39) (n=109)
**Week 16**	49.42 (69.42) (n=103)	52.75 (64.22) (n=51)	46.15 (74.65) (n=52)	44.06 (53.39) (n=202)	50.00 (52.27) (n=98)	38.46 (54.07) (n=104)
**Week 24**	69.70 (141.59) (n=101)	58.63 (66.75) (n=51)	81.00 (190.03) (n=50)	54.46 (76.99) (n=199)	61.85 (84.09) (n=96)	47.57 (69.44) (n=103)
**Week 32**	81.68 (113.04) (n=98)	74.78 (80.14) (n=47)	88.04 (137.10) (n=51)	78.20 (117.59) (n=199)	88.02 (120.45) (n=95)	69.23 (114.76) (n=104)
**Week 40**	102.20 (132.61) (n=102)	95.42 (112.33) (n=51)	108.98 (151.04) (n=51)	100.79 (149.38) (n=201)	119.36 (161.66) (n=97)	83.48 (135.45) (n=104)
**Week 48**	108.29 (136.80) (n=104)	104.60 (125.00) (n=51)	111.85 (148.37) (n=53)	118.44 (171.50) (n=203)	133.89 (177.43) (n=97)	104.31 (165.46) (n=106)
**Week 52**	121.15 (139.30) (n=99)	113.38 (126.73) (n=49)	128.77 (151.51) (n=50)	134.38 (172.98) (n=190)	150.39 (174.29) (n=93)	119.03 (171.19) (n=97)
Motor subphenotypes akinetic-rigid and tremor dominant						

Mean (SD)	Placebo			Prasinezumab pooled		
	**Whole population (n=105)**	**Tremor dominant (n=41)**	**Akinetic-rigid (n=52)**	**Whole population (n=211)**	**Tremor dominant (n=81)**	**Akinetic-rigid (n=113)**
**Baseline**	41.90 (49.09) (n=105)	37.80 (48.45) (n=41)	47.12 (49.92) (n=52)	40.17 (48.68) (n=211)	39.51 (48.55) (n=81)	42.26 (49.22) (n=113)
**Week 8**	42.31 (49.15) (n=104)	38.75 (48.68) (n=40)	47.12 (49.92) (n=52)	39.88 (48.62) (n=210)	38.75 (48.38) (n=80)	42.26 (49.22) (n=113)
**Week 16**	49.42 (69.42) (n=103)	54.63 (91.95) (n=41)	49.00 (49.99) (n=50)	44.06 (53.39) (n=202)	43.04 (51.72) (n=79)	46.30 (55.41) (n=108)
**Week 24**	69.70 (141.59) (n=101)	91.00 (208.74) (n=40)	60.00 (70.71) (n=50)	54.46 (76.99) (n=199)	44.74 (52.01) (n=76)	61.46 (89.70) (n=108)
**Week 32**	81.68 (113.04) (n=98)	87.69 (121.09) (n=37)	84.08 (115.20) (n=49)	78.20 (117.59) (n=199)	72.91 (114.39) (n=79)	84.78 (124.40) (n=105)
**Week 40**	102.20 (132.61) (n=102)	118.24 (146.06) (n=39)	91.48 (118.63) (n=51)	100.79 (149.38) (n=201)	96.83 (166.34) (n=78)	106.41 (142.65) (n=108)
**Week 48**	108.29 (136.80) (n=104)	123.99 (145.71) (n=40)	94.92 (119.36) (n=52)	118.44 (171.50) (n=203)	112.06 (188.09) (n=79)	126.49 (166.67) (n=108)
**Week 52**	121.15 (139.30) (n=99)	143.15 (146.79) (n=38)	104.62 (123.30) (n=49)	134.38 (172.98) (n=190)	126.25 (182.63) (n=72)	146.20 (173.87) (n=102)
Motor subphenotypes PIGD and tremor dominant						
	**Placebo**			**Prasinezumab pooled**		
**Mean (SD)**	**Whole population (n=105)**	**Tremor dominant (n=77)**	**PIGD (n=23)**	**Whole population (n=211)**	**Tremor dominant (n=144)**	**PIGD (n=47)**

**Baseline**	41.90 (49.09) (n=105)	42.21 (49.38) (n=77)	36.96 (48.19) (n=23)	40.17 (48.68) (n=211)	39.58 (48.72) (n=144)	43.62 (49.58) (n=47)
**Week 8**	42.31 (49.15) (n=104)	42.76 (49.47) (n=76)	36.96 (48.19) (n=23)	39.88 (48.62) (n=210)	39.16 (48.62) (n=143)	43.62 (49.58) (n=47)
**Week 16**	49.42 (69.42) (n=103)	51.84 (75.40) (n=76)	38.64 (48.63) (n=22)	44.06 (53.39) (n=202)	42.75 (50.74) (n=138)	50.00 (62.36) (n=46)
**Week 24**	69.70 (141.59) (n=101)	77.57 (161.98) (n=74)	45.45 (53.25) (n=22)	54.46 (76.99) (n=199)	47.69 (55.78) (n=135)	66.67 (95.35) (n=45)
**Week 32**	81.68 (113.04) (n=98)	85.98 (111.71) (n=71)	72.73 (128.85) (n=22)	78.20 (117.59) (n=199)	70.69 (102.17) (n=138)	101.95 (141.67) (n=42)
**Week 40**	102.20 (132.61) (n=102)	111.14 (135.61) (n=74)	82.61 (134.51) (n=23)	100.79 (149.38) (n=201)	92.69 (145.42) (n=139)	122.77 (148.69) (n=44)
**Week 48**	108.29 (136.80) (n=104)	118.22 (140.39) (n=76)	82.61 (134.51) (n=23)	118.44 (171.50) (n=203)	109.03 (171.88) (n=139)	142.92 (160.33) (n=46)
**Week 52**	121.15 (139.30) (n=99)	132.37 (142.05) (n=73)	87.55 (136.67) (n=22)	134.38 (172.98) (n=190)	123.73 (171.75) (n=132)	163.94 (166.64) (n=42)

MAO-B, monoamine oxidase B; PIGD, postural instability gait dysfunction; REM, rapid eye movement.

Supplementary Table 7: Percentage of patients in a specific fast-progressing subgroup who also belong to other fast-progressing subgroups

	MAO-B inhibitors at baseline (n=115)	***Hoehn and Yahr stage 2 (n=238)***	***REM sleep behavior disorder score ≥5 (n=85)***	***Data-driven subphenotype: diffuse malignant (n=59)***	***Age at baseline ≥60 years (n=168)***	***Male gender (n=213)***	***Disease duration >12 months (n=97)***	***Age at diagnosis ≥60 years (n=162)***	***Motor subphenotype akinetic-rigid (n=165)***	***Motor subphenotype PIGD (n=70)***
MAO-B inhibitors at baseline, n (%)	115 (100)	83 (72.2)	33 (28.7)	24 (20.9)	53 (46.1)	74 (64.3)	46 (40.0)	49 (42.6)	64 (55.7)	29 (25.2)
Hoehn and Yahr stage 2, n (%)	83 (34.9)	238 (100)	66 (27.7)	50 (21.0)	131 (55.0)	166 (69.7)	74 (31.1)	125 (52.5)	133 (55.9)	51 (21.4)
REM Sleep behavior disorder score ≥5, n (%)	33 (38.8)	66 (77.6)	85 (100)	32 (37.6)	57 (67.1)	56 (65.9)	23 (27.1)	57 (67.1)	41 (48.2)	19 (22.4)
Data-driven subphenotype: diffuse malignant, n (%)	24 (40.7)	50 (84.7)	32 (54.2)	59 (100)	40 (67.8)	39 (66.1)	11 (18.6)	40 (67.8)	30 (50.8)	14 (23.7)
Age at baseline ≥60 years, n (%)	53 (31.5)	131 (78.0)	57 (33.9)	40 (23.8)	168 (100)	113 (67.3)	49 (29.2)	162 (96.4)	83 (49.4)	31 (18.5)
Male gender, n (%)	74 (34.7)	166 (77.9)	56 (26.3)	39 (18.3)	113 (53.1)	213 (100)	67 (31.5)	109 (51.2)	114 (53.5)	49 (23.0)
Disease duration >12 months, n (%)	46 (47.4)	74 (76.3)	23 (23.7)	11 (11.3)	49 (50.5)	67 (69.1)	97 (100)	44 (45.4)	43 (44.3)	21 (21.6)
Age at diagnosis ≥60 years, n (%)	49 (30.2)	125 (77.2)	57 (35.2)	40 (24.7)	162 (100)	109 (67.3)	44 (27.2)	162 (100)	81 (50.0)	30 (18.5)
Motor subphenotype akinetic-rigid, n (%)	64 (38.8)	133 (80.6)	41 (24.8)	30 (18.2)	83 (50.3)	114 (69.1)	43 (26.1)	81 (49.1)	165 (100)	70 (42.4)

Motor subphenotype PIGD, n (%)	29 (41.4)	51 (72.9)	19 (27.1)	14 (20.0)	31 (44.3)	49 (70.0)	21 (30.0)	30 (42.9)	70 (100)	70 (100)

MAO-B, monoamine oxidase B; PIGD, postural instability gait dysfunction; REM, rapid eye movement.

Supplementary Fig. 1: Forest plot of prasinezumab effects on motor progression as measured by the MDS-UPDRS Part III (hypothetical strategy) across the pre-specified exploratory subpopulations. Adj., adjusted; CI, confidence interval; PIGD, postural instability gait dysfunction.

Category	Subgroup	Total n	Placebo adj. mean	Prasinezumab pool adj. mean	Adj. mean difference	80% CI	Relative difference
Age at baseline	≥60 years	168	7.04	5.14	-1.89	(-3.90, 0.12)	-26.9%
	<60 years	148	3.83	3.19	-0.61	(-2.58, 1.36)	-15.9%
Sex	Male	213	6.18	4.95	-1.23	(-2.89, 0.44)	-19.9%
	Female	103	4.20	2.94	-1.22	(-3.83, 1.39)	-29.1%
Disease duration	>12 months	97	5.98	4.96	-1.12	(-3.91, 1.68)	-18.7%
	<12 months	219	5.60	3.83	-1.78	(-3.45, -0.10)	-31.8%
Age at diagnosis	≥60 years	162	7.18	5.14	-2.03	(-4.08, 0.02)	-28.3%
	<60 years	154	3.74	3.19	-0.52	(-2.44, 1.41)	-13.9%
Motor subphenotype	Akinetic-rigid	165	6.00	3.39	-2.61	(-4.48, -0.74)	-43.5%
	Tremor dominant	122	4.93	4.87	-0.05	(-2.19, 2.10)	-1.0%
	PIGD	70	8.40	3.43	-4.96	(-7.44, -2.48)	-59.1%
	Tremor dominant	221	4.70	4.25	-0.44	(-2.17, 1.30)	-9.4%

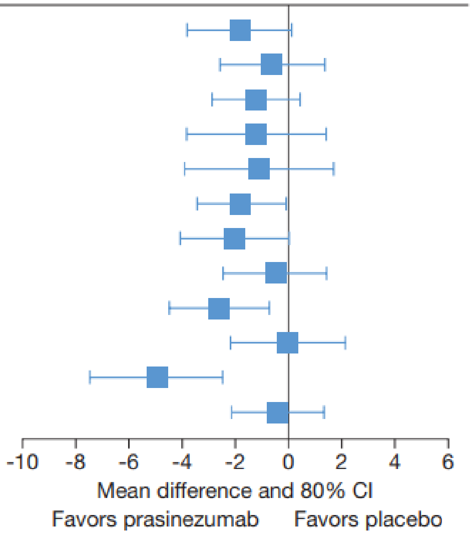

A full list of the members of the PASADENA Investigators and Prasinezumab Study Group

PASADENA Investigators

Claudia Altendorf[1], Chareyna Anandan[2], Giulia Andrews[3], Solène Ansquer[4], Raphaele Arrouasse[5], Sana Aslam[3], Jean-Philippe Azula[6], Jeanette Baker[7], Ernest Balaguer Martinez[8], Shadi Barbu[3], Kara Bardram[9], Danny Bega[10], Helena Bejr-Kasem Marco[11], Isabelle Benatru[4], Eve Benchetrit[6], Felix Bernhard[12], Amit Besharat[13], Sagari Bette[14], Amelie Bichon[15], Andrew Billnitzer[2], Sophie Blondeau[4], Thomas Boraud[16], Freiderike Borngräber[1], James Boyd[7], Kathrin Brockmann[17], Matthew Brodsky[18], Ethan Brown[19], Christof Bruecke[1], Fabienne Calvas[20], Monica Canelo[21], Federico Carbone[22], Claire Carroll[23], Laura Casado Fernandez[24], Catherine Casse-Parrot[6], Anna Castrioto[15], Helene Catala[20], Justine Chan[3], Samia Cheriet[20], Anthony Ciabarra[25], Joseph Classen[26], Juliana Coleman[27], Robert Coleman[27], Yaroslau Compta[28], Jean-Christophe Corvol[29], Mariana Cosgaya[28], Nabila Dahodwala[30], Philippe Damier[31], Elodie David[32], Thomas Davis[33], Marissa Dean[34], Berengere Debilly[35], Janell DeGiorgio[36], Andres Deik[30], Laure Delaby[35], Marie-Helene Delfini[6], Pascal Derkinderen[31], Philipp Derost[35], Maria de Toledo[24], Lisa Deuel[7], Ann Marie DiazHernandez[14], Cameron Dietiker[19], Karina Dimenshteyn[37], Julio Dotor[24], Franck Durif[35], Jens Ebentheuer[21], Karla Maria Eggert[38], Sara Eichau Madueño[39], Claudia Eickoff[37], Aaron Ellenbogen[9], Philipp Ellmerer[22], Ines Esparragosa Vazquez[40], Alexandre Eusebio[6], Siobhan Ewert[1], John Fang[33], Danielle Feigenbaum[13], Frederique Fluchere[6], Alexandra Foubert-Samier[16], Marie Fournier[15], Anne Fradet[4], Valerie Fraix[15], Samuel Frank[41], Franka Fries[17], Monique Gailitzky[20], Anne Gaille Corbille[31], Marisol Gallardó Pérez[42], Jose Manuel Garcia Morena[39], Carmen Gasca[43], Thomas Gasser[17], Joyce Gibbons[44], Caroline Giordana[32], Alicia Gonzalez Martinez[24], Ira Goodman[23], Arantza Gorospe[40], Marie Goubeaud[20], David Grabli[29], Mangone Graziella[29], Stephan Grimaldi[6], Jeffrey Gross[45], Raquel Guimaraes-Costa[29], Andreas Hartmann[29], Christian Hartmann[37], Travis Hassell[33], Robert Hauser[46], Antonio Hernandez[8], Jorge Hernandez-Vara[47], Günter Höglinger[48], Christian Homedes[8], Andrea Horta[11],

Jean-Luc Houeto[4], Julius Huebl[1], Jennifer Hui[13], Stuart Isaacson[14], Joseph Jankovic[2], Annette Janzen[38], Jocelyne Jiao[18], Maria Jose Marti Domenech[49], Xavier Joseph[5], Srinath Kadimi[45], Pat Kaminski[50], Silja Kannenberg[37], R. Jan Kassubek[51], Maya Katz[19], Kevin Klos[52], Shannon Klos[52], Christopher Kobet[27], Jennifer Koebert[46], Patricia Krause[1], Andrea Kuhn[1], Jaime Kulisevsky Bojarsky[53], Rajeev Kumar[36], Martin Kunz[52], Lille Kurvits[1], Kimberly Kwei[54], Simon Laganiere[41], Brice Laurens[16], Johannes Levin[48], Oren Levy[55], Peter Le Witt[51], Gurutz Linazasoro Cristobal[11], Irene Litvan[55], Karlo Lizarraga[56], Katherine Longardner[57], Rocio Lopez[39], Lydia Lopez Manzanares[24], Sara Lucas del Pozo[47], Maria Rosario Luquin Puido[40], Nijee Luthra[19], Kelly Lyons[57], Sylvia Maass[48], Gerrit Machetanz[17], Yolanda Macias[43], David Maltete[58], Jorge Uriel Manez Miro[43], Louis-Laure Mariani[29], Juan Marin[11], Kathrin Marini[22], Ana Marques[35], Gloria Marti[40], Saul Martinez[11], Wassilios Meissner[16], Sara Meoni[15], Brit Mollenhauer[21], Dunia Mon Martinez[8], Johnson Moon[25], Elena Moro[15], Peter Morrison[56], Christoph Muehlberg[26], Manpreet Multani[25], Christine Murphy[23], Anthony Nicholas[34], Rajesh Pahwa[57], Antonio Palasis[47], Heidi Pape[38], Neepa Patel[50], Prity Patel[23], Marina Peball[22], Elizabeth Peckham[59], Terry Peery[59], Jesus Perez[11], Rafael Perez Alisa Petit[39], Elmar Pinkhardt[51], Werner Poewe[22], Elsa Pomies[20], Cecile Preterre[31], Joseph Quinn[18], Olivier Rascol[20], Philippe Remy[5], Emily Reuther[27], Irene Richard[56], Benjamin Roeben[17], Jost-Julian Rumpf[26], David Russell[44], Hayet Salhi[5], Daniela Samaniego[47], Alexandra Samier-Foubert[16], Alvaro Sanchez-Ferro[43], Emmanuelle Schmitt[15], Alfons Schnitzler[37], Oliver Schorr[22], Julie Schwartzbard[60], Kerstin Schweyer[48], Klaus Seppi[22], Victoria Sergo[36], Holly Shill[3], Andrew Siderowf[30], Tanya Simuni[10], Umberto Spampinato[16], Ashok Sriram[27], Natividad Stover[34], Caroline Tanner[19], Arjun Tarakad[2], Carolyn Taylor[10], Claire Thalamus[20], Thomas Toothaker[45], Nadege Van Blercom[11], Nora Vanegas-Arrogave[54], Lydia Vela[43], Sylvian Vergnet[16], Tiphaine Vidal[35], Jonathan Vöglein[48], Ryan Walsh[3], Cheryl Waters[54], Mirko Wegschneider[26], Endy Weidinger[26], Caroline Weill[5], Gregor Wenzel[1], Tatiana Witjas[6], Isabel Wurster[17], Brenton Wright[55], Milan Zimmermann[17], Rafael Zuzuarregui[19]

1. Berlin Medical University, Neurology Clinic, Campus Charité Mitte, Berlin, Germany;

2. Baylor College of Medicine, Houston, TX, USA;

3. Barrow Neurological Institute, Phoenix, AZ, USA;

4. Poitiers University Hospital, Poitiers, France;

5. Henri-Mondor University Hospital, Créteil, France;

6. Marseille University Hospital Timone, Marseille, France;

7. University of Vermont, Larner College of Medicine, Burlington, VT, USA;

8. General University Hospital of Catalonia, Barcelona, Spain;

9. Quest Research Institute, Farmington Hills, MI, USA;

10. Northwestern University, Evanston, IL, USA;

11. Policlinica Gipuzkoa Servicio De Neurologia, Gipuzkoa, Spain;

12. Philipps University of Marburg, Neurology Clinic, Marburg, Germany;

13. University of Southern California, Keck Medical Center, Los Angeles, CA, USA;

14. Parkinson's Disease and Movement Disorders Center of Boca Raton, Boca Raton, FL, USA;

15. Grenoble Alpes University Michallon Hospital, La Tronche, France;

16. Hospital Pellegrin Bordeaux, Bordeaux, France;

17. Tubingen University Hospital, Tubingen, Germany;

18. Oregon Health & Science University, Portland, OR, USA;

19. University of California, San Francisco, CA, USA;

20. Toulouse University, Clinical Research Center, Purpan Hospital, Toulouse, France;

21. Goettingen University Medical Center, Paracelsus Elena Klinik Kassel, Goettingen, Germany;

22. Medical University of Innsbruck, Neuroradiology Clinic, Innsbruck, Austria;

23. Compass Research LLC, Orlando, FL, USA;

24. De La Princessa University Hospital, Madrid, Spain;

25. Neurology Center of North Orange County, Fullerton, CA, USA;

26. Leipzig University, Neurology Clinic and Polyclinic, Leipzig, Germany;

27. Spectrum Health Medical Group, USA;

28. Hospital Clinic Barcelona, Barcelona, Spain;

29. Sorbonne University, Pitié-Salpêtrière University Hospital, Paris, France;

30. University of Pennsylvania, Philadelphia, PA, USA;

31. Nantes University, North Laennec University Hospital, Saint-Herblain, France;

32. Nice University, Hospital Pasteur, Nice, France;

33. Vanderbilt University Medical Center, Nashville, TN, USA;

34. University of Alabama, UAB Medicine, Birmingham, AL, USA;

35. Clermont-Ferrand University Hospital Center, Site Gabriel-Montpied, Clermont-Ferrand, France;

36. Rocky Mountain Movement Disorders Center, Englewood, CO, USA;

37. Heinrich Heine University Düsseldorf, University Hospital Düsseldorf, Germany;

38. Philips University of Marburg, Marburg, Germany;

39. University of Sevilla, Virgin Macarena University Hospital, Sevilla, Spain;

40. University of Navarra, Navarra University Hospital, Department of Neurology, Navarre, Spain;

41. Beth Israel Deaconess Medical Center, Boston, MA, USA;

42. University of Barcelona, The Provincial Clinic Hospital, Barcelona, Spain;

43. University of Madrid, The Alcorcón Foundation University Hospital, Madrid, Spain;

44. Invicro, New Haven, CT, USA;

45. Associated Neurologists of Southern Connecticut, P.C., Milford, CT, USA;

46. University of South Florida, Parkinson’s Disease and Movement Disorders, Tampa, FL, USA;

47. Vall d'Hebron Barcelona University Hospital, Department of Neurology, Barcelona, Spain;

48. Technical University of Munich, The Rechts der Isar Hospital, Munich, Germany;

49. Neurosciences Clinic Institute of the Barcelona Hospital Clinic, Parkinson Disease and Movement Disorders Unit, Barcelona, Spain;

50. Henry Ford Health System. Clarkston, MI, USA;

51. University of Ulm, Ulm University Hospital, Clinic for Neurology, Ulm, Germany;

52. The Movement Disorder Clinic of Oklahoma, Tulsa, OK, USA;

53. Autonomous University of Barcelona, Hospital de la Santa Creu i Sant Pau, Autoimmune Neurology Unit – Neurology Service, Barcelona, Spain;

54. Columbia University, New York, CU, USA;

55. University of California San Diego Altman Clinical and Translational Research Institute, La Jolla, CA, USA;

56. University of Rochester Medical Center, Rochester, NY, USA;

57. University of Kansas Medical Center, Kansas City, KS, USA;

58. University of Rouen Normandy Hospital, Charles Nicolle Parkinson's Disease Center, Rouen, France;

59. Central Texas Neurology Consultants, Round Rock, TX, USA;

60. Aventura Neurologic Associates, Aventura, FL, USA;

Prasinezumab Study Group

Markus Abt,[1] Atieh Bamdadian,[1] Teresa Barata,[1] Nicholas Barbet,[1] Sara Belli,[1] Frank Boess,[1] Azad Bonni,[1] Edilio Borroni,[1] Anne Boulay,[1] Markus Britschgi,[1] Valerie Cosson,[1] Christian Czech,[1] Evan Davies,[1] Dennis Deptula,[1] Cheikh Diack,[1] Rachelle Doody,[1] Juergen Dukart,[1] Giulia D'Urso,[1] Sebastian Dziadek,[1] Chris Edgar,[1] Laurent Essioux,[1] Morgan Farell,[1] Rebecca Finch,[1] Paulo Fontoura,[1] Waltraud Gruenbauer,[1] Andrea Hahn,[1] Stefan Holiga,[1] Michael Honer,[1] Shirin Jadidi,[1] Timothy Kilchenmann,[1] Thomas Kremer,[1] Thomas Kustermann,[1] Claire Landsdall,[1] Michael Lindemann,[1] Florian Lipsmeier,[1] Cecile Luzy,[1] Marianne Manchester,[1] Maddalena Marchesi,[1] Ferenc Martenyi,[2] Meret Martin-Facklam,[1] Katerina Mironova,[1] Emma

Moore,[1] Annabelle Monnet,[1] Markus Niggli,[1] Tania Nikolcheva,[2] Susanne Ostrowitzki,[1] Gennaro Pagano,[1] Benedicte Passemard,[1] Agnes Poirier,[1] Anke Post,[1] Megana Prasad,[1] Nathalie Pross,[1] Benedicte Ricci,[1] Ellen Rose,[2] Daria Rukina,[1] Christoph Sarry,[1] Marzia A. Scelsi,[1] Christine Schubert,[1] Jeff Sevigny,[1] Kaycee Sink,[1] Nima Shariati,[1] Alexander Strasak,[1] Hannah Staunton,[1] Hanno Svoboda,[1] Kirsten I. Taylor,[1] Dylan Trundell,[1] Daniel Umbricht,[1] Lynne Verselis,[1] Annamarie Vogt,[1] Ekaterina Volkova-Volkmar,[1] Cornelia Weber,[1] Silke Weber,[1] Stefano Zanigni[1]

1. F. Hoffmann-La Roche Ltd, Basel, Switzerland;

2. Prothena Corporation plc, Dublin, Ireland
