# Supplementary material for: Prasinezumab slows motor progression in rapidly progressing early-stage Parkinson’s disease
Source: Nat Med. 2024 Apr 15;30(4):1096–103. doi: 10.1038/s41591-024-02886-y (PMC11031390; doi:10.1038/s41591-024-02886-y)
Supplement: Supplementary file 2 — Reporting Summary [file 41591_2024_2886_MOESM2_ESM.pdf]

nature portfolio

Corresponding author(s): Gennaro Pagano

Last updated by author(s): Feb 12, 2024

Reporting Summary

Nature Portfolio wishes to improve the reproducibility of the work that we publish. This form provides structure for consistency and transparency in reporting. For further information on Nature Portfolio policies, see our Editorial Policies and the Editorial Policy Checklist.

Statistics

For all statistical analyses, confirm that the following items are present in the figure legend, table legend, main text, or Methods section.

n/a	Confirmed	
☐	☒	The exact sample size (*n*) for each experimental group/condition, given as a discrete number and unit of measurement
☐	☒	A statement on whether measurements were taken from distinct samples or whether the same sample was measured repeatedly
☒	☐	The statistical test(s) used AND whether they are one- or two-sided<br>*Only common tests should be described solely by name; describe more complex techniques in the Methods section.*
☐	☒	A description of all covariates tested
☒	☐	A description of any assumptions or corrections, such as tests of normality and adjustment for multiple comparisons
☐	☒	A full description of the statistical parameters including central tendency (e.g. means) or other basic estimates (e.g. regression coefficient) AND variation (e.g. standard deviation) or associated estimates of uncertainty (e.g. confidence intervals)
☒	☐	For null hypothesis testing, the test statistic (e.g. *F*, *t*, *r*) with confidence intervals, effect sizes, degrees of freedom and *P* value noted<br>*Give P values as exact values whenever suitable.*
☒	☐	For Bayesian analysis, information on the choice of priors and Markov chain Monte Carlo settings
☒	☐	For hierarchical and complex designs, identification of the appropriate level for tests and full reporting of outcomes
☐	☒	Estimates of effect sizes (e.g. Cohen's *d*, Pearson's *r*), indicating how they were calculated

Our web collection on statistics for biologists contains articles on many of the points above.

Software and code

Policy information about availability of computer code

Data collection	Data were collected using the eCRF Medidata Classic Rave® 2021.1.2 (Copyright © 1999-2021 Medidata Solutions, Inc.). The MDS-UPDRS data were collected using Virgil Investigative Study Platform provided by MedAvante, Inc. (Copyright © 2023 WCG Clinical)
Data analysis	Data was analyzed using SAS software version 9.04 and R version 4.0.3

For manuscripts utilizing custom algorithms or software that are central to the research but not yet described in published literature, software must be made available to editors and reviewers. We strongly encourage code deposition in a community repository (e.g. GitHub). See the Nature Portfolio guidelines for submitting code & software for further information.

Data

Policy information about availability of data

All manuscripts must include a data availability statement. This statement should provide the following information, where applicable:

- Accession codes, unique identifiers, or web links for publicly available datasets
- A description of any restrictions on data availability
- For clinical datasets or third party data, please ensure that the statement adheres to our policy

Qualified researchers may request access to individual patient-level data through the clinical study data request platform (https://vivli.org/). Further details on Roche's criteria for eligible studies are available here (https://vivli.org/members/ourmembers/). For further details on Roche's Global Policy on the Sharing of

Clinical Information and how to request access to related clinical study documents, see here: (https://www.roche.com/research_and_development/who_we_are_how_we_work/clinical_trials/our_commitment_to_data_sharing.htm).

Human research participants

Policy information about studies involving human research participants and Sex and Gender in Research.

Reporting on sex and gender	Sex (male vs female) was considered in the study design as a stratification factor. Sex was self reported by the participants. The majority of trial participants were male (213/316; 67.4%). With more men than women diagnosed with Parkinson's Disease by a ratio approximately of 2:1, the proportion of male to female participants recruited in this trial is appropriate. Gender was not collected.
Population characteristics	Covariate-relevant population characteristics of the participants included: treatment (placebo/ prasinezumab), age at baseline (>60 / <60), sex (male/female), MAO-B inhibitor (yes/no) and the DaT-SPECT contralateral binding ratio at baseline (very abnormal/abnormal).
Recruitment	Participants were identified for potential recruitment per site-specific recruitment plans prior to consenting to take part in this study. Any recruitment materials for participants received Institutional Review Board or Ethics Committee (IRB/EC) approval prior to use.
Ethics oversight	The trial was conducted according to the principles of the Declaration of Helsinki and Good Clinical Practice guidelines and was approved by central institutional review boards (Ethikkommission der Medizinischen Universität Innnsbruck [Austria], Comité de Protection des Personnes [CPP] Ouest IV [France], Ethikkommission der LÄK Hessen [Germany], CEIm Hospital Universitari Vall d'Hebron [Spain], Copernicus Group Independent Review Board [US] and Western Institutional Review Board [US]) or ethics committees at each trial site (Ethikkommission der Universität Leipzig Geschäftsstelle der Ethikkommission an der medizinischen Fakultät der Universität Leipzig [Germany], Ethikkommission der Fakultät für Medizin der Technischen Universität München [Germany], Ethikkommission der Universität Ulm, (Oberer Eselsberg) [Germany], Landesamt für Gesundheit und Soziales Berlin Geschäftsstelle der Ethik-Kommission des Landes Berlin [Germany], Ethikkommission des FB Medizin der Philipps-Universität Marburg [Germany], Ethikkommission an der Medizinischen Fakultät der Eberhard-Karls-Universität und am Universitätsklinikum Tübingen [Germany], Ethikkommission an der Med. Fakultät der HHU Düsseldorf [Germany], The University of Kansas Medical Center Human Research Protection Program [US], Oregon Health & Science University Independent Review Board [US], Northwestern University Institutional Review Board [US], Spectrum Health Human Research Protection Program [US], The University of Vermont Committees on Human Subjects [US], Beth Israel Deaconess Medical Center Committee on Clinical Investigations, New Procedures and New Forms of Therapy [US], Vanderbilt Human Research Protection Program Health [US], University of Maryland, Baltimore Institutional Review Board [US], University of Southern California Institutional Review Board [US], Columbia University Medical Center Institutional Review Board [US], University of Southern California San Francisco Institutional Review Board [US], University of Pennsylvania Institutional Review Board [US] and HCA - HealthOne Institutional Review Board [US]).

Note that full information on the approval of the study protocol must also be provided in the manuscript.

Field-specific reporting

Please select the one below that is the best fit for your research. If you are not sure, read the appropriate sections before making your selection.

☒ Life sciences ☐ Behavioural & social sciences ☐ Ecological, evolutionary & environmental sciences

For a reference copy of the document with all sections, see nature.com/documents/nr-reporting-summary-flat.pdf

Life sciences study design

All studies must disclose on these points even when the disclosure is negative.

Sample size	The sample size calculations were aimed to power the study for the primary endpoint of change in total MDS-UPDRS (sum of Parts I, II and III) at Week 52. A sample size of 100 randomized participants (300 participants for the three groups) was chosen to obtain a power of ~80% at 2-sided a-level of 20% for the comparison of each active dose arm to placebo.
Data exclusions	A hypothetical estimand strategy was followed, i.e. assessments performed while on any symptomatic treatment started after randomization were not included in the analysis. However the correlation structure used in the MMRM model used for analysis allows data from subjects who were excluded to make a contribution to estimation of the effects at the final time point. For endpoints defined in terms of change from baseline, patients who did not have a pretreatment value reported for a particular assessment (if any) were excluded from the change from baseline analyses for that assessment
Replication	NA. As we report trial data, the only feasible way to replicate the results would be to run a new trial
Randomization	Randomization was done via an Interactive Voice/Web Response System (IxRS). Both randomization lists were generated by the IxRS provider. Patients were randomized once all screening assessments had been completed and eligibility confirmed. Randomization was stratified by sex, age group (<60 years vs >= 60 years), and prior background therapy with MAO-B inhibitor at randomization (Yes vs No).

Blinding	PASADENA was a double-blind study

Reporting for specific materials, systems and methods

We require information from authors about some types of materials, experimental systems and methods used in many studies. Here, indicate whether each material, system or method listed is relevant to your study. If you are not sure if a list item applies to your research, read the appropriate section before selecting a response.

Materials & experimental systems

n/a	Involved in the study	
☐	☒	Antibodies
☒	☐	Eukaryotic cell lines
☒	☐	Palaeontology and archaeology
☒	☐	Animals and other organisms
☐	☒	Clinical data
☒	☐	Dual use research of concern

Methods

n/a	Involved in the study	
☒	☐	ChIP-seq
☒	☐	Flow cytometry
☒	☐	MRI-based neuroimaging

Antibodies

Antibodies used	Prasinezumab (PRX002) is an immunoglobulin class G1 (IgG1) humanized monoclonal antibody (mAb) directed against an epitope in the C-terminus of human alpha-synuclein.
Validation	The initial step in the development of prasinezumab was to generate a murine antibody with the appropriate characteristics through standard hybridoma techniques in the mouse. In this study (1075169) the murine parent of prasinezumab, antibody 9E4, was generated by the active immunization of mice with the full-length non-mutated human alpha-synuclein. Biochemical analysis demonstrated that 9E4 bound to monomeric human alpha-synuclein with nanomolar affinity (KD approximately 220-35 nM) and high specificity under the in vitro conditions tested. Importantly, 9E4 lacked cross-reactivity with beta- and gamma-synucleins. 9E4 bound to a region of alpha-synuclein encompassing amino acids 118126 and bound with much lower affinity to murine alpha-synuclein (KD >1 uM).<br>Subsequently, humanized variants of the murine 9E4 mAb were produced in order to identify a candidate for clinical development (1075170). Sequences of variable heavy and light chains generated from hybridoma cells and expressed as mouse/human chimeric antibody showed equivalent activity towards alpha-synuclein as the 9E4 antibody. The binding affinity of the selected humanized variant (VH3L3), an IgG1 antibody, was maintained throughout the humanization process, and this feature was a primary factor guiding selection of the final humanized construct. In addition to verifying the fidelity of binding affinity by surface plasmon resonance (SPR), the humanized versions were also tested against human PD tissue by immunoprecipitation and immunohistochemistry. Consequently, as summarized in Table 4, prasinezumab binding affinity to human alpha-synuclein was similar to murine parent 9E4, was comparable between human and cynomolgus monkey alpha-synuclein and, like 9E4, was not measurable against murine alpha-synuclein (KD >3 uM). Prasinezumab binding to rat and rabbit alpha-synuclein was not detected either. Moreover, prasinezumab, as with 9E4, immunoreacted with Lewy bodies and Lewy neurites in human brains and immunoprecipitated alpha-synuclein from human brain extracts.

Clinical data

Policy information about clinical studies

All manuscripts should comply with the ICMJE guidelines for publication of clinical research and a completed CONSORT checklist must be included with all submissions.

Clinical trial registration	PASADENA Phase II study, ClinicalTrials.gov identifier: NCT03100149
Study protocol	https://www.nejm.org/doi/suppl/10.1056/NEJMoa2202867/suppl_file/nejmoa2202867_protocol.pdf
Data collection	The analysis of Part 1 took place once all the randomized patients in the study reached the Week 52 visit or withdrawn from the study prior to Week 52; and all data from the study were in the database. The patient data were cut at the day prior to the Week 56 visit. All data prior to this cut-off were cleaned and verified for completeness to the best extent possible. The following data was included in the 52-week data cut: all screening and postbaseline data with a clinical date (i.e., administration/assessment/onset/start date) the day before the date of re-randomization to Part 2 (Week 56).<br>The Part 1 data cut included all data regardless of the type of study visit at which it was collected. This may include data collected at prior to start dopaminergic visits, unscheduled visits, dosing termination visits, early termination visits, or safety follow-up visits, if the visit date was before re-randomization to Part 2 (Week 56). The trial was conducted at 57 sites in Austria, France, Germany, Spain, and the United States. The trial started on 27 June 2017 (i.e., first participant randomized). The last participant's last visit during Part 1 of the trial was on 27 November 2019.
Outcomes	The results of the subpopulations analyses of the following secondary endpoints in PASADENA Part 1 (randomized controlled part of the study) are reported: MDS-UPDRS Parts I, II and III. Following the International Council for Harmonization of Technical Requirements for Pharmaceuticals for Human Use (ICH) E9 (R1) addendum, analyses were performed using two estimand strategies to handle the post-randomization event of start or increase of symptomatic treatment: (1) 'hypothetical strategy', the estimated treatment effect assumes a scenario in which the events of start of symptomatic therapy or change in MAO-B inhibitor dose did not

March 2021

occur (performed for the mITT population), and (2) 'treatment policy', an assessment of treatment effect irrespective of symptomatic treatment start or changes in MAO-B inhibitor treatment (performed for the ITT population). The hypothetical strategy implies that the data following the first dose of symptomatic treatment or change in MAO-B inhibitor dose are excluded from the analysis; instead, the treatment effect from these participants is estimated through the covariance matrix of the mixed models for repeated measures (MMRM) model. For the treatment policy analysis of MDS-UPDRS Part III, all the data are included in the analysis, regardless of symptomatic treatment intake. Two scenarios are considered in this case: (1) measurements in practically-defined OFF state (i.e., 12 hours after withdrawal of levodopa), and (2) ON state (after taking levodopa).
In this manuscript we report subgroup analyses of the following secondary endpoints in PASADENA Part 1: MDS-UPDRS Parts I, II and III. All the subgroup analyses presented in the manuscript were pre-specified in the Statistical Analysis Plan (SAP; please see Section 4.7.5). This section describes the covariates used for the main subgroup analyses, and the exploratory subgroups can be found in Appendix 1.
